# Secondary Metabolites with Anti-Inflammatory Activities from One Actinobacteria *Amycolatopsis taiwanensis*

**DOI:** 10.3390/molecules26195765

**Published:** 2021-09-23

**Authors:** Yung-Shun Su, Ming-Der Wu, Jih-Jung Chen, Ming-Jen Cheng, Yueh-Hsiung Kuo, Chee-Yin Chai, Aij-Lie Kwan

**Affiliations:** 1 Graduate Institute of Medicine, College of Medicine, Kaohsiung Medical University (KMU), Kaohsiung 807, Taiwan; mariussu@gmail.com (Y.-S.S.); cychai@kmu.edu.tw (C.-Y.C.); 2 Department of Dermatology, Kaohsiung Medical University Chung-Ho Memorial Hospital, Kaohsiung 807, Taiwan; 3 Bioresource Collection and Research Center (BCRC), Food Industry Research and Development Institute (FIRDI), Hsinchu 300, Taiwan; wmd@firdi.org.tw; 4 Department of Pharmacy, School of Pharmaceutical Sciences, National Yang Ming Chiao Tung University (NYCU), Taipei 112, Taiwan; 5 Department of Medical Research, China Medical University Hospital, Taichung 404, Taiwan; 6 Department of Chemistry, National Taiwan University, Taipei 106, Taiwan; yhkuo800@gmail.com; 7 Department of Biotechnology, Asia University, Taichung 413, Taiwan; 8 Department of Chinese Pharmaceutical Sciences and Chinese Medicine Resources, College of Pharmacy, China Medical University, Taichung 404, Taiwan; 9 Department of Pathology, Kaohsiung Medical University Chung-Ho Memorial Hospital, Kaohsiung 807, Taiwan; 10 Ph.D. Program in Environmental and Occupational Medicine, College of Medicine, Kaohsiung Medical University and National Health Research Institutes, Kaohsiung 807, Taiwan; 11 Department of Neurosurgery, Kaohsiung Medical University Chung-Ho Memorial Hospital, Kaohsiung 807, Taiwan

**Keywords:** *Amycolatopsis taiwanensis*, Pseudonocardiaceae, actinobacteria, secondary metabolites, NO inhibition

## Abstract

Phytochemical investigation and chromatographic separation of extracts from one new actinobacteria strain *Amycolatopsis taiwanensis* that was isolated from soil of Yilan township, in the north of Taiwan, led to the isolation of nine new compounds, amycolataiwanensins A–I (**1**–**9**, resp.), and one *new natural product*, namely amycolataiwanensin J (**10**). The structures of the new compounds were unambiguously elucidated on the basis of extensive spectroscopic-data analysis (1D- and 2D-NMR, MS, and UV) and comparison with literature data. The effect of some isolates on the inhibition of NO production in lipopolysaccharide-activated RAW 264.7 murine macrophages was evaluated. Of the isolates, **3**, **5**, **7** and **8** exhibited potent anti-NO production activity, with *IC*_50_ values of 17.52, 12.31, 17.81 and 13.32 μM, respectively, compared to that of quercetin, an iNOS inhibitor with an IC_50_ value of 35.94 μM. This is the first report on indole metabolite from the genus *Amycolatopsis*.

## 1. Introduction

Actinobacteria are well known as an outstanding and fascinating source of commercially valuable bioactive compounds, particularly antibiotics. Almost a half of the known microbial bioactive secondary metabolites are derived from actinomycetes, of which more than 70% were obtained from the genus *Streptomyces*. However, the active ingredients of many new Taiwanese actinobacteria and their mechanisms of actions are still unknown. It is necessary to study on the active compounds by scientific methods from these rare actinobacteria. Actinobacteria are widely distributed in nature. They seem to have unlimited ability to produce secondary metabolites with multiple chemical structures and biological activities, so they have a place in the pharmaceutical industry [1,2,3,4,5]. They are Gram-positive, free-living saprophytic bacteria that exist in soil, water and colonizing plants. The residents of actinobacteria have been identified as one of the main groups of soil populations [4], may vary depending on the type of soil.

Based on our long-term plan for the collection and identification of new species of native actinobacteria in Taiwan, we isolated several new strains from Taiwan soil. A strain named 0345M-7T was isolated from soil sediment samples in Yilan County, Taiwan. It has a unique morphology after observation through an electron microscope [4].

This strain was determined to be *Amycolatopsis taiwanensis*, (Family: Pseudonocardiaceae) based on their phenotypic and genotypic data [5]. The isolate displayed substrate mycelia, upon which were borne short spore chains. The spore chains were composed of non-motile, smooth-surfaced, oval spores. The genus *Amycolatopsis* (Lechevalier et al. 1986) contains more than 70 species and its main habitat is soil. Strains of this genus can produce a variety of important antibiotics and secondary metabolites, such as balimycin, dethymicin, rifamycin, vancomycin, and can be used for drug-resistant Staphylococcus, organ transplantation, leprosy, tuberculosis, etc. Although the strains of the genus *Amycolatopsis* have been researched and developed very early and many times, *A. taiwanensis* is a new strain isolated from Taiwan, and no relevant research has been carried out. It has the potential to discover new compounds. According to the literature search, it was found that 159 compounds of the genus *Amycolatopsis* were reported from 8 known species and 18 unknown species [6]. These secondary metabolites are mainly divided into polyphenols, linear polyketides, macrolides, macrolides, thiazolyl peptides, cyclic peptides, glycopeptides, amides and amino derivatives, glycoside derivatives, and enediyne derivatives, and sesquiterpenes. At the same time, they mainly exhibit unique antibacterial, anti-cancer, anti-oxidant, anti-hyperglycemic and enzyme inhibitory activities.

**Figure 1:**
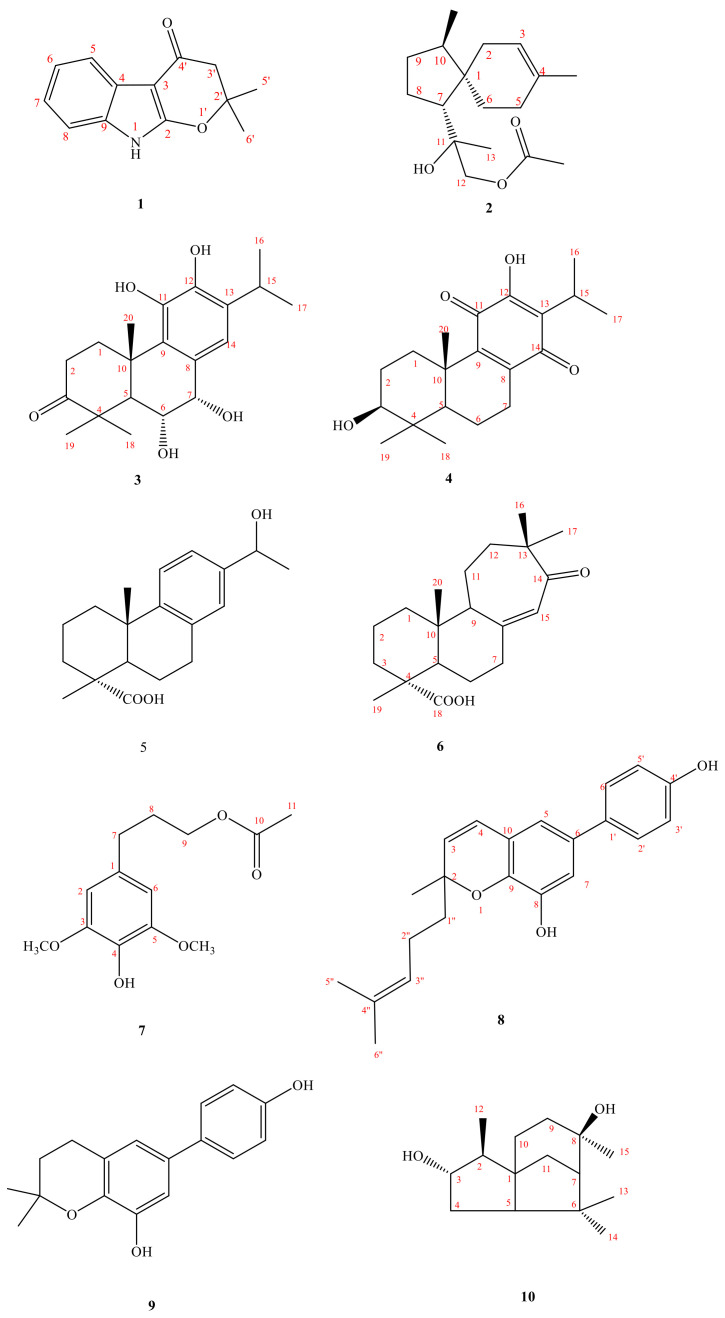
Compounds **1**–**10**, isolated from *Amycolatopsis taiwanensis*.

In the course of our search for potential diverse secondary metabolites from natural microbial sources, and to further understanding of the minor metabolites of the genus *Amycolatopsis*, we examined the EtOAc extract of *A. taiwanensis*, which showed rich metabolites according to the HPLC fingerprint analysis and inhibitory activity on LPS-induced NO release in RAW 264.7 murine macrophages, as determined by our primary screening. Investigation of the bioactive metabolites of the active EtOAc extract from the microbe *A. taiwanensis*, fermented by liquid fermentation was investigated. The metabolites investigation guided by the HPLC profile analysis and ^1^H-NMR spectrum prescreening led to the isolation of nine new metabolites, amycolataiwanensins A-I (**1**–**9**), and one metabolite isolated for the first time from nature sources, amycolataiwanensin J (**10**) (Figure 1 and the Supplementary part). The structures of these isolates were established by means of spectral experiments. The isolation, structural elucidation, inhibitory effects of some isolates on nitric oxide (NO) production by RAW264.7 macrophages are described herein.

## 2. Results and Discussion

**Figure 2:**
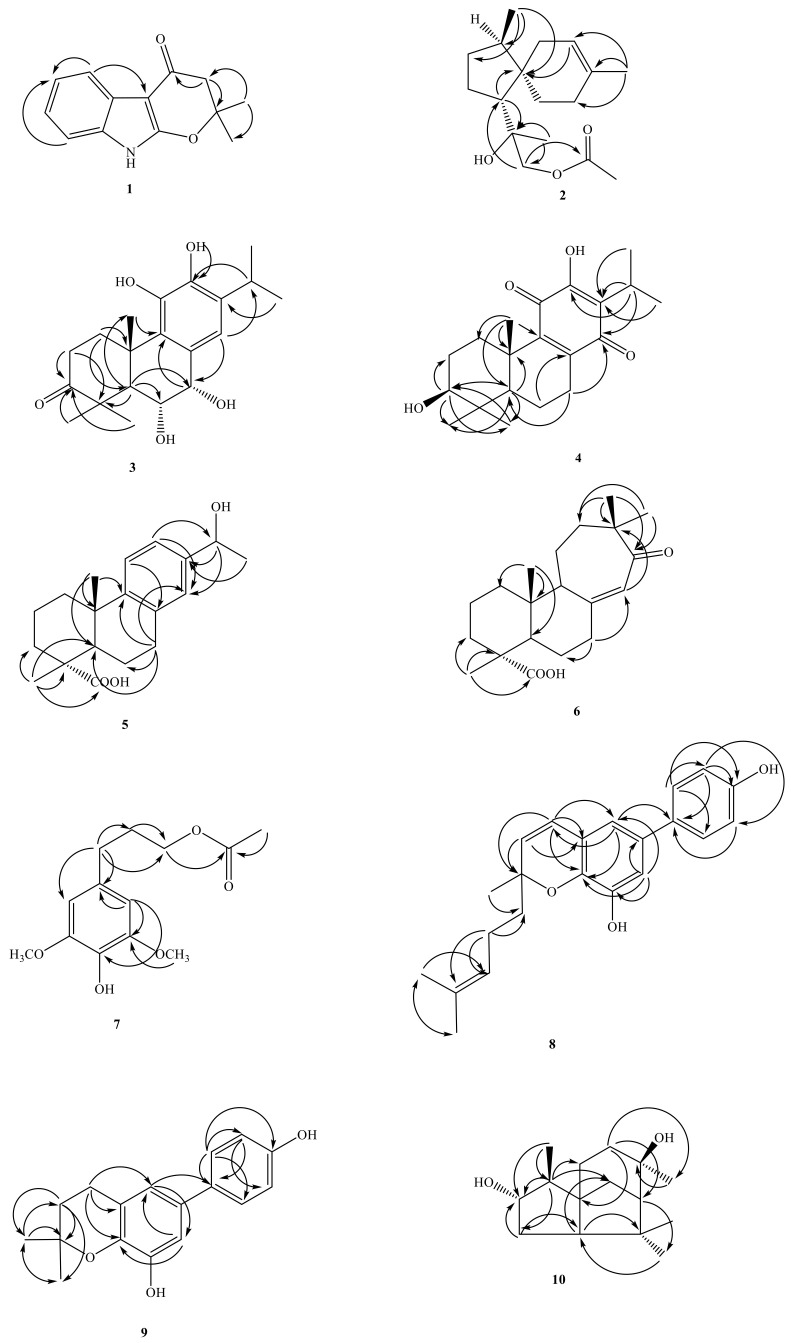
Key HMBC (→) correlations of **1**–**10**.

**Figure 3:**
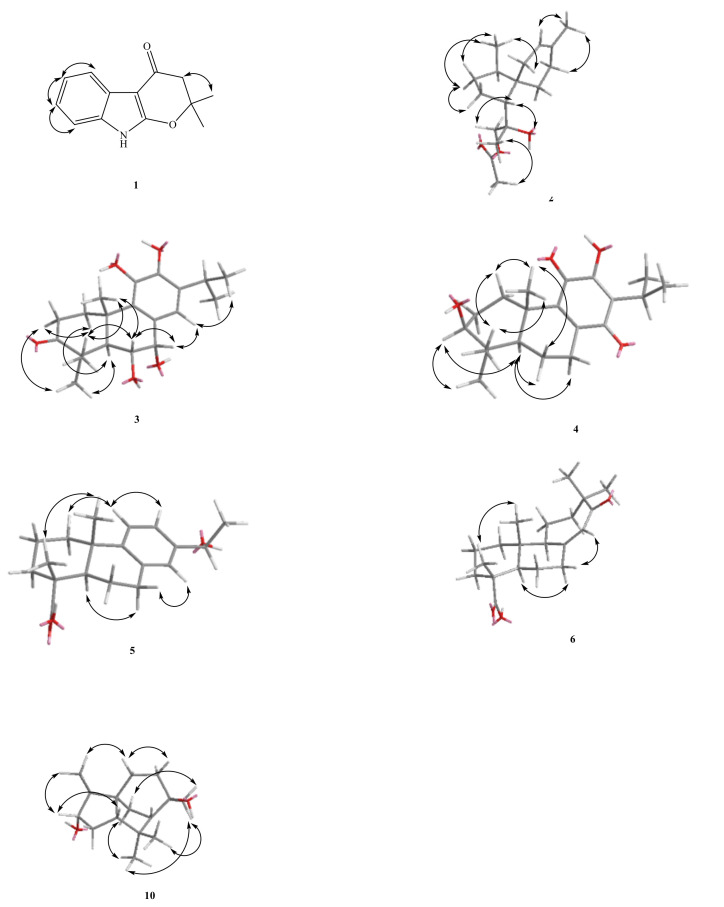
Major NOESY (↔) contacts of **1**–**6** and **10**.

Compound **1** was isolated as oil. The HR-EI-MS spectrum gave a molecule ion [M]^+^ at *m/z* 215.0948, consistent with a molecular formula of C_13_H_1__3_NO_2_. UV spectrum showed maximum absorption at 241 (4.20), 264 (4.09) and 310 (3.98) nm, indicating the presence of an indole skeleton [7]. Its IR spectrum revealed NH absorption at 3320 cm^−1^. Analysis of the ^1^H NMR spectrum of **1** revealed four typical mutually coupling aromatic protons of indole alkaloid at δ_H_ 7.08 (1H, td, *J* = 8.0, 1.2 Hz, H-7), 7.11 (1H, td, *J* = 8.0, 1.2 Hz, H-6), 7.26 (1H, dd, *J* = 8.0, 1.2 Hz, H-8), 7.84 (1H, dd, *J* = 8.0, 1.2 Hz, H-5) and one NH group at δ_H_ 7.66 (1H, br s, exchangeable with D_2_O). According to the molecular formula, the degree of unsaturation can be calculated to be 8. After deducting the two rings and four double bonds of indole, there are also 2 remains. In addition to the carbon spectrum, there is a conjugated carbonyl signal at δ_C_ 183.99. It is speculated that the branched chain forms a ring (C ring). It can be confirmed from the fact that the methylene signal on the carbonyl α carbon is δ_H_ 2.63 (2H, s) in a lower magnetic field. In addition, the two carbon absorption signals on the pyrrole ring of indole, a particularly low magnetic field and a high magnetic field, are presumably affected by the electronic resonance of the carbonyl group. Therefore, one end of the branch is connected to a carbon with a higher magnetic field (δ_C_ 95.15) through a carbonyl carbon. The carbon with a lower magnetic field (δ_C_ 160.87) should also be connected to an oxygen and be affected by the carbonyl group to achieve such a low magnetic field. Observing the carbon spectrum, there is also a quaternary carbon connected to oxygen. It is obvious that the other end of the branch chain is connected to indole with this oxygen. There is also a single peak integrated into 6H in the ^1^H NMR, δ_H_ 1.57 (6H, s, CH_3_-5′, 6′), which is presumed to be two methyl groups with the same signal, and the position is on the quaternary carbon (δ_C_ 86.2) connected to oxygen. The HMBC ^3^J-correlations (Figure 2) from δ_H_ 2.63 (CH_2_-3′) to δ_C_ 95.2 (C-3) and one weak ^4^J-correlations δ_H_ 1.57 (6H, s, CH_3_-5′, 6′) to δ_C_ 160.9 (C-2), verify the junction of the 2,2-dimethyldihydropyrano ring to the indole moiety at C-2, and 3. The other key correlations of HMBC are illustrated in Figure 2. Based on the above data, the structure of **1**, named amycolataiwanensin A, was elucidated as 2,2-dimethyl-2,3-dihydropyrano[2,3-*b*]indol-4(9*H*)-one, which was further confirmed by ^13^C NMR, COSY, NOESY (Figure 3), HSQC and HMBC (Figure 2) experiments.

Compound **2**, isolated as gum, showed a dehydrated molecular ion [M-H_2_O]^+^ peak at *m/z* 262.1933 for C_17_H_26_O_2_, corresponding to four indexes of hydrogen deficiency (IHD). The IR spectrum of **2** displayed an absorption for an OH group (3500 cm^−1^) and a C = O group (1740 cm^−1^). The ^1^H- and ^13^C-NMR, COSY, HMBC, and NOESY data (Figure 3) established the structure of **2** as 12-acetoxy-11-hydroxyacora-3-ene.

The ^1^H-NMR spectrum exhibited signals for a trisubstituted olefinic proton [δ_H_ 5.30 (br s, H-3)], an oxymethylene at δ_H_ 3.93/4.05 (each d, *J* = 11.0 Hz, CH_2_-12), and the four methyl groups include three singlet methyl groups δ_H_ 1.24 (s), 1.58 (s), 2.08 (s) and a doublet methyl group at δ_H_ 0.82 (d, CH_3_-14). From the carbon spectrum (^13^C-NMR) and DEPT, because of the appearance of one quaternary carbon at δ_C_ 171.2 (ester) and one primary carbon at δ_C_ 20.9 (CH_3_), it can be seen that there is an acetoxy group. In addition, there are two quaternary carbons δ_C_ 44.70 and 74.85 (of which δ_C_ 74.35 is the oxygen-containing quaternary carbon). Calculating the degree of unsaturation, subtracting a double bond and a carbonyl group, there is 2 left, so it can be determined that there are two more rings in this structure. According to the above characteristic spectrum information and reference data, [8] it can be known that the NMR information of this structure are very similar to the spectrum of a known compound 12-acetoxy-11-hydroxyacora-4-ene, so it is inferred that this compound It is also belonged to acorane backbone compound. According to the signal of HMBC, the structure is similar to the known compound 12-acetoxy-11-hydroxyacora-4-ene, [8] while the signal of NOESY explains the difference in stereo orientation: (1) The H-2 has NOE correlation signals with H-3 and H-14 in compound **2**. (2) In the similar compound 12-acetoxy-11-hydroxyacora-4-ene, its H-6 has NOE correlation signal with H-13 and H-5. Therefore, the structure and steric orientation of compound **2** were proved. The above spectroscopic data proves that the structure is correct, and this new compound is named amycolataiwanensin B.

Compound **3** had the dehydrated molecular ion peak [M-H_2_O]^+^ at *m/z* 330.1831 (HR-EI-MS), as analyzed for C_20_H_28_O_5_. The IR spectrum of **3** exhibited the presence of an OH group at 3400 cm^−1^ and a CO moiety at 1695 cm^−1^. The UV absorptions (λ_max_ 229.0 and 256.0 nm) confirmed an aromatic system. Seven IHD were determined from the molecular formula, ^13^C-NMR spectrum, and DEPT. Further spectral data (Table 1 and Table 2) and comparison with reference compounds [9] established the structure of **3** as 6α,7α,11-trihydroxy-3-oxoferrugiol. The ^1^H-NMR spectrum of **3** indicated the presence of an iPr group (δ_H_ 1.27 (d) and 1.28 (d), and 2.99 (sep)) attached to the benzene ring, two phenol groups (δ 4.85 (s), 5.81 (s)), another three Me groups (δ_H_ 1.21 (s), 1.33 (s), and 1.45 (s)) attached to a quaternary carbon, two OCH groups (δ_H_ 4.96 (d, 5.0) and 4.44 (dd, 11.5, 5.0)), the signal of five substituted benzene ring at 6.81 (s, H-14). According to the ^13^C-NMR and DEPT, the benzene ring δ_C_ 125.33, 130.44, 139.96, 142.42, 132.91 and 119.70 composed of 6 olefinic carbons; there is a carbonyl group at δ_C_ 219.66, and two oxygen-containing tertiary carbons at δ_C_ 68.3 (C-7), and 74.3 (C-6). Based on the above information and combining the above characteristic spectra data, the compound **3** with the abietane skeleton can be identified. The H-1β at δ_H_ 3.03 (m) is the result of the displacement of the low magnetic field due to the influence of the hydroxyl group on C-11. In order to determine the structure and the position of each functional group, continue with two-dimensional nuclear magnetic resonance spectroscopy (HSQC, HMBC) and NOESY experiments. According to the key information of HMBC: (1) H-18 and H-19 are correlated to δ_C_ 219.66, so it is determined that the carbonyl group is located at the position of C-3; (2) H-14 is only related to δ_C_ 68.26, so the two tertiary hydroxyl carbons can be distinguished. (3) Because δ_H_ 5.81 (s, OH-12) is correlated to C-12, the two phenols can be distinguished and make sure that δ_H_ 5.81 (s) is connected to C-12. According to the signal from NOESY: (1) H-5 is connected with H-1α and H-18 respectively; (2) H-6 is connected with H-19 and H-20, so it is determined to be in the axial position; (3) H-7 is related to H-6β and H-14, and by its coupling constant (*J* = 5.0 Hz), it can also be determined that it is in the equatorial position. Compound **3** is a previously undescribed diterpene and was named amycolataiwanensin C.

The ^1^H-NMR signals of **4** at δ_H_ 0.84 (s), 1.02 (s) and 1.22 (s); 1.19 and 1.18 (each 3H, d, *J* = 7.0 Hz, CH_3_-16, 17), and 3.12 (H-15, COSY cross-peaks with δ_H_ 1.19 and 1.18) suggested that **5** has an iPr group and three Me groups attached to a quatenary C-atom. According to ^13^C-NMR and DEPT, in addition to isopropyl and three singlet methyl groups, there is a carbon at δ_C_ 78.24, which is a tertiary carbon connected to oxygen, and δ_C_ 183.25 and 187.31 show quinone group signals. In addition, there are four olefinic carbons, δ_C_ 123.85, 150.56, 145.74 and 145.97. Since the compound is yellow and the UV absorption spectrum shows, coupled with the above-mentioned spectral data (^1^H-NMR and ^13^C-NMR data of known compounds in Reference [10], it can be inferred that compound **4** is a derivative of hydroxybenzoquinone in the abietane skeleton. The C ring is a quinone ring, and H-1β (δ 2.79) is affected by the quinone group of C-11, so the magnetic field is relatively low. From the signal of δ_H_ 3.24 (dd, J = 10.6, 5.7 Hz), it can be seen that this H is in the axial position, and -OH is in the equatorial position. By heteronuclear correlation spectroscopy (HSQC, HMBC) and NOESY to analyze its structural correlation and stereo orientation. According to HMBC’s information as following: (1) δ 3.24 is related to C-18 and C-19 respectively, so it is determined that the hydroxyl group is connected to the position of C-3; (2) H-5 is connected to C-3, C-7, and C-3, respectively. C-10, C-18, C-19, C-20 are connected; (3) H-15 is connected with C-12, C-13, C-14 respectively; (4) H-20 is connected with C-1. C-5, C-9, C-10 are related. According to the signal from NOESY: (1) H-5 is related to H-1α, H-3, H-6α, H-7α, and H-18 respectively; (2) H-3 is related to H-5 and H-18 Therefore, it is determined that H-3 is in the axial direction; (3) H-20 is related to H-2β and H-6β; (4) H-2β is related to H-19 and H-20. The structure was proved to be correct, and the new compound was named amycolataiwanensin D.

Compound **5** was obtained as a colorless gum and had a molecular formula of C_19_H_26_O_3_ by the HR-EI-MS (*m/z* 302.1885 [M+H]^+^, calcd for C_20_H_30_O_3_ 302.1882), requiring six degrees of unsaturation. The IR spectrum of **5** displayed absorption characteristic of a hydroxy (3396 cm^−1^), carboxylic acid group (2700~3400 (OH) & 1697 (C = O) cm^−1^), and benzene ring (1570 and 1498 cm^−1^). The ^13^C NMR spectrum revealed the presence of 20 carbon signals, which were assigned with the assistance of DEPT spectrum as one sp^2^ quaternary carbonyl carbon [δ_C_ 184.5 (C-18)], three sp^3^ methyls [δ_C_ 24.8, 16.2, and 25.0 (C-17, 19 & 20)], five sp^3^ methylenes [δ_C_ 37.8, 18.5, 36.6, 21.6, and 29.9 (C-1, 2, 3, 6 & 7)], two sp^3^ methines [δ_C_ 44.5, and 70.1 (C-5 and 15)], three sp^2^ methines [δ_C_ 124.4, 122.8, and 126.0 (C-11, 12, and 14)], two sp^3^ quaternary carbons [δ_C_ 47.3 and 37.0 (C-4 and 10)], and five sp^2^ quaternary carbons [δ_C_ 135.1, 148.6, and 142.6 (C-8, 9, and 13)]. The ^1^H-NMR and ^13^C-NMR spectra (Table 1 and Table 2) of **5** were similar to those of 12-methoxy-13-(1-hydroxy ethyl)podocarpa-8,11,13-trien-19-oic acid [11,12], except that an aromatic proton [δ_H_ 7.11 (d, *J* = 8.2 Hz, H-12)] of **5** replaced a methoxyl group at C-12 [δ_H_ 3.65 (3 H, s, OCH_3_)] of **5a**. According to the DEPT-NMR spectrogram, there are three primary carbons, five secondary carbons, five tertiary carbons, and six tertiary carbons. The degree of unsaturation is estimated to be 7, which is consistent with the predicted structure. Viewed from the HMBC spectrum, δ_H_ 1.48 (H-16) is correlated to C-15 and C-13, and δ_H_ 4.80 (H-15) is correlated to C-16, C-14, C-13, and C-12, could be explained the position of the doublet methyl group (Me-15) and the hydroxyl group. The stereochemistry is determined from the NOESY spectrum. H-20 and H-19 have NOESY correlation, which is sufficient to show that -COOH is located in the equatorial. H-1β has NOESY association with H-11, and H-16 has NOESY association with H-12 and H-14, which are consistent with the speculated structure. Based on the above data, it can be determined that compound **5** is (1R*,4aS*)-7-(1-hydroxyethyl)-1,4a-dimethyl-1,2,3,4,4a,9,10,10a-octahydrophenanthrene-1-carboxylic acid (15-hydroxyabieta-8,11,13-trien-18-oic acid) and named as amycolataiwanensin E.

Compound **6** was isolated as oil. Its molecular formula, C_20_H_30_O_3_, was determined on the basis of the positive HR-EI-MS at *m/z* 318.2197 [M]^+^ (calcd 318.2195) and was supported by the ^1^H, ^13^C, and DEPT data. The IR absorption bands of **6** revealed the presence of the COOH (3400 cm^−1^ for OH; 1699 cm^−1^ for CO) and a conjugated carbonyl (1670 cm^−1^) functions. According to DEPT plots, there are four primary carbons, seven secondary carbons, three tertiary carbons, and six quaternary carbons. The degree of unsaturation is estimated to be 6. In ^13^C-NMR spectrum, four signals at δ 29.1, 23.8, 16.5, and 14.6, and ^1^H-NMR at δ 0.67, 1.01, 1.13, & 1.14 (each 3H, s), showing the presence of four Me groups. The ^13^C-NMR spectrum at δ 183.8 (s) and the infrared absorption spectrum at 2700–3400, 1699 cm^−1^, it shows that this compound has a carboxylic acid group. In addition, δ 154.2 (s), 125.1 (d) and ^1^H-NMR spectrum δ 5.82 (1H, br s) show that there is a group of triple-substituted double bonds. The remaining ^13^C-NMR spectrum at δ 211.2 (s) shows the existence of carbonyl group (C = O). It can be inferred that **6** contains a carboxylic acid, a set of double bonds, and a carbonyl group. The remaining unsaturation is 3, which is inferred to be a tricyclic structure. HMBC correlations of δ_H_ 1.01 (H-17)/δ_C_ 49.5 (C-15), 211.2 (C-13); δ 1.13 (H-16) δ 49.5 (C-15), 211.2 (C-13); δ 5.82 (H-14)/δ 49.5 (C-15). From the HMBC spectrum, δ 1.01 (H-17) is correlated to δ 49.5 (C-15), 211.2 (C-13); δ 1.13 (H-16) is correlated to δ 49.5 (C-15), 211.2 (C-13), and the correlation between δ 5.82 (H-14) and δ 49.5 (C-15), and the double bond signal will shift to a low magnetic field. It is speculated that the double bond is conjugated to the carbonyl group, but δ 211.2 (C-13)) Unlike a conjugated carbonyl group, there may be one α carbon and three β carbons, which makes the carbonyl shift to a lower magnetic field. From the HMBC correlations between δ2.07 (H-9) and δ 39.7 (C-10), 14.6 (C-20); δ 1.98 (H-5) and δ 47.1 (C-4), 39.7 (C-10), 26.1 (C-6), 16.5 (C-19), 14.6 (C-20); δ 1.14 (H-19) and δ 183.8 (C-18), 47.1 (C-4), 36.9 (C -3); δ 5.82 (H-14) and δ 154.2 (C-8), 49.5 (C-15), 38.6 (C-7); δ 1.58 (H-12) and δ 211.2 (C -13), 59.7 (C-9), 23.8 (C-16); δ 1.13 (H-16), 1.01 (H-17) are related to δ 211.2 (C-13), 49.5 (C-15), 35.4 (C-12) respectively. From the above analysis of 1D and 2D spectra, compound **6** is 12(13→15)abeoabietane diterpenes, C-12 is not connected to C-13, and C-15 is reversed to cause the six-ring to seven-ring, geminal dimethyl group is substituted for isopropy. As for its proposal biosynthesis and the stereochemistry, it can be explained by the NOESY spectrum. H-20 and H-19 have a NOESY correlation, which is sufficient to show that -COOH is located in the equatorial direction. The spectra of HMQC and COSY confirm that the compound **6** is 12(13→15)abeo-13-oxo-8(14)-abietene-18-oic acid and designated as amycolataiwanensin F.

Compound **7** was obtained as oil. Its molecular formula C_13_H_18_O_5_ was deduced from molecular ion at *m/z* 254.1151 [M]^+^ (calcd 254.1154) in the HR-EI mass spectrum. The presence of hydroxyl (3440 cm^−1^), acetoxyl (1730 cm^−1^), and benzene (1615 and 1518 cm^−1^) groups were evident from the IR spectrum. The ^1^H (Table 1) and ^13^C NMR (Table 2) data of **7** were very similar to those of dihydrosyringenin [13], except that an acetoxy group [δ_H_ 2.02 (3H, s); δ_C_ 20.9, 171.1 (OCOCH_3_)] at C-9 in **7** replaced the hydroxy group of dihydrosyringenin. This was supported by HMBC correlation between OCH_2_-9 (δ_H_ 3.76) and C-11 (δ_C_ 171.1) (Figure 2). The full assignment of ^1^H and ^13^C NMR resonances was supported by ^1^H-^1^H COSY, DEPT, HSQC, NOESY, and HMBC (Figure 2) spectral analyses. Thus, the structure of **7** was established as shown in Figure 1, and named amycolataiwanensin G.

Compound **8** was isolated as oil. Its molecular formula, C_17_H_18_O_3_, was determined on the basis of the HR-EI-MS at *m/z* 270.1252 [M]^+^ (calcd 270.1256) and was supported by the ^1^H, ^13^C, and DEPT. The IR absorption bands of **8** revealed the presence of hydroxyl (3400 cm^−1^) and benzene (1614 and 1520 cm^−1^) functions. The ^1^H (Table 1) and ^13^C NMR (Table 2) data of **8** were similar to those of oblongifoliagarcinine A, [14] except that a 4-methylpent-3-en-1-yl group [δ_H_ 5.08 (1H, t, *J* = 7.1 Hz, H-3″), 1.59 (3H, s, CH_3_-6″) 1.64 (3H, s, CH_3_-5″), 2.09 (2H, m, H-2″), 1.75 (2H, m, H-1″); δ_C_ 22.8 (C-2″), 123.8 (C-3″), 131.9 (C-4″), 25.6 (C-6″), 17.6 (C-5″), 41.7 (C-1″)] at C-2 in **8** replaced the Me group at C-2 of oblongifoliagarcinines A [14]. This was supported by NOESY correlations between Me-2 (δ_H_ 1.42) and H-2′′ (δ_H_ 2.09), and between H-3″ (δ_H_ 5.08) and both of CH_3_-6″ (δ_H_ 1.64) and H-1′’ (δ_H_ 1.75) and by HMBC correlation between H-1′’ (δ_H_ 1.75) and C-3 & 9 (δ_C_ 129.8 & 138.5) (Figure 2). The full assignment of ^1^H and ^13^C NMR resonances was further confirmed by DEPT, ^1^H-^1^H COSY, NOESY, HSQC, and HMBC data (Figure 2). Consequently, the structure of compound **8** was established as amycolataiwanensin H.

Compound **9** was obtained as oil and had the molecular formula C_22_H_24_O_3_, as inferred from the HR-EI-MS showing the molecular-ion peak at *m/z* 336.1728 [M]^+^, indicating nine degrees of unsaturation. The IR spectrum of **9** showed absorption bands at 3395 cm^−1^ for free OH groups, 1613, 1596, 1520, 1486 cm^−1^ for aromatic moieties. The ^1^H-NMR and ^13^C-NMR spectra (Table 1 and Table 2) of **9** were similar to those of oblongifoliagarcinines A [14], except that the single bond at C3–C4 [δ_H_ 2.78 (2H, t, *J* = 6.7 Hz, H-4), 1.83 (2H, t, *J* = 6.7 Hz, H-3)] of **9** replaced a pair of double bond [δ_H_ 5.65 (2H, d, *J* = 9.8 Hz, H-4), 6.37 (2H, d, *J* = 9.8 Hz, H-3)] of oblongifoliagarcinines A [14]. The ^1^H-and ^13^C-NMR (Table 1 and Table 2) and HMBC data (Figure 2) established the structure of amycolataiwanensin I (**9**) as 6-(4-hydroxyphenyl)-2,2-dimethylchroman-8-ol.

Fifteen ^13^C-NMR signals and the HR-EI-MS confirmed the molecular formula C_15_H_26_O_2_ of **10**. Analysis of its IR spectrum suggested that **10** contained OH (3299 cm^−1^) moiety. The three IHD (from the DEPT experiment), the ^13^C-NMR data, and the molecular formula indicated that **10** is a sesquiterpene. Further spectral data established the structure of **10** as (2S*,3S*,6R*)-3,6,8,8-tetramethyloctahydro-1*H*-3a,7-methanoazulene-2,6-diol (3α-hydroxycedrol). The ^1^H-NMR shows that δ_H_ 0.94 (d) is a doublet methyl group attached to a tertiary carbon. At δ_H_ 1.00 (s), 1.23 (s), and 1.32 (s), there are three singlet methyl groups on the quaternary carbon. Among them, δ_H_ 1.23 (s) and 1.32 (s) are located in the lower magnetic field because they are connected to the hydroxyl group. From ^13^C-NMR, DEPT, and HSQC plots, there are two carbons attached to oxygen at δ_C_ 72.84 and 81.43, 72.84 belongs to the quaternary carbon, and δ_C_ 81.43 belongs to the tertiary carbon. Calculating the degree of unsaturation, because there is no double bond or carbonyl carbon, it can be proposed this compound is a tricyclic ring. Based on the above spectral data, it is speculated that the compound should be a cedrane skeleton. After comparing with the reference data, and comparing with the ^1^H-NMR and ^13^C-NMR of the known compound cedrol [15], there is only one more oxygen-containing tertiary carbon. It can be confirmed by the signal (δ_H_ 3.58 (1H, ddd, *J* = 15.5, 10.5, 5.5 Hz, H-3); δ_C_ 81.4) appeared on the ^1^H-NMR & ^13^C-NMR. Continue to perform two-dimensional heteronuclear correlation spectroscopy (HSQC, HMBC) and NOESY to further determine the structural relevance and stereo orientation of **10**. The ^1^H signal at δ_H_ 1.45 (H-2)/δ_H_ 1.36 (CH_2_-4) and δ_H_ 0.94 (CH_3_-12) showed a two and three-bond connectivities with C-3 (δ_C_ 81.4) in the HMBC plot (Figure 2), which suggested that the second OH group at C-3. According to the signals from NOESY, determine the relative configuration of the C-3 and C-8 hydroxyl groups: (1) Compound **3** exhibited the HMBC correlation: H-3/12-Me, and judging from the split pattern (ddd), H-3 is located in the β-axial position; (2) 15-CH_3_ is correlated to H-9α and H-9β, so it is judged that 15-CH_3_ is equatorial. The structure was proved to be correct, and compared with the literature, it was confirmed that this was a compound discovered for the first time in nature, named amycolataiwanensin J.

NO is a mediator in the inflammatory response involved in host defense. In the course of our search for potential diverse secondary metabolites from natural fungal sources, and to further understanding of the bioactive metabolites of the genus *Amycolatopsis*, we examined the EtOAc extract of *A*. *taiwanensis*, which showed inhibitory activity on LPS-induced NO release production in RAW 264.7 murine macrophages, as determined by our primary screening (approximately 95% inhibition at a concentration of 10 μg/mL). Investigation of the bioactive metabolites of the active EtOAc extract from the titled material *A*. *taiwanensis*, led to the isolation of ten new compounds. Due to the small quantity of isolated compound (**1**), we evaluated the inhibitory effects of amycolataiwanensins B–J (**2**–**10**, resp.) on the production of NO induced by LPS. The inhibitory activity data of the 10 isolated compounds on NO generation by macrophages are shown in Table 3. From the results of our anti-inflammatory tests, the following conclusions can be drawn: (*a*) They showed potent inhibition with IC_50_ values between 12.8 to 34.2 μM, against lipopolysaccharide (LPS)-induced nitric oxide (NO) generation. (*b*)The high cell viability (>80%) indicated that the inhibitory activity of LPS-induced nitrite production by compounds **3**, **5**, **8**, and **9** (IC_50_ value: 17.52, 12.31, 17.81 and 13.32 *μ*M) did not result from its cytotoxicity. (*c*) Compounds **6** & **7** (IC_50_ value: 24.83 and 12.78 *μ*M) also showed inhibition of NO production of macrophages, but the low cell viability (<80%) suggested the possibility of cytotoxicity. (*d*) The sesquiterpene derivative, compound **2** exhibited less effective NO inhibition. (*e*) Among the abietane diterpene analogues, compound **3** (with (abietane with 3-isopropylbenzene-1,2-diol unit in C ring) exhibited more effective inhibition than its analogue, compound **4** (abietane with hydroxybenzoquinone unit in C ring), compound **5** (with 1-phenylethan-1-ol moiety in C ring) and compound **6** (with 7,7-dimethylcyclohept-2-en-1-one moiety in C ring. (*f*) Among the aromatics analogues, compound 7 (simple aromatic with 9-acetoxydihydrosyringenin) displayed better inhibition than its analogue, compound **8** (abietane with hydroxybenzoquinone unit in C ring), compound **5** (with 1-phenylethan-1-ol moiety in C ring) and compound **9** (with 7,7-dimethylcyclohept-2-en-1-one moiety in C ring. (*g*) Furthermore, the RT-PCR analysis in the present study indicated that LPS treatment increased the level of iNOS mRNA expression, and that compounds **3**, **5**, **8** and **9** inhibited this increase in a concentration-dependent manner. At the highest concentration, none of the compounds tested showed any obvious cytotoxicity toward RAW 264.7 cells. (*h*) Cytotoxic effects were measured using MTT assay. The high cell viability (>95%) indicated that the inhibitory activities of LPS-induced NO production by active compounds **3**, **5**, **8**, and **9** were not resulted from its cytotoxicity.

## 3. Materials and Methods

### 3.1. General Experimental Procedures

The following instruments were used for obtaining physical and spectroscopic data: optical rotations *Jasco DIP-370* polarimeter; in CHCl_3_ (JASCO, Kyoto, Japan); FTIR spectra were obtained by using a FTIR spectrometer (*Perkin-Elmer-2000* FT-IR spectrophotometer; ν in cm^−1^, Norwalk, CT, USA); Absorption spectra were recorded by an ultraviolet visible (UV-vis) light spectrophotometer (*Jasco UV-240* spectrophotometer; λ_max_ (log ε) in nm, Hitachi, Ltd., Tokyo, Japan), EI and HREIMS: Jeol JMS-HX-300 mass spectrometer; in *m/z* (rel. %) and JEOL SX-102A Mass Spectrometer; melting point, MP-J3 (Yanaco, Kyoto, Japan); NMR spectra were taken on a *Varian*-*Mercury*-500 and *Varian*-*Unity-Plus*-400 spectrometers with TMS as an internal standard (Lake Forest, California USA). Silica gel column chromatography was performed on silica gel (70–230 mesh, Merck, Darmstadt, Germany). HPLC was performed on a Shimadzu LDC-Analytical-III apparatus equipped with an UV–VIS detector (SPD–10A). A Spherical C18 column (250 × 10 mm, 5μm) was used for preparative purposes (flow rate: 2.00 mL/min). Aluminum pre-coated silica gel (Merck, Kieselgel 60 F254) were used for TLC monitoring with visualization by spraying with a 10% solution of Ce_2_SO4 in ethanol and heating to approximately 100 °C on a hotplate.

### 3.2. Microorganism

*Amycolatopsis taiwanensis* (0345M-7^T^) was used throughout this study, and deposited at Bioresource Collection and Research Center (BCRC), Food Industry Research and Development Institute (FIRDI). This actinobacteria was identified by Min Tseng., and specimens (0345M-7^T^) deposited at the Bioresource Collection and Research Center (BCRC) of the Food Industry Research and Development Institute (FIRDI).

### 3.3. Cultivation and Preparation of the Fungal Strain

The actinobacteria, *Amycolatopsis taiwanensis* (0345M-7^T^), was isolated from a sediment collected from the northern area of Taiwan, by using HVY agar, and was then incubated at 45 °C for 7 days. This actinobacteria was identified by one of the authors (Mrs. Min Tseng), and specimens (0345M-7^T^) deposited at the Bioresource Collection and Research Center (BCRC) of the Food Industry Research and Development Institute (FIRDI). The strain was maintained on oatmeal agar and the spores or mycelia suspension were harvest with 20% (*v*/*v*) glycerol and stored at −20 °C. A mature slant culture of strain 0345M-7^T^ was inoculated into a 500 mL flask containing 100 mL of the seed medium consisting of 0.4% glucose, 0.4% yeast extract, and 1% malt extract (pH 7.3). After growing at 30 °C for 4 d on a rotary shaker (200 rpm), the aliquots (2 mL) of seed culture were transferred into a 500 mL flask containing 200 mL of production medium (Humic acid 1.0 g, Na_2_HPO_4_ 0.5 g, KCl 1.7 g, MgSO_4_7H_2_O 0.05 g, FeSO_4_·7H_2_O 0.01 g, CaCO_3_ 0.02 g, Yeast extract 1.0 g, Agar 20.0 g, Dist. Water 1.0 L, pH 7.4). After 21 days cultivation at 30 °C temperature on a rotary shaker (500 rpm), the culture filtrates (10 L) were obtained by filtering through filter paper.

### 3.4. Isolation and Characterization of Secondary Metabolites

The liquid fermentation (H_2_O) of *A*. *taiwanensis* (10 L) was extracted with EtOAc, and the EtOAc-layer (16.0 g) was subjected to CC (SiO_2_; hexane/acetone gradient) to get six fractions (*Frs. 1–6*). *Fr. 1* was subjected to MPLC (RP-18; MeOH/H_2_O 2:1) to produce 11 fractions, *Frs. 1.1–1.11*. *Fr. 1.2* yielded **4** (3.6 mg). *Fr. 1.11* was further purified with prep. TLC (hexane/CH_2_Cl_2_ 6:1) to obtain **1** (0.8 mg). *Fr.* *2* was subjected to MPLC (SiO_2_; hexane/acetone 6:1) to produce 14 fractions, *Fr.* *2.1–2.14*. *Fr.* *2.4* was subjected to MPLC (SiO_2_; hexane/acetone 4:1) to produce four fractions, *Fr.* *2.4.1–2.4.4*. *Fr.* *2.4.4* was further purified with prep. TLC (CH_2_Cl_2_/AcOEt 10:1) to obtain **5** (*4*.6 mg), **2** (3.1 mg), **3** (*R*_f_ 0.51; 0.4 mg), and **10** (*2.*7 mg). *Fr.* *3* was subjected to MPLC (RP-18; acetone/H_2_O 1:2) to produce seven fractions, *Frs.* *3.1–3.7*. *Fr.* *3.1* was subjected to MPLC (SiO_2_; CH_2_Cl_2_/acetone, 10/1) to obtain **8** (6.3 mg). *Fr.* *3.6* was subjected to MPLC (RP-18; MeOH/H_2_O 1:2) to produce **9** (*4*.1 mg), **6** (3.1 mg). *Fr.* *3.7* was further purified with prep. TLC (hexane/EtOAc 1:1) to obtain **7** (1.7 mg). *Frs.4* and *5* are combined together after TLC analysis and were subjected to MPLC (RP-18; MeOH/H_2_O 2.5:1) to produce five fractions, *Frs.* *4.1–4.5*. *Fr.* *4.2* was subjected to MPLC (SiO_2_; CH_2_Cl_2_/MeOH 5:1) to produce 5 fractions, *Fr.* *4.2.1–4.2.5*. *Fr.* *4.2.4* was further purified with HPLC (RP-18; MeOH/H_2_O 1:2) to obtain **5** (*t*_R_ 12 min; 2 mL/min; 4.7 mg).

Amycolataiwanensin A (**1**): oil; UV (MeOH): 241 (4.20), 264 (3.68), 310 (3.88) nm; IR (Neat): 3194 (NH), 1738 (C = O), 1633, 1614, 1590 (aromatic C = C) cm^−1^; ^1^H NMR (400 MHz, CD_3_COCD_3_): see Table 1; ^13^C NMR (100 MHz, CD_3_COCD_3_): see Table 2); HREIMS *m/z* 215.0948 [M]^+^ (calcd for C_1__3_H_1__3_NO_2_, 215.0946).

Amycolataiwanensin B (**2**): oil; [α${]}_{D}^{25}$ = +17.0 (*c* 0.48, CHCl_3_); IR (Neat): 3500 (OH), 1740 (ester C = O) cm^−1^; ^1^H NMR (500 MHz, CDCl_3_): see Table 1; ^13^C NMR (125 MHz, CDCl_3_): see Table 2); EIMS (70 eV) *m/z* (%): 262 [M−H_2_O]^+^(10), 202 (14), 132 (100), 119 (91), 57 (98); HREIMS *m/z* 262.1933 [M−H_2_O]^+^ (calcd for C_1__7_H_2__6_O_2_, 262.1930).

Amycolataiwanensin C (**3**): oil; [α${]}_{D}^{25}$ = −56.2 (*c* 0.075, CHCl_3_); UV (MeOH): 265 (3.86) nm; IR (Neat): 3399 (OH), 1694 (C = O),1609, 1450 (aromatic C = C) cm^−1^; ^1^H NMR (500 MHz, CDCl_3_): see Table 1; ^13^C NMR (125 MHz, CDCl_3_): see Table 2); EIMS (70 eV) *m/z* (%): 330 [M−H_2_O]^+^(100), 229 (86), 83 (75), 55 (64); HREIMS *m/z* 330.1822 [M−H_2_O]^+^ (calcd for C_16_H_16_O_4_, 330.1826).

Amycolataiwanensin D (**4**): oil; ${]}_{D}^{25}$ = −85.2 (*c* 0.17, CHCl_3_); UV (MeOH): 272 (4.26), 352 (3.64) nm; IR (Neat): 3516 (OH), 1637 (conjugated C = O) cm^−1^; ^1^H NMR (500 MHz, CDCl_3_): see Table 1; ^13^C NMR (125 MHz, CDCl_3_): see Table 2); EIMS (70 eV) *m/z* (%): 332 ([M]^+^, 100), 299 (31), 231 (15), 69 (26), 59 (22); HREIMS *m/z* 332.1980 [M]^+^ (calcd for C_20_H_28_O_4_, 332.1982).

Amycolataiwanensin E (**5**): oil; ${]}_{D}^{25}$ = +31.0 (*c* 0.11, CHCl_3_); UV (MeOH): 218 (3.60), 256 (2.90) nm; IR (Neat): 3396 (OH), 2700~3400 (COOH), 1697 (COOH), 1570, 1498 (aromatic C = C) cm^−1^; ^1^H NMR (500 MHz, CDCl_3_): see Table 1; ^13^C NMR (125 MHz, CDCl_3_): see Table 2); EIMS (70 eV) *m/z* (%): 302 ([M]^+^, 30), 287 (100), 269 (39), 241 (40), 197 (70); HREIMS *m/z* 302.1882 [M]^+^ (calcd for C_20_H_30_O_3_, 302.1883).

Amycolataiwanensin F (**6**): oil; ${]}_{D}^{25}$ = +9.2 (*c* 0.56 CHCl_3_); UV (MeOH): 241 (3.70) nm; IR (Neat): 3400 (COOH), 1691 (COOH), 1670 (conjugated C = O) cm^−1^; ^1^H NMR (500 MHz, CDCl_3_): see Table 1; ^13^C NMR (125 MHz, CDCl_3_): see Table 2); EIMS (70 eV) *m/z* (%): 318 ([M]^+^, 18), 152 (100), 137 (18), 121 (22), 109 (25); HREIMS *m/z* 318.2193 [M]^+^ (calcd for C_20_H_30_O_3_, 318.2195).

Amycolataiwanensin G (**7**): oil; UV (MeOH): 233 (3.80) nm; IR (Neat): 3440 (OH), 1730 (C = O), 1611, 1518 (aromatic C = C) cm^−1^; ^1^H NMR (500 MHz, CDCl_3_): see Table 1; ^13^C NMR (125 MHz, CDCl_3_): see Table 2); EIMS (70 eV) *m/z* (%): 254 ([M]^+^, 53), 194 (29), 167 (100), 151 (14); HREIMS *m/z* 254.1151 [M]^+^ (calcd for C_1__3_H_1__8_O_5_, 254.1154).

Amycolataiwanensin H (**8**): oil; ${]}_{D}^{25}$ = +13.5 (*c* 0.36, CHCl_3_); UV (MeOH): 258 (4.20) nm; IR (Neat): 3400 (OH), 1614, 1520 (aromatic C = C) cm^−1^; ^1^H NMR (500 MHz, CDCl_3_): see Table 1; ^13^C NMR (125 MHz, CDCl_3_): see Table 2); EIMS (70 eV) *m/z* (%): 270 ([M]^+^, 53), 248 (12), 214 (100), 188 (18). HREIMS *m/z* 270.1252 [M]^+^ (calcd for C_17_H_18_O_3_, 270.1256).

Amycolataiwanensin I (**9**): oil; UV (MeOH): 224 (4.20), 268 (3.69) nm; IR (Neat): 3391 (OH), 1613, 1520 (aromatic C = C) cm^−1^; ^1^H NMR (500 MHz, CDCl_3_): see Table 1; ^13^C NMR (125 MHz, CDCl_3_): see Table 2); EIMS (70 eV) *m/z* (%): 336 ([M]^+^, 22), 253 (100), 69 (5); HREIMS *m/z* 336.1728 [M]^+^ (calcd for C_22_H_24_O_3_, 336.1725).

Amycolataiwanensin J (**10**): oil; ${]}_{D}^{25}$ = +34.8 (*c* 0.01, CHCl_3_); IR (Neat): 3299 (OH); ^1^H NMR (500 MHz, CDCl_3_): see Table 1; ^13^C NMR (125 MHz, CDCl_3_): see Table 2); EIMS (70 eV) *m/z* (%): 238 ([M]^+^, 10), 220(10), 167 (100),149 (31), 107 (34); HREIMS *m/z* 238.1930 [M]^+^ (calcd for C_1__5_H_26_O_2_, 238.1932).

### 3.5. Determination of NO Production and Cell Viability Assay

Mouse macrophage cell line (RAW 264.7) was obtained from Bioresource Collection and Research Center (BCRC 60001) and cultured at 37 °C in Dulbecco’s Modified Eagle’s Medium (DMEM) supplemented with 10% fetal bovine serum (Gibco), 4.5 g/L glucose, 4 mM glutamine, penicillin (100 units/mL), and streptomycin (100 μg/mL) in a humidified atmosphere in a 5% CO_2_ incubator. The cells were treated with 10, 25, 50 μM natural products in the presence of 1 μg/mL LPS (lipopolysaccharide, Sigma-Aldrich, St. Louis, MO, USA) for 20 h. The concentration of NO in culture supernatants was determined as nitrite, a major stable product of NO, by Griess reagent assay [16], and cell viabilities were determined using the MTT assay as described previously [17].

## 4. Conclusions

Actinobacteria have the ability to produce a variety of physiologically active products, so they play a very important role in the food and pharmaceutical industries. Over the years, our team has also separated and collected actinomycetes resources from all over Taiwan and various environments. In addition to common *Streptomyces*, there are also many rare species of actinobacteria, and there are many new species. Based on the concept of “new species and new compounds”, it is expected that special compounds can be found from these new strains. In recent years, studies have also found that these new species of actinobacteria can produce many active secondary metabolites. In order to further explore the efficacy of different strains of actinobacteria and expand the application range of actinomycetes, therefore, this project uses one new species of actinobacteria that have not been studied in the past, and they are cultured, extracted, purified and identified with high-level and highly active anti-inflammatory compounds with solid rice, in order to improve the research of actinobacteria in my country level, and develop health products related to actinobacteria. Under the support of the Ministry of Economic Affairs, the Bioresource Collection and Research Center at Food Industry Research and Development Institute, has been dedicated to the research work on the collection, separation and preservation of bio-resource research in the past few years, and has constructed a complete *indigenous* strains resource bank in Taiwan. The applicant analyzed the active constituents from it, and obtained more than sixty active new compounds isolated from red yeast rice, endophytes, actinobacteria, and mushrooms, among which many new compounds have anti-cancer and anti-inflammatory effects. Most of them have been published [18,19,20,21,22,23]. According to those findings, we have proved that the exploration of bioactive compounds on indigenous strains in Taiwan is a research-worthy topic. Actinobacteria are well known as an outstanding source of commercially valuable bioactive compounds, particularly antibiotics. Many microbial bioactive metabolites are derived from actinomycetes (*Streptomyces* sp.). However, the metabolites of many new Taiwanese actinobacteria and their mechanisms of actions are still unknown. It is necessary to study on the bioactive by scientific methods from these rare actinobacteria. In summary, we have isolated and characterized nine undescribed derivatives, amycolataiwanensins A–J from an actinobacteria strain *Amycolatopsis taiwanensis* that was isolated from soilt of Yilan township, in the north of Taiwan. The relative configurations of new isolates were determined by comparing their optical activities with related derivatives and NOESY plots. Amycolataiwanensins C, E, H & I showed inhibitory activities against LPS-induced NO production in RAW 264.7.

The discovery of indole, sesquiterpenes, diterpenes, and chromenes derivatives from actinobacteria pointed toward the potential of endophytic or associated *Amycolatopsis taiwanensis* as alternative producer of indole, sesquiterpenes, diterpenes, and chromenes derivatives. The current results may encourage further investigations on the chemistry and bioactivity of flavan metabolites. These results also suggest that *Amycolatopsis* has distinct and diverse metabolites that arise under different fermentation conditions and soil-derived collections. It may therefore be possible to find more new bioactive natural products by searching *Amycolatopsis* species under special eco-environment. For the sake of better understanding the distribution of flavan acid analogs, the actinobacteria of the tilted research material and other special strains are worth examining for the presence of these secondary metabolites.

## Figures and Tables

**Table 1:** ^1^H NMR Data for Compounds **1**–**10** in CDCl_3_ (*δ* in ppm, *J* in Hz, 500 MHz in CDCl_3_).

No	1 ^a^	2	3	4	5	6	7	8	9	10
1			3.03 (m, H-2β) 2.05 (m, H-2α)	2.79 (dt, *J* = 13.6, 3.2, H-2β) 1.25 (m, H-2α)	1.49 (m, H-1α) 2.30 (d, *J* = 13.2, H-1β)	1.15 (m, H-1α) 1.63 (m, H-1β)				
2		1.80 (m, H-2β) 2.31 (m, H-2α)	2.78 (ddd, *J* = 15.0, 11.6, 7.2 H-2β) 2.33 (dd, *J* = 15.0, 3.6, H-2α)	1.72 (m)	1.72 (m)	1.52 (m, H-2α) 1.58 (m, H-2β)	6.38 (s)	1.42 (s)	1.36 (s)	1.45 (br d, *J* = 7.0)
3		5.30 (br s)		3.24 (dd, *J* = 10.6, 5.7)	1.71 (m)	1.72 (m, H-3α) 1.75 (m, H-3β)		5.58 (d, *J* = 9.9)	1.83 (t, *J* = 6.7)	3.58 (ddd, *J* = 15.5, 10.5, 5.5)
4								6.38 (d, *J* = 9.9)	2.787 (t, *J* = 6.7)	1.36 (m, H-4α) 1.84 (m, H-4β)
5	7.84 (dd, *J* = 8.0, 1.2)	1.90 (m, H-5β) 1.95 (m, H-5α)	2.68 (d, J = 11.5)	1.05 (d, J = 12.5))	2.20 (dd, J = 12.0, 1.8)	1.98 (dd, J = 12.5, 2.9)		6.71 (d, *J* = 2.1)	6.78 (d, *J* = 1.6)	1.71 (m)
6	7.11 (td, *J* = 8.0, 1.2)	1.62 (m, H-6β) 1.92 (m, H-6α)	4.44 (dd, *J* = 11.5, 5.0)	1.41 (ddd, *J* = 13.5, 12.5, 5.5, H-6β) 1.86 (dd, J = 13.5, 7.5, H-6α)	1.87 (m, H-6β) 1.54 (m, H-6α)	1.45 (m, H-6α) 1.54 (m, H-6β)	6.38 (s)			
7	7.08 (td, *J* = 8.0, 1.2)	1.77 (m)	4.96 (d, J = 5.0)	2.72 (dd, *J* = 17.5, 5.5, H-7β) 2.32 (ddd, *J* = 17.5, 12.5, 7.5, H-7α)	2.90 (m)	2.22 (td, *J* = 13.0, 5.6, H-7α) 2.30 (m, H-7β)	2.59 (t, J = 8.0)	6.97 (d, *J* = 2.1)	6.94 (d, *J* = 1.6)	1.53 (m)
8	7.26 (dd, *J* = 8.0, 1.2)	1.52 (m, H-8β) 1.68 (m, H-8α)					1.91 (m)			
9		1.11 (m, H-9α) 1.81 (m, H-9β)				2.07 (dd, *J* = 11.5, 5.2)	4.07 (t, *J* = 6.7)			1.62 (m, H-9α) 1.81 (m, H-9β)
10		1.85 (m)								1.33 (m)
11					7.20 (d, J = 8.2)	1.83 (m, H-11α) 1.14 (m, H-11β)				1.53 (m, H-11α) 1.66 (m, H-11β)
12		3.93 (d, *J* = 11.0, H-12β) 4.05 (d, *J* = 11.0, H-12α)			7.11 (d, J = 8.2)	1.50 (m, H-12α) 1.58 (m, H-12β)	2.02 (s)			0.94 (d, *J* = 7.0)
13		1.24 (s)								1.32 (s)
14		0.82 (d, *J* = 7.0)	6.81 (s)		7.01 (br s)	5.82 (br s)				1.00 (s)
15		1.58 (s)	2.99 (sept, J = 7.0)	3.12 (sept, J = 7.0)	4.80 (q,. *J* = 6.5)					1.23 (s)
16			1.27 (d, *J* = 7.0)	1.19 (d, *J* = 7.0)		1.13 (s)				
17		2.08 (s)	1.28 (d, *J* = 7.0)	1.18 (d, *J* = 7.0)	1.48 (d, *J* = 6.5)	1.01 (s)				
18			1.33 (s)	1.02 (s)						
19			1.45(s)	0.84 (s)	1.28 (s)	1.14 (s)				
20			1.21 (s)	1.22 (s)	1.19 (s)	0.67 (s)				
2′								7.38 (d, *J* = 8.7)	7.38 (d, *J* = 8.5)	
3′	2.63 (s)							6.84 (d, *J* = 8.7)	6.83 (d, *J* = 8.5)	
4′										
5′	1.57 (s)							6.84 (d, *J* = 8.7)	6.83 (d, *J* = 8.7)	
6′	1.57 (s)							7.38 (d, *J* = 8.7)	7.38 (d, *J* = 8.5)	
1′’								1.75 (m)		
2′’								2.09 (m)		
3′’	2.63 (s)							5.08 (t, *J* = 7.1)		
4′’										
5′’	1.57 (s)							1.59 (s)		
6′’	1.57 (s)							1.64 (s)		
OH-11			4.85 (s)							
OH-12			5.81 (s)	7.24 (s)						
OMe-3							3.85 (s)			
OMe-5							3.85 (s)			

^a^ Measured in CD_3_COCD_3_ at 400 MHz.

**Table 2:** ^13^C NMR Data for Compounds **1**–**10** (*δ* in ppm, 125 MHz for ^13^C NMR in CDCl_3_).

No	1 ^a^	2	3	4	5	6	7	8	9	10
1		44.7	35.3	34.3	37.8	37.5	132.2			50.8
2	160.9	33.7	33.6	27.6	18.5	18.1	104.9	79.8	75.6	50.2
3	95.2	121.6	219.7	78.2	36.6	36.9	146.9	129.8	33.0	81.4
4	124.0	133.3	47.7	39.0	47.3	47.1	132.9	122.7	22.2	35.3
5	119.8	27.7	49.2	51.1	44.5	48.9	146.9	116.1	118.5	52.6
6	121.9	30.5	74.3	17.2	21.6	26.1	104.9	133.6	133.0	42.6
7	122.02	53.4	68.3	26.8	29.9	38.6	32.3	113.3	110.5	60.9
8	111.2	26.1	125.3	145.7	135.1	154.2	30.4	144.4	145.3	74.8
9	132.1	32.0	130.4	146.0	148.6	59.7	60.8	138.5	140.2	34.2
10		40.9	38.4	38.1	37.0	39.7		121.1	121.1	32.1
11		74.9	140.0	183.3	124.4	20.9	171.1			43.1
12		72.1	142.4	150.6	122.8	35.4				12.4
13		23.7	132.9	123.9	142.6	211.2				30.2
14		18.0	119.7	187.3	126.0	125.1				29.5
15		23.1	27.2	24.0	70.1	49.5				27.3
16		171.2	22.5	20.0	24.8	23.8				
17		20.9	22.8	19.8	184.5	29.1				
18			32.0	28.2	16.2	183.8				
19			19.0	15.7	25.0	16.5				
20			20.1	19.9		14.6				
1′								133.8	134.0	
2′	86.2							127.8	127.9	
3′	48.7							115.5	115.5	
4′	184.0							154.7	154.0	
5′	26.9							115.5	115.5	
6′	26.9							127.8	127.9	
1′’								41.7		
2′’								22.8		
3”								123.8		
4”								131.9		
5”								17.6		
6”								25.6		
Me-2									26.9(×2)	
Me-11							20.9			
OMe-3							56.2			
OMe-5							56.2			

^a^ Measured in CD_3_COCD_3_ at 100 MHz.

**Table 3:** Inhibitory Effects of the 9 isolates (**2**–**10**) from *Amycolatopsis taiwanensis* on LPS-activated NO productions in RAW 264.7 macrophages.

Compounds	IC_50_ (μM) ^a^)	
	NO	Cell Viability (% Control)
Amycolataiwanensin B (**2**)	34.23 ± 1.97 *	99.62 ± 5.53
Amycolataiwanensin C (**3**)	17.52 ± 1.85 *	95.53 ± 3.39
Amycolataiwanensin D (**4**)	24.75 ± 1.63 *	91.12 ± 4.22
Amycolataiwanensin E (**5**)	12.31 ± 4.97 **	82.22 ± 3.21
Amycolataiwanensin F (**6**)	24.83 ± 12.91 **	76.35 ± 5.48
Amycolataiwanensin G (**7**)	12.78 ± 4.82 *	56.27 ± 3.91
Amycolataiwanensin H (**8**)	17.81 ± 1.132 **	95.91 ± 1.32
Amycolataiwanensin I (**9**)	13.32 ± 0.51 *	98.03 ± 1.53
Amycolataiwanensin J (**10**)	>100	89.50 ± 2.15
Quercetin ^b^	35.94 ± 2.34 *	94.11 ± 1.32

^a^ The IC_50_ values were calculated from the slope of the dose-response curves (SigmaPlot). Values are expressed as mean ± SEM (*n* = 4) of 3 independent experiments. * *p* < 0.05, ** *p* < 0.01 compared with the control. ^b^ Quercetin was used as a positive control.

## Data Availability

The data presented in this study are available in article and Supplementary Material.

## Supplementary Materials

The following are available online at https://www.mdpi.com/article/10.3390/molecules26195765/s1. Figure S1: ^1^H NMR spectrum of **1**; Figure S2. ^13^C NMR spectrum of **1**; Figure S3: ^1^H-^1^H COSY spectrum of **1**; Figure S4: HMBC spectrum of **1;** Figure S5: NOESY spectrum of **1**; Figure S6: HSQC spectrum of **1**; Figure S7: EI-MS spectrum of **1**; Figure S8: ^1^H NMR spectrum of **2**; Figure S9: ^13^C NMR spectrum of **2**; Figure S10: ^1^H-^1^H COSY spectrum of **2**; Figure S11: HMBC spectrum of **2**; Figure S12: NOESY spectrum of **2**; Figure S13: HSQC spectrum of **2**; Figure S14: EI-MS spectrum of **2**; Figure S15: ^1^H NMR spectrum of **3**; Figure S16: ^13^C NMR spectrum of **3**; Figure S17: ^1^H-^1^H COSY spectrum of **3**; Figure S18: HMBC spectrum of **3**; Figure S19: NOESY spectrum of **3**; Figure S20: HSQC spectrum of **3**; Figure S21: EI-MS spectrum of **3**; Figure S22: ^1^H NMR spectrum of **4**; Figure S23: ^13^C NMR spectrum of **4**; Figure S24: ^1^H-^1^H COSY spectrum of **4**; Figure S25: HMBC spectrum of **4**; Figure S26: NOESY spectrum of **4**; Figure S27: HSQC spectrum of **4**; Figure S28: EI-MS spectrum of **4**; Figure S29: ^1^H NMR spectrum of **5**; Figure S30: ^13^C NMR spectrum of **5**; Figure S31: ^1^H-^1^H COSY spectrum of **5**; Figure S32: HMBC spectrum of **5**; Figure S33: NOESY spectrum of **5**; Figure S34: HSQC spectrum of **5**; Figure S35: EI-MS spectrum of **5**; Figure S36: ^1^H NMR spectrum of **6**; Figure S37: ^13^C NMR/DEPT spectra of **6**; Figure S38: ^1^H-^1^H COSY spectrum of **6**; Figure S39: HMBC spectrum of **6**; Figure S40: NOESY spectrum of **6**; Figure S41: HSQC spectrum of **6**; Figure S42: EI-MS spectrum of **6**; Figure S43: ^1^H NMR spectrum of **7**; Figure S44: ^13^C NMR/DEPT spectra of **7**; Figure S45: ^1^H-^1^H COSY spectrum of **7**; Figure S46: HMBC spectrum of **7**; Figure S47: NOESY spectrum of **7**; Figure S48: HSQC spectrum of **7**; Figure S49: EI-MS spectrum of **7**; Figure S50: ^1^H NMR spectrum of **8**; Figure S51: ^13^C NMR/DEPT spectra of **8**; Figure S52: ^1^H-^1^H COSY spectrum of **8**; Figure S53: HMBC spectrum of **8**; Figure S54: NOESY spectrum of **8**; Figure S55: HSQC spectrum of **8**; Figure S56: EI-MS spectrum of **8**; Figure S57: ^1^H NMR spectrum of **9**; Figure S58: ^13^C NMR/DEPT spectra of **9**; Figure S59: ^1^H-^1^H COSY spectrum of **9**; Figure S60: HMBC spectrum of **9**; Figure S61: NOESY spectrum of **9**; Figure S62: HSQC spectrum of **9**; Figure S63: EI-MS spectrum of **9**; Figure S64: ^1^H NMR spectrum of **10**; Figure S65: ^13^C NMR/DEPT spectra of **10**; Figure S66: ^1^H-^1^H COSY spectrum of **10**; Figure S67: HMBC spectrum of **10**; Figure S68: NOESY spectrum of **10**; Figure S69: HSQC spectrum of **10**; Figure S70: EI-MS spectrum of **10**.
