# Supplementary material for: Secondary Metabolites with Anti-Inflammatory Activities from One Actinobacteria Amycolatopsis taiwanensis
Source: Molecules. 2021 Sep 23;26(19):5765. doi: 10.3390/molecules26195765 (PMC8510368; doi:10.3390/molecules26195765)
Supplement: Supplementary file 1 [file molecules-26-05765-s001.zip › molecules-1359869-supplementary.pdf]

molecules

Supplementary File

Secondary Metabolites with Anti-Inflammatory Activities from one Actinobacteria *Amycolatopsis taiwanensis*

Yung-Shun Su [1,2], Ming-Der Wu [3], Jih-Jung Chen [4,5,*], Ming-Jen Cheng [3,*], Yueh-Hsiung Kuo [6,7,8], Chee-Yin Chai [1,9] and Aij-Lie Kwan [1,10,11,*]

1 Graduate Institute of Medicine, College of Medicine, Kaohsiung Medical University (KMU), Kaohsiung 807, Taiwan; mariussu@gmail.com (Y.-S.S.); cychai@kmu.edu.tw (C.-Y.C.); aijliekw@kmu.edu.tw (A.-L.K.)
2 Department of Dermatology, Kaohsiung Medical University Chung-Ho Memorial Hospital, Kaohsiung 807, Taiwan
3 Bioresource Collection and Research Center (BCRC), Food Industry Research and Development Institute (FIRDI), Hsinchu 300, Taiwan; wmd@firdi.org.tw
4 Department of Pharmacy, School of Pharmaceutical Sciences, National Yang Ming Chiao Tung University (NYCU), Taipei 112, Taiwan
5 Department of Medical Research, China Medical University Hospital, Taichung 404, Taiwan
6 Department of Chemistry, National Taiwan University, Taipei 106, Taiwan; yhkuo800@gmail.com
7 Department of Biotechnology, Asia University, Taichung 413, Taiwan
8 Department of Chinese Pharmaceutical Sciences and Chinese Medicine Resources, College of Pharmacy, China Medical University, Taichung 404, Taiwan
9 Department of Pathology, Kaohsiung Medical University Chung-Ho Memorial Hospital, Kaohsiung 807, Taiwan
10 Ph.D. Program in Environmental and Occupational Medicine, College of Medicine, Kaohsiung Medical University and National Health Research Institutes, Kaohsiung 807, Taiwan
11 Department of Neurosurgery, Kaohsiung Medical University Chung-Ho Memorial Hospital, Kaohsiung 807, Taiwan
* Correspondence: jjungchen@nycu.edu.tw (J.-J.C.); chengfirdi@gmail.com (M.-J.C.)

Citation: Su, Y.-S.; Wu, M.-D.; Chen, J.-J.; Cheng, M.-J.; Kuo, Y.-H.; Chai, C.-Y.; Kwan, A.-L. Secondary Metabolites with Anti-Inflammatory Activities from one Actinobacteria *Amycolatopsis taiwanensis*. *Molecules* **2021**, *26*, 5765. https://doi.org/10.3390/molecules26195765

Academic Editor: Bruno Botta

Received: 13 August 2021
Accepted: 10 September 2021
Published: 21 September 2021

Publisher's Note: MDPI stays neutral with regard to jurisdictional claims in published maps and institutional affiliations.

Copyright: © 2021 by the authors. Licensee MDPI, Basel, Switzerland. This article is an open access article distributed under the terms and conditions of the Creative Commons Attribution (CC BY) license (http://creativecommons.org/licenses/by/4.0/).

Contents

Figure S1. ^{1}H NMR spectrum of **1** 5
Figure S2. ^{13}C NMR spectrum of **1** 5
Figure S3. ^{1}H-^{1}H COSY spectrum of **1** 6
Figure S4. HMBC spectrum of **1** 6
Figure S5. NOESY spectrum of **1** 7
Figure S6. HSQC spectrum of **1** 7
Figure S7. EI-MS spectrum of **1** 8
Figure S8. ^{1}H NMR spectrum of **2** 9
Figure S9. ^{13}C NMR spectrum of **2** 9
Figure S10. ^{1}H-^{1}H COSY spectrum of **2** 10
Figure S11. HMBC spectrum of **2** 10
Figure S12. NOESY spectrum of **2** 11
Figure S13. HSQC spectrum of **2** 11
Figure S14. EI-MS spectrum of **2** 12
Figure S15. ^{1}H NMR spectrum of **3** 13
Figure S16. ^{13}C NMR spectrum of **3** 13
Figure S17. ^{1}H-^{1}H COSY spectrum of **3** 14
Figure S18. HMBC spectrum of **3** 14
Figure S19. NOESY spectrum of **3** 15
Figure S20. HSQC spectrum of **3** 15
Figure S21. EI-MS spectrum of **3** 16
Figure S22. ^{1}H NMR spectrum of **4** 17
Figure S23. ^{13}C NMR spectrum of **4** 17
Figure S24. ^{1}H-^{1}H COSY spectrum of **4** 18
Figure S25. HMBC spectrum of **4** 18
Figure S26. NOESY spectrum of **4** 19
Figure S27. HSQC spectrum of **4** 19
Figure S28. EI-MS spectrum of **4** 20
Figure S29. ^{1}H NMR spectrum of **5** 21
Figure S30. ^{13}C NMR spectrum of **5** 21
Figure S31. ^{1}H-^{1}H COSY spectrum of **5** 22
Figure S32. HMBC spectrum of **5** 22

Figure S33. NOESY spectrum of **5** 23
Figure S34. HSQC spectrum of **5** 23
Figure S35. EI-MS spectrum of **5** 24
Figure S36. ^{1}H NMR spectrum of **6** 25
Figure S37. ^{13}C NMR/DEPT spectra of **6** 25
Figure S38. ^{1}H-^{1}H COSY spectrum of **6** 26
Figure S39. HMBC spectrum of **6** 26
Figure S40. NOESY spectrum of **6** 27
Figure S41. HSQC spectrum of **6** 27
Figure S42. EI-MS spectrum of **6** 28
Figure S43. ^{1}H NMR spectrum of **7** 29
Figure S44. ^{13}C NMR/DEPT spectra of **7** 29
Figure S45. ^{1}H-^{1}H COSY spectrum of **7** 30
Figure S46. HMBC spectrum of **7** 30
Figure S47. NOESY spectrum of **7** 31
Figure S48. HSQC spectrum of **7** 31
Figure S49. EI-MS spectrum of **7** 32
Figure S50. ^{1}H NMR spectrum of **8** 33
Figure S51. ^{13}C NMR/DEPT spectra of **8** 33
Figure S52. ^{1}H-^{1}H COSY spectrum of **8** 34
Figure S53. HMBC spectrum of **8** 34
Figure S54. NOESY spectrum of **8** 35
Figure S55. HSQC spectrum of **8** 35
Figure S56. EI-MS spectrum of **8** 36
Figure S57. ^{1}H NMR spectrum of **9** 37
Figure S58. ^{13}C NMR/DEPT spectra of **9** 37
Figure S59. ^{1}H-^{1}H COSY spectrum of **9** 38
Figure S60. HMBC spectrum of **9** 38
Figure S61. NOESY spectrum of **9** 39
Figure S62. HSQC spectrum of **9** 39
Figure S63. EI-MS spectrum of **9** 40
Figure S64. ^{1}H NMR spectrum of **10** 41
Figure S65. ^{13}C NMR/DEPT spectra of **10** 41

Figure S66. ^{1}H-^{1}H COSY spectrum of **10** 42
Figure S67. HMBC spectrum of **10** 42
Figure S68. NOESY spectrum of **10** 43
Figure S69. HSQC spectrum of **10** 43
Figure S70. EI-MS spectrum of **10** 44

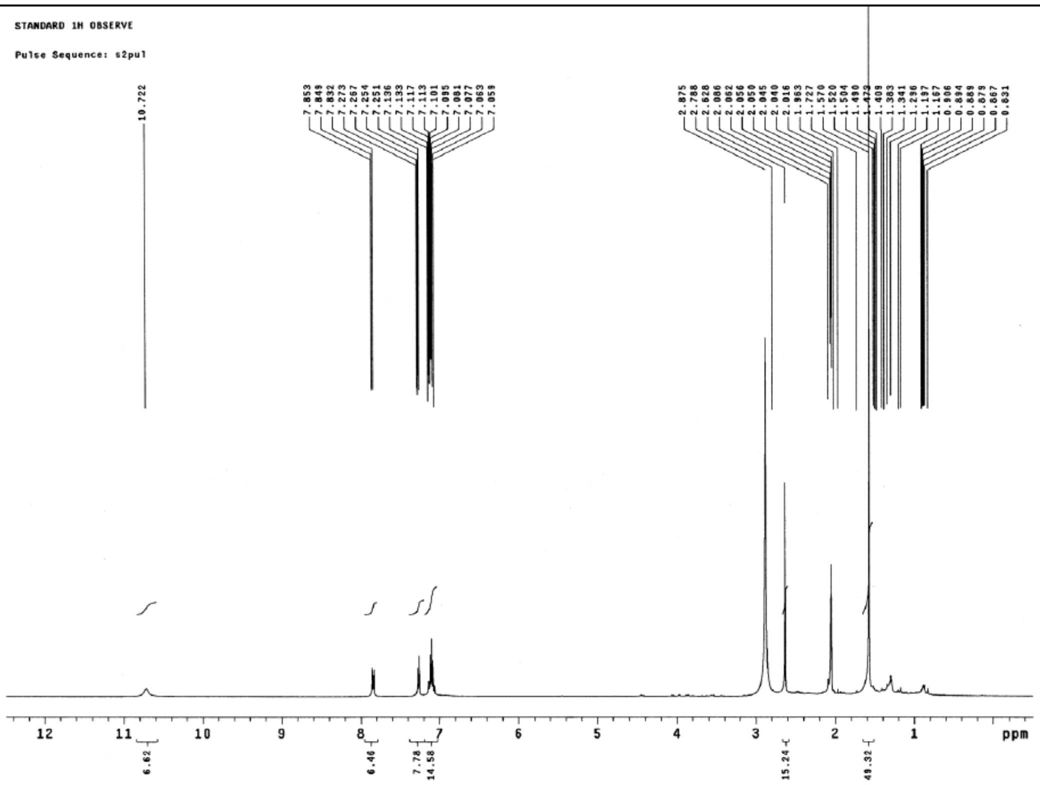

Figure S1. ^{1}H NMR spectrum of **1**

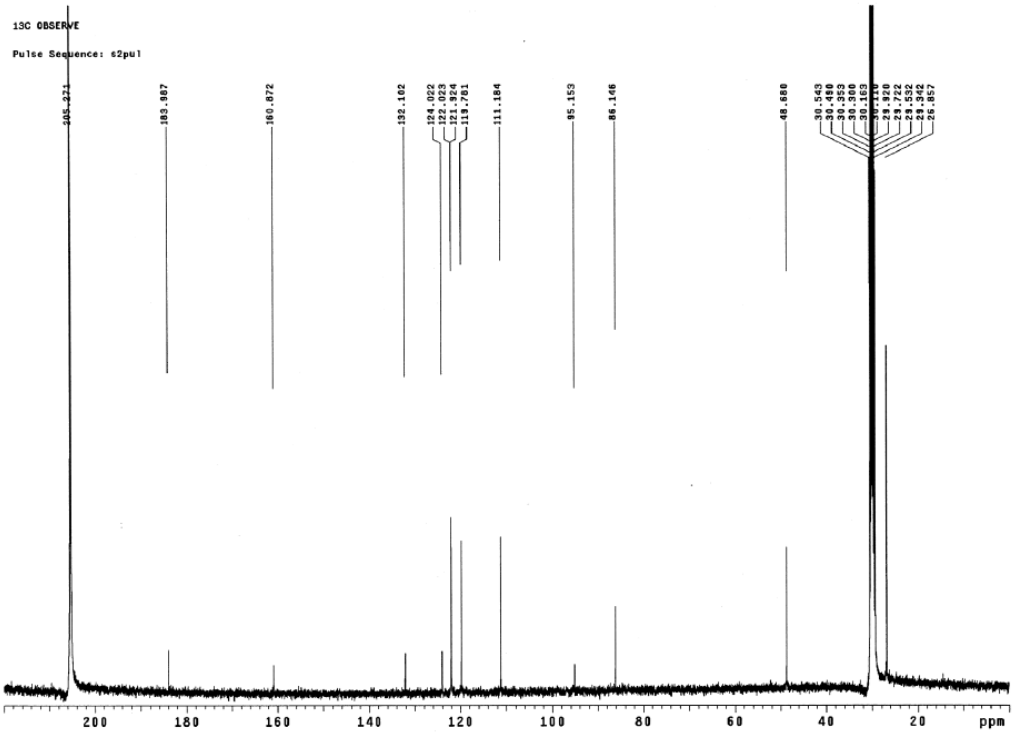

Figure S2. ^{13}C NMR spectrum of **1**

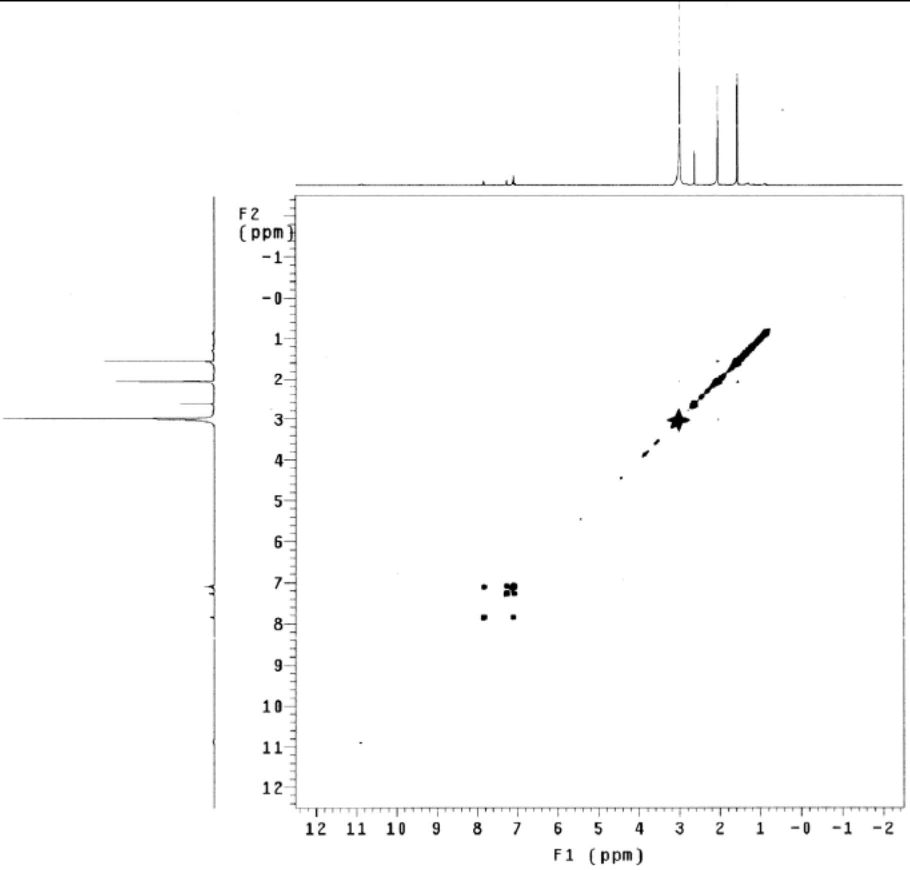

Figure S3. COSY spectrum of **1**

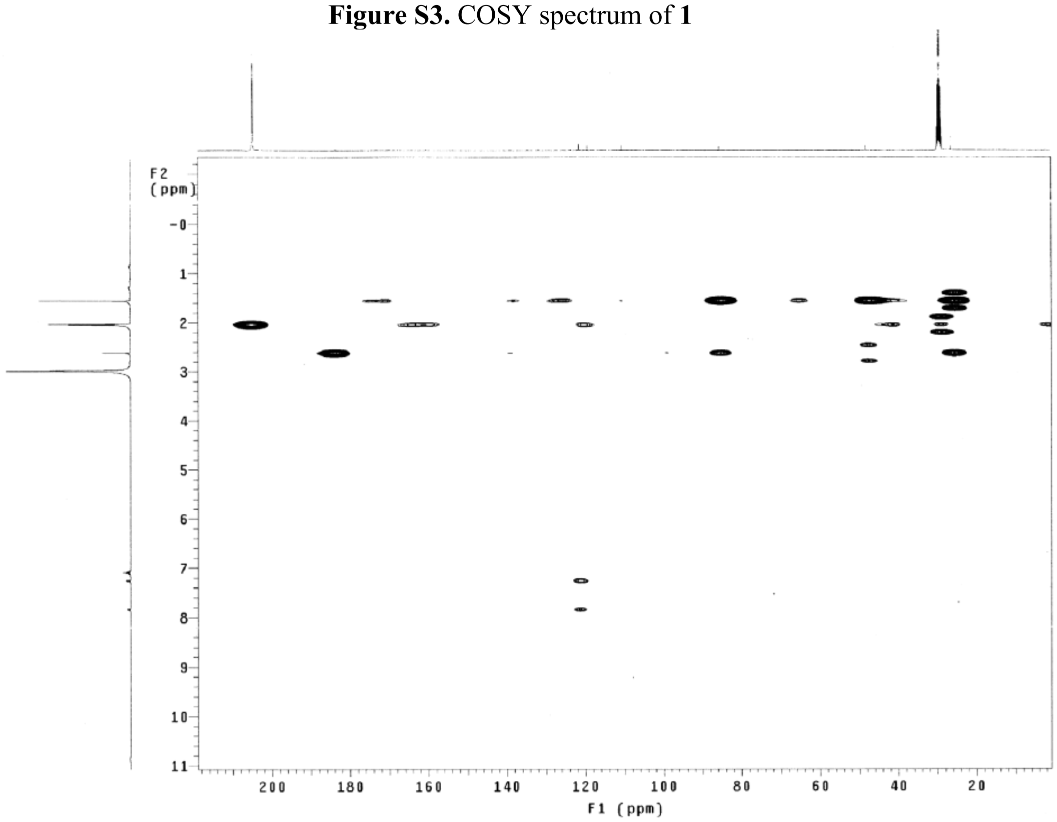

Figure S4. HMBC spectrum of **1**

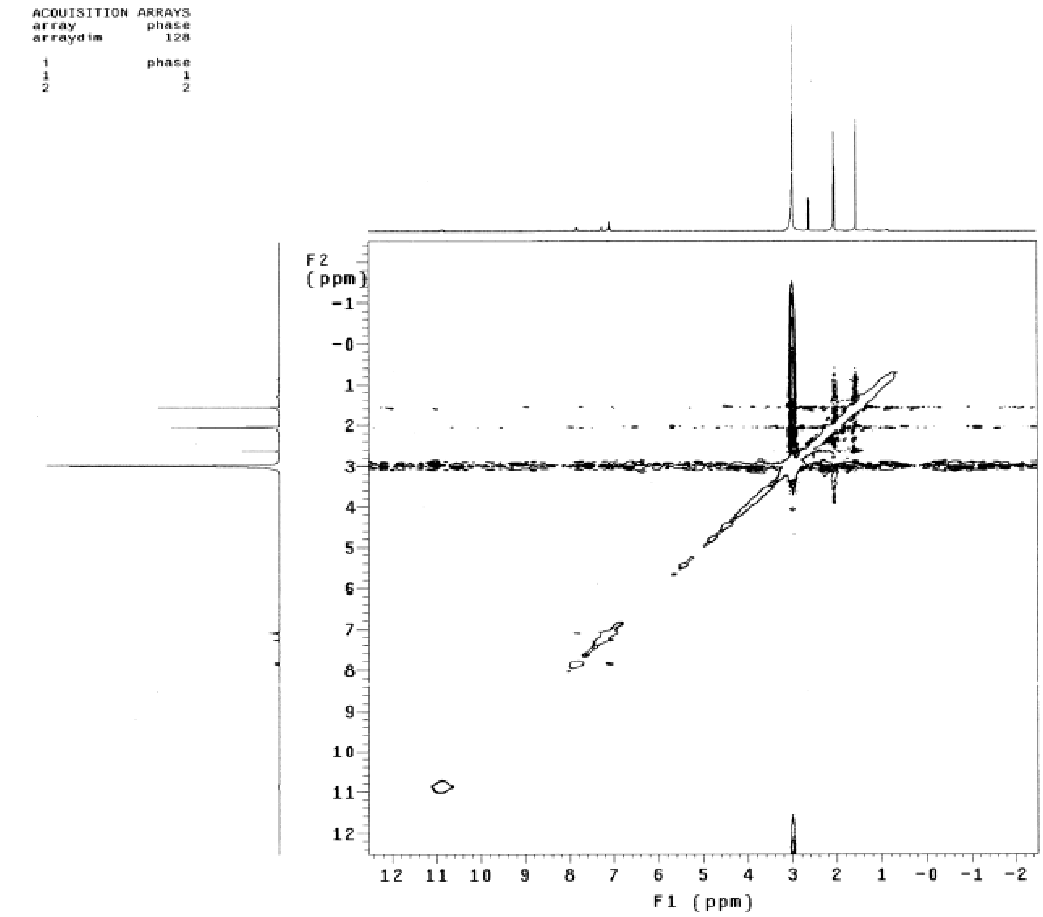

Figure S5. NOESY spectrum of **1**

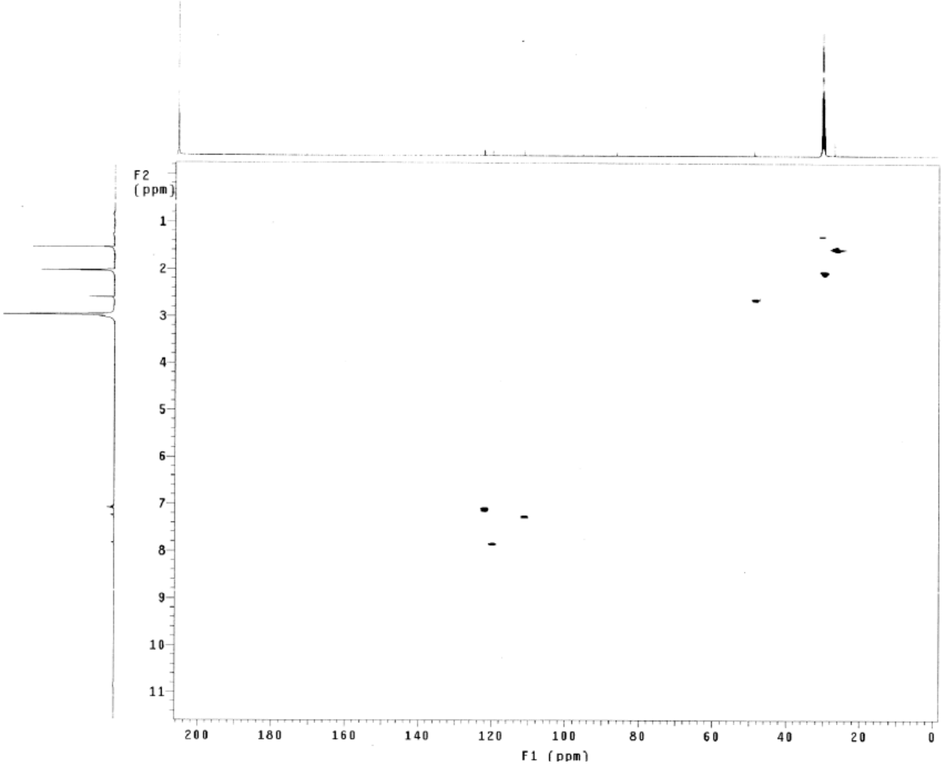

Figure S6. HSQC spectrum of **1**

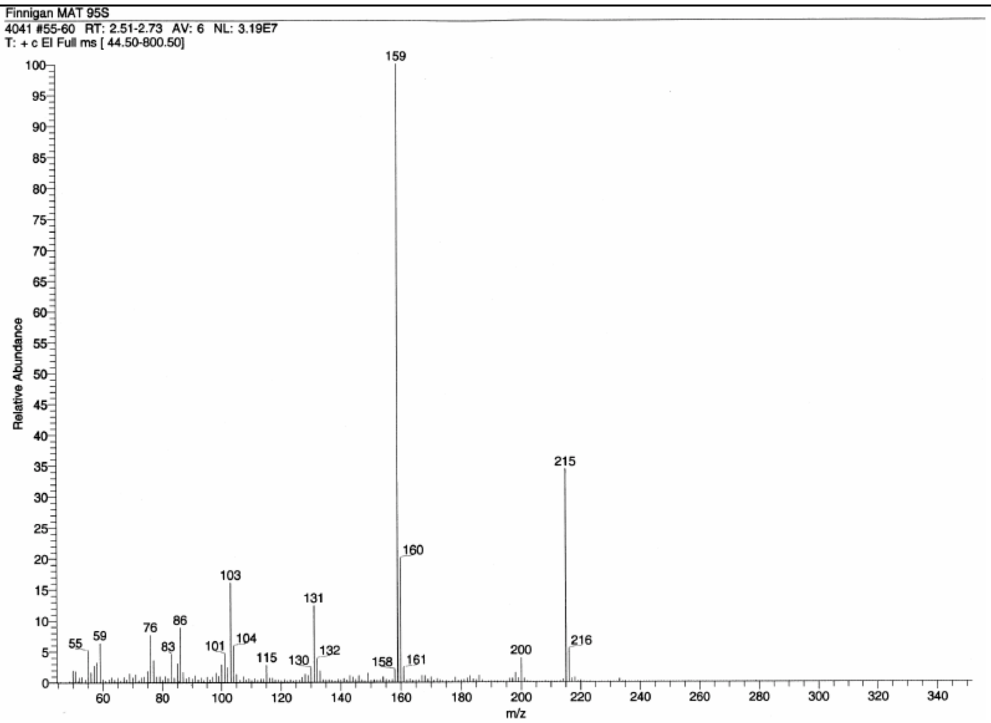

Figure S7. EIMS spectrum of **1**

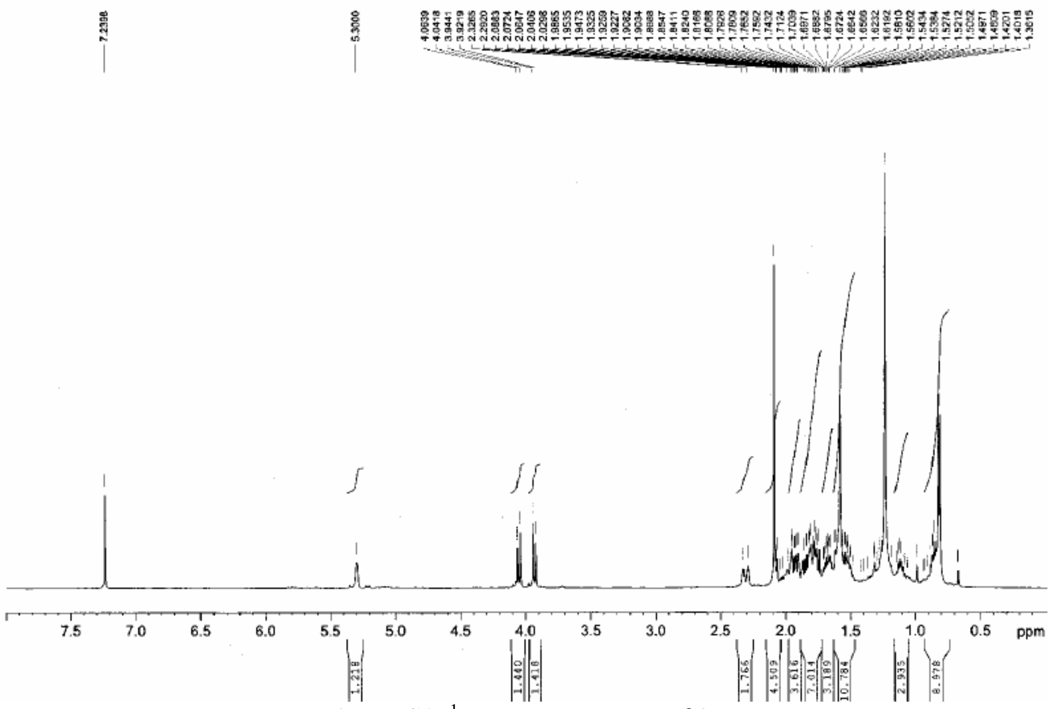

Figure S8. ^{1}H NMR spectrum of **2**

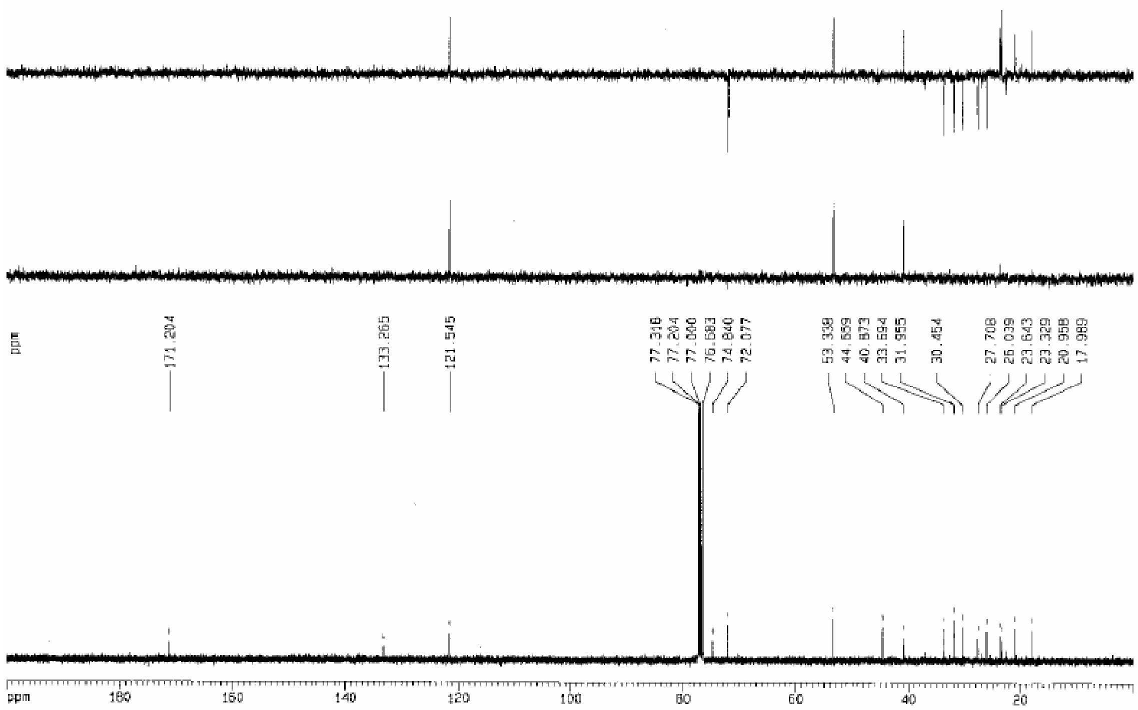

Figure S9. ^{13}C spectrum of **2**

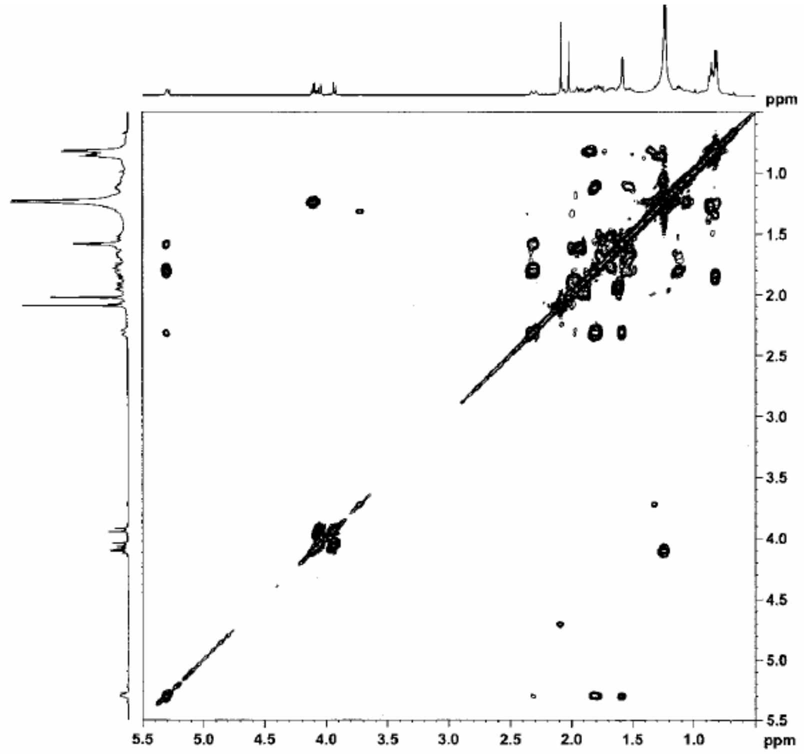

Figure S10. COSY spectrum of **2**

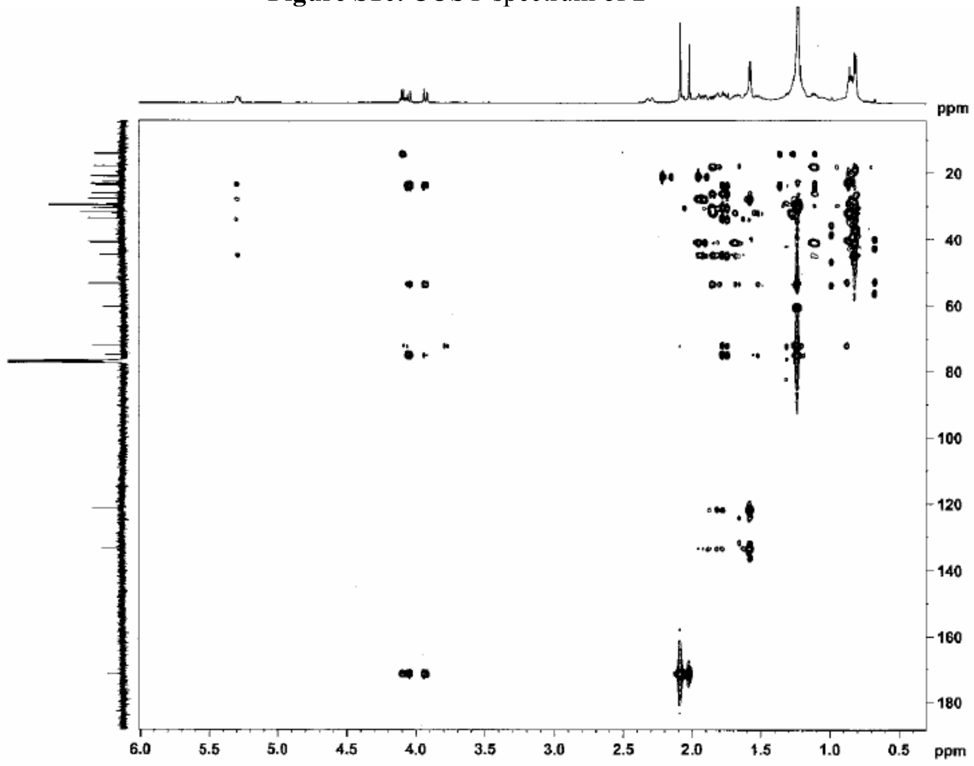

Figure S11. HMBC spectrum of **2**

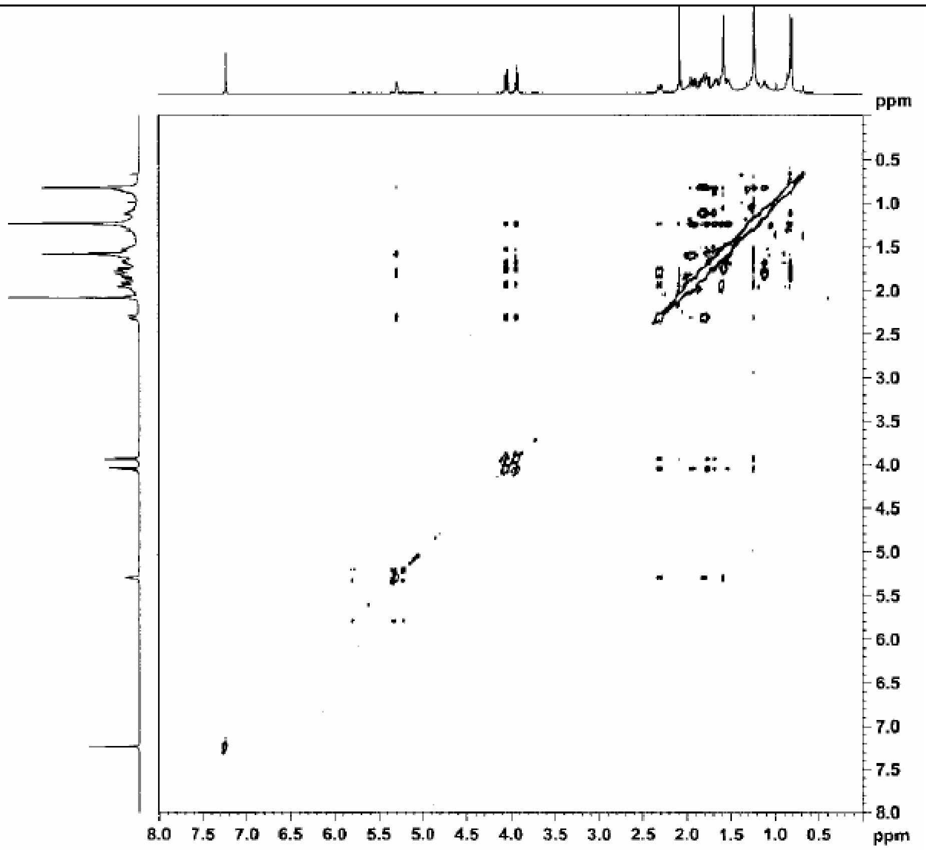

Figure S12. NOESY spectrum of **2**

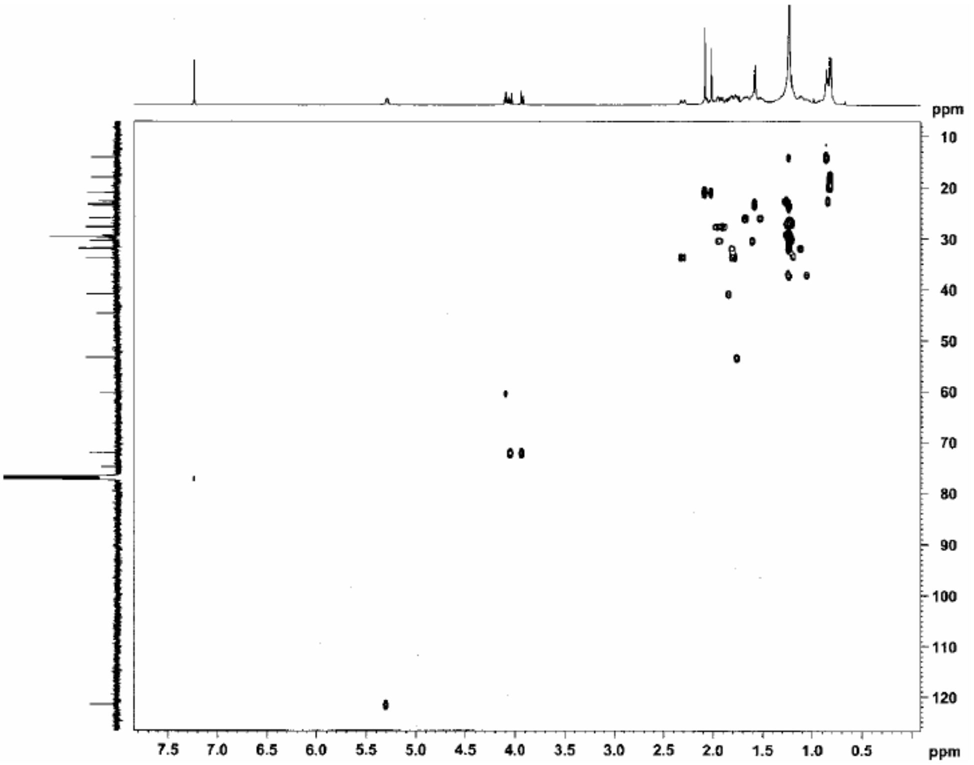

Figure S13. HSQC spectrum of **2**

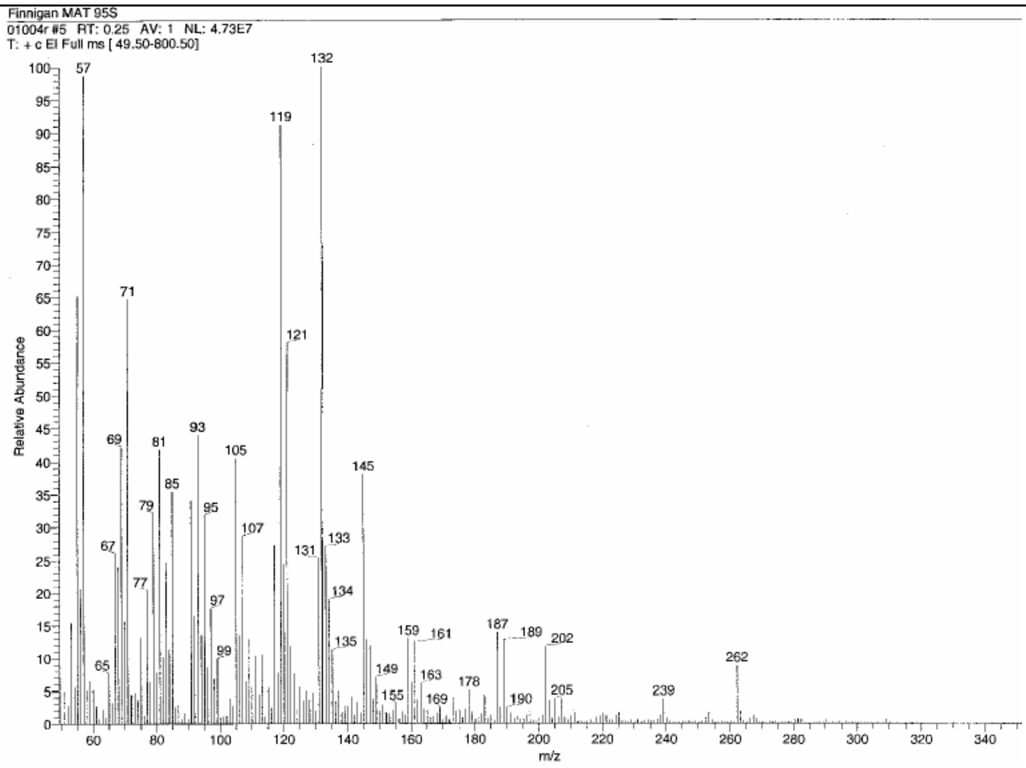

Figure S14. EI-MS spectrum of **2**

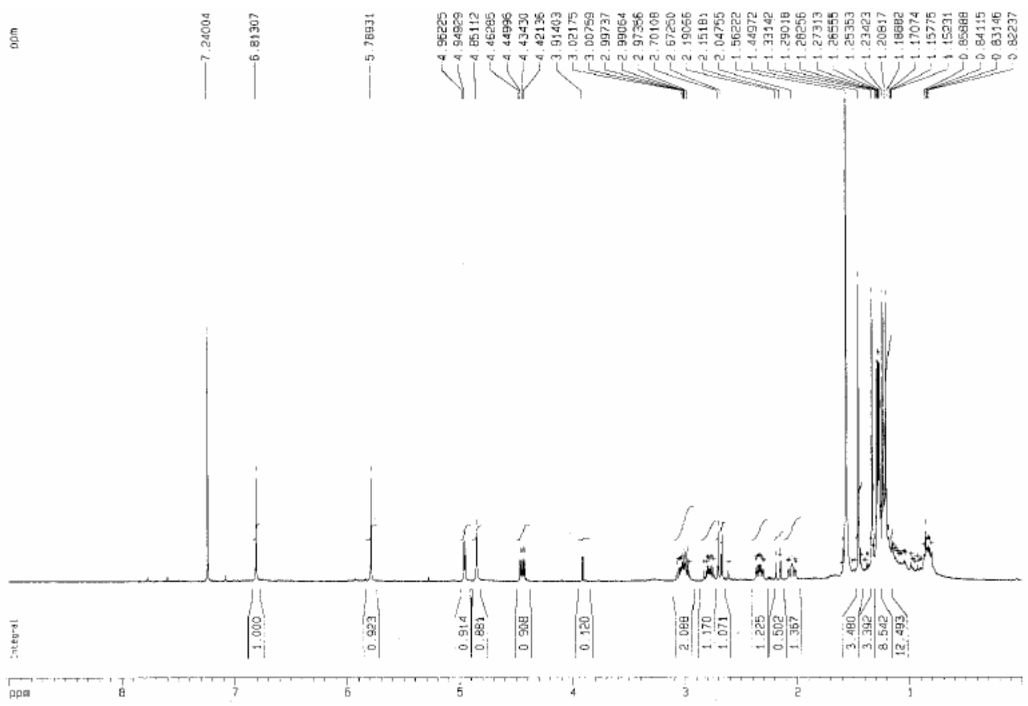

Figure S15. ^{1}H NMR spectrum of **3**

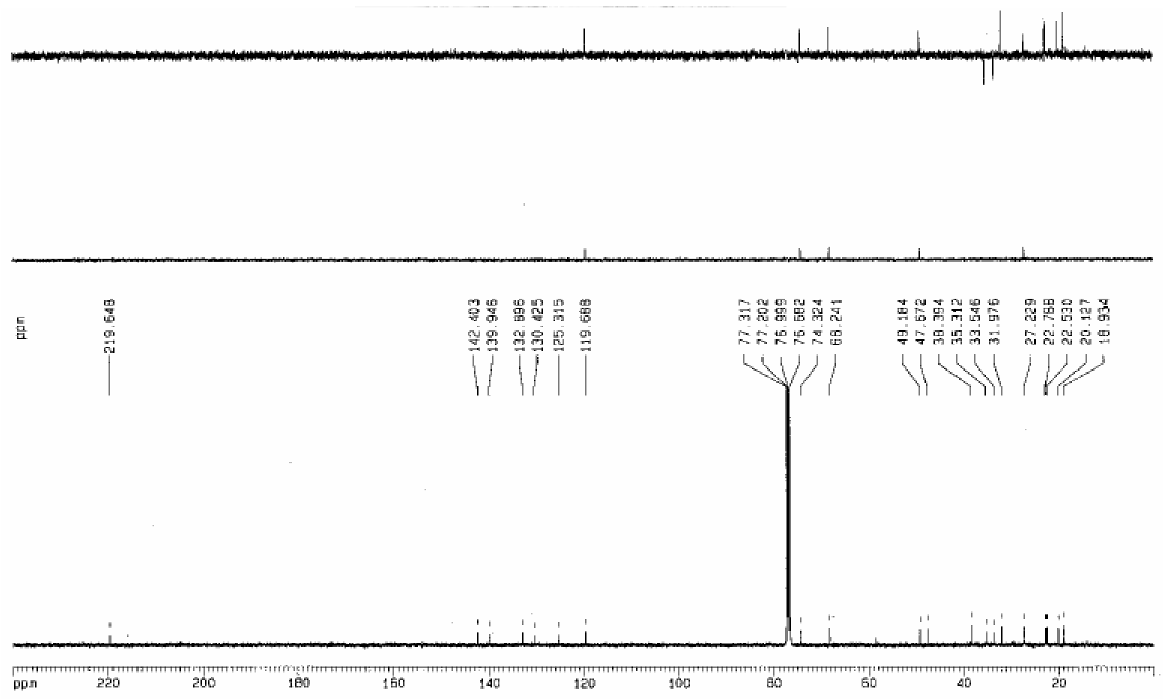

Figure S16. ^{13}C NMR spectrum of **3**

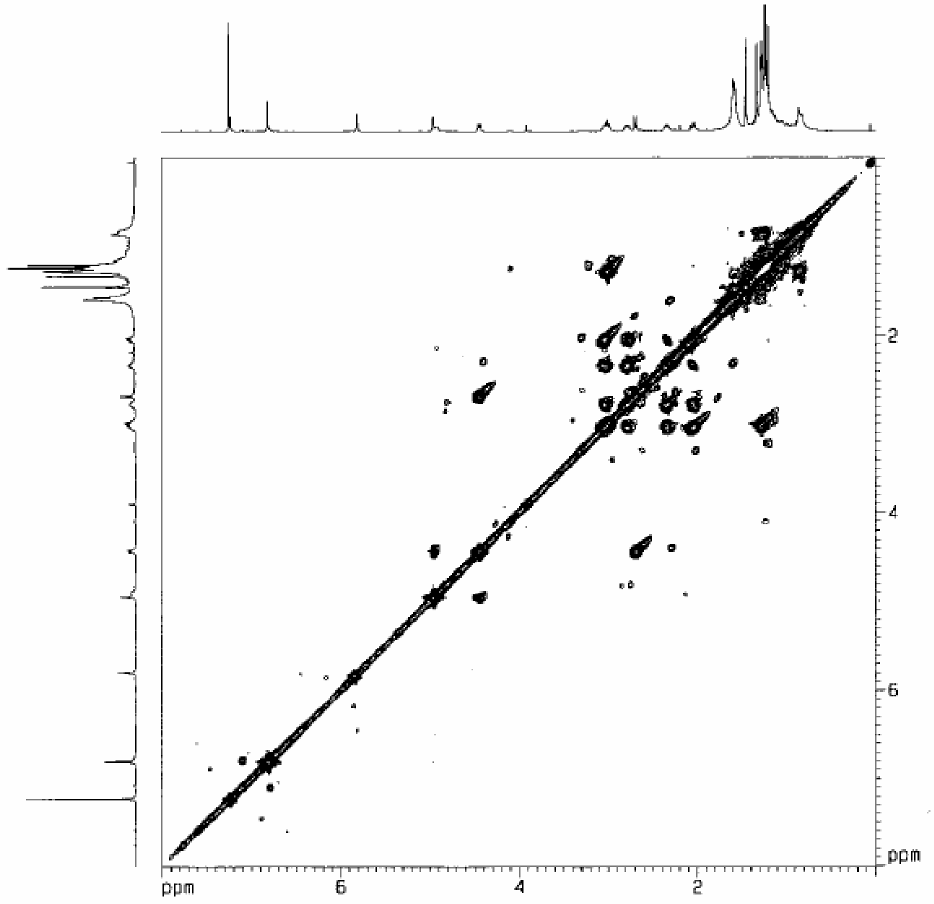

Figure S17. COSY spectrum of **3**

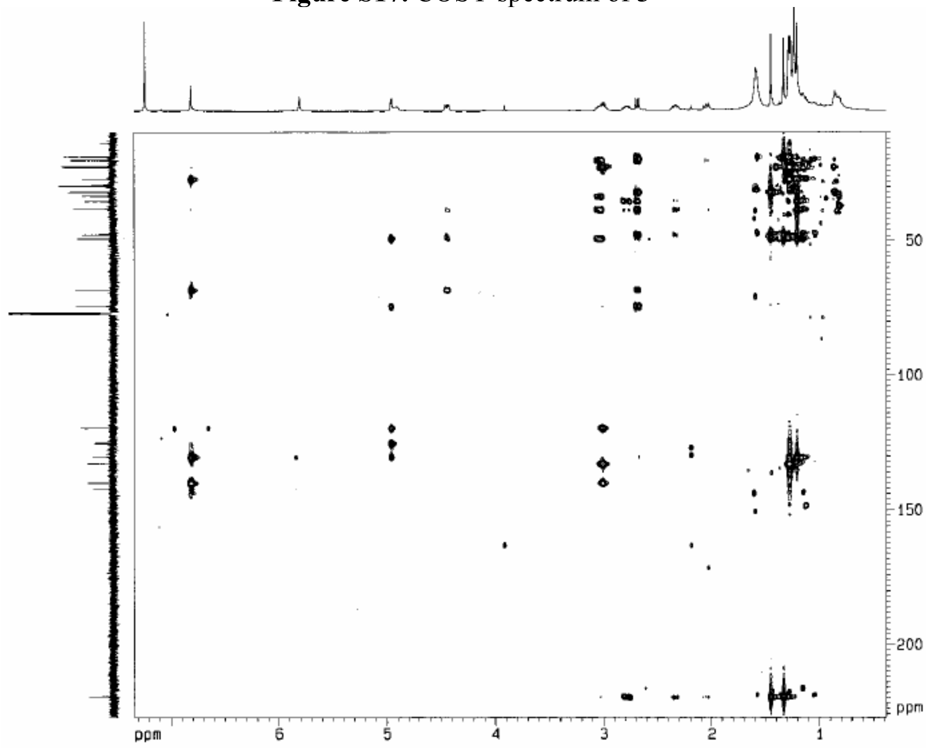

Figure S18. HMBC spectrum of **3**

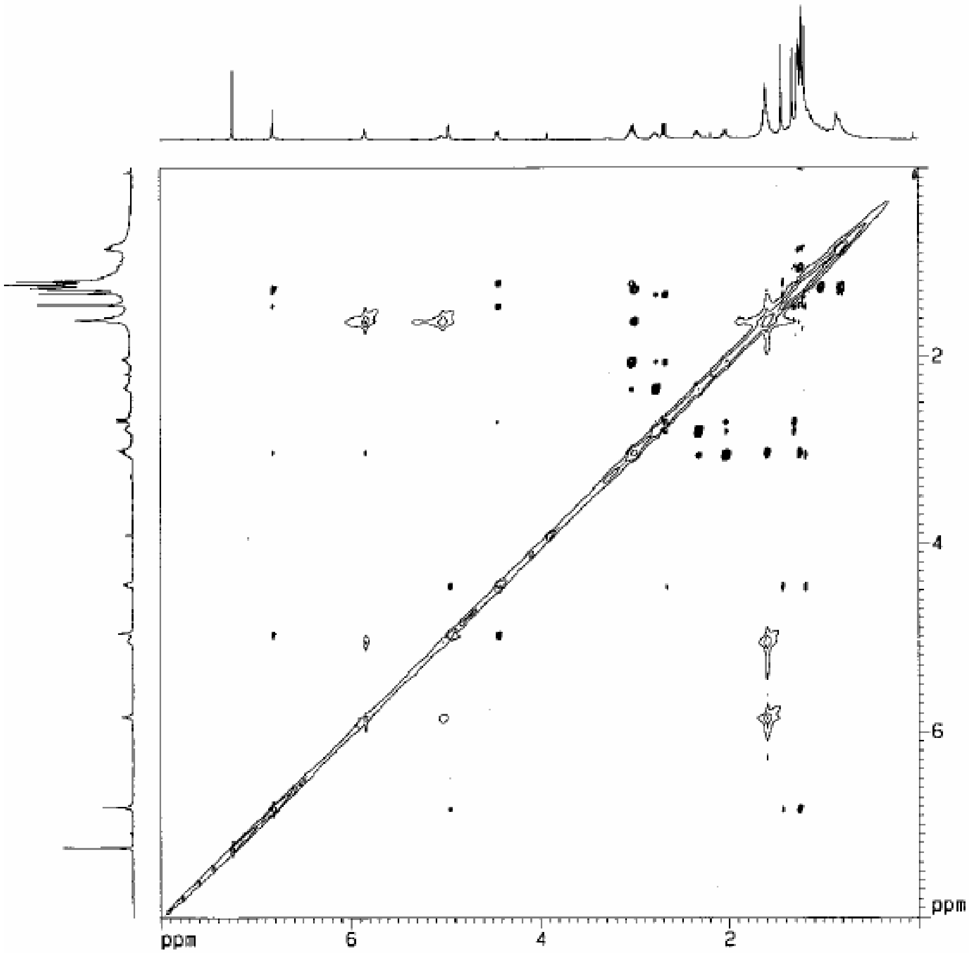

Figure S19. NOESY spectrum of **3**

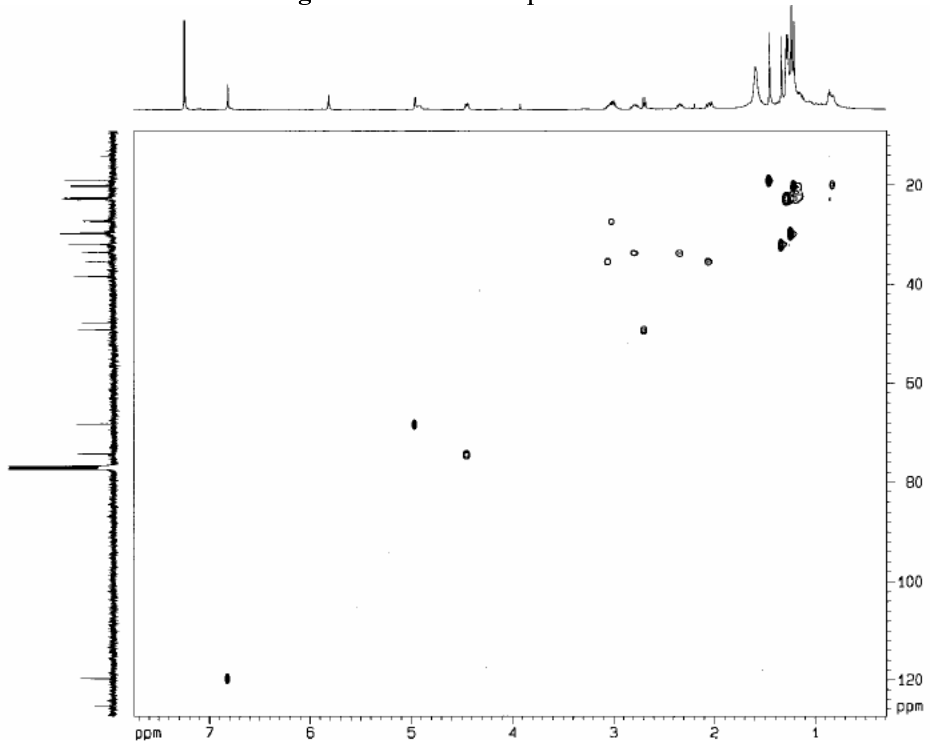

Figure S20. HSQC spectrum of **3**

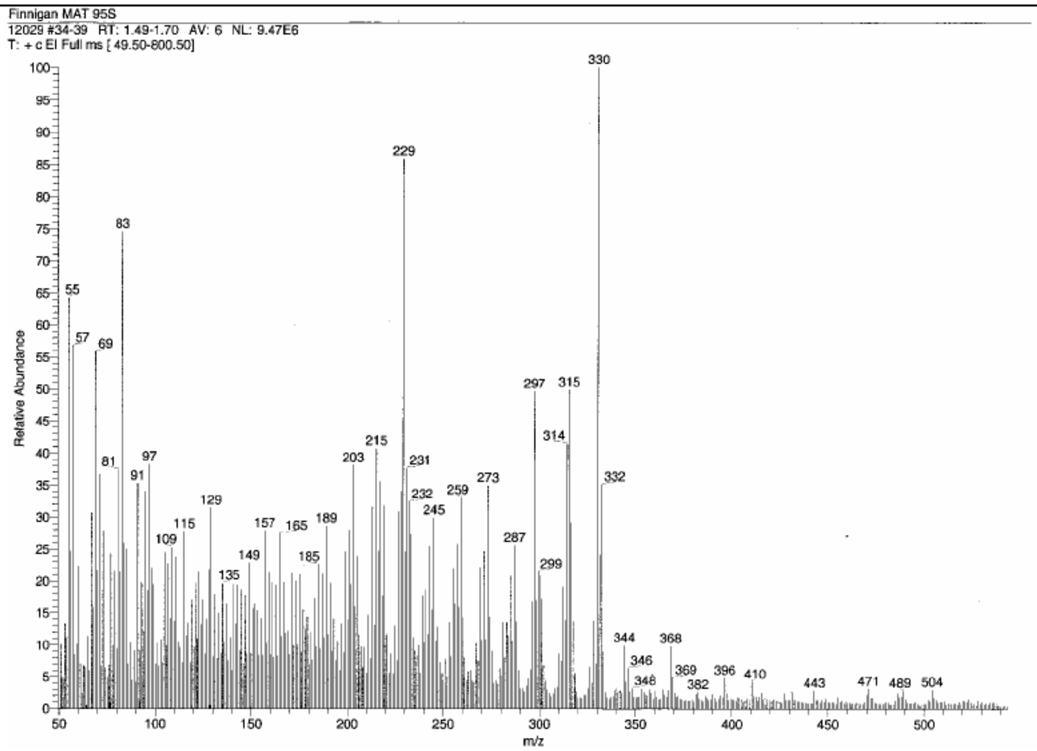

Figure S21. EI-MS spectrum of **3**

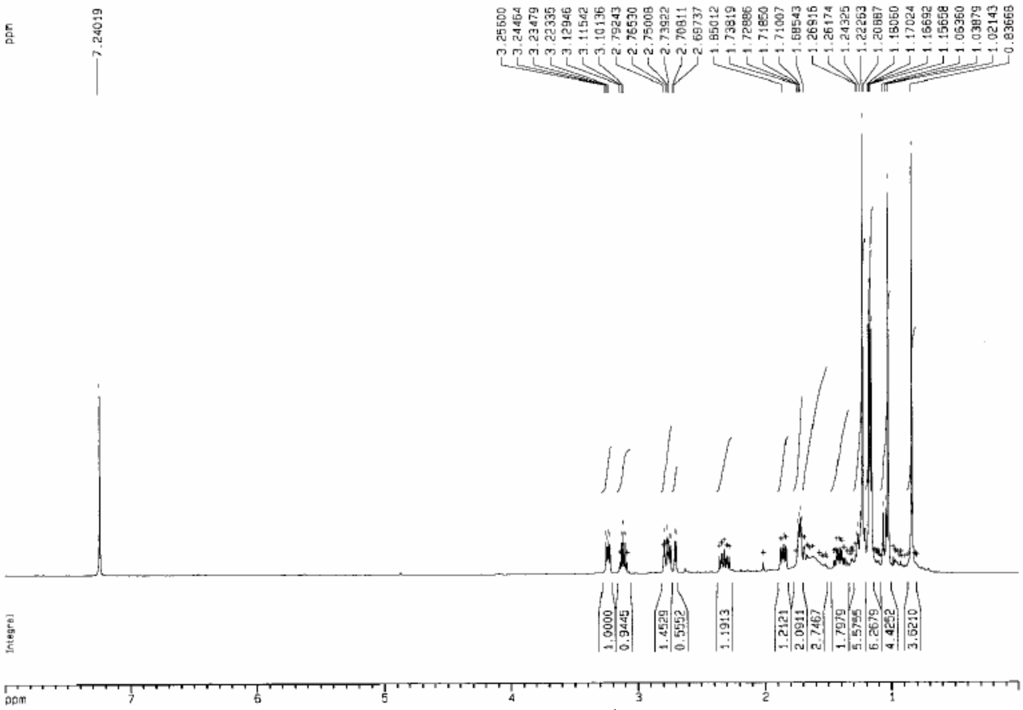

Figure S22. ^{1}H NMR spectrum of **4**

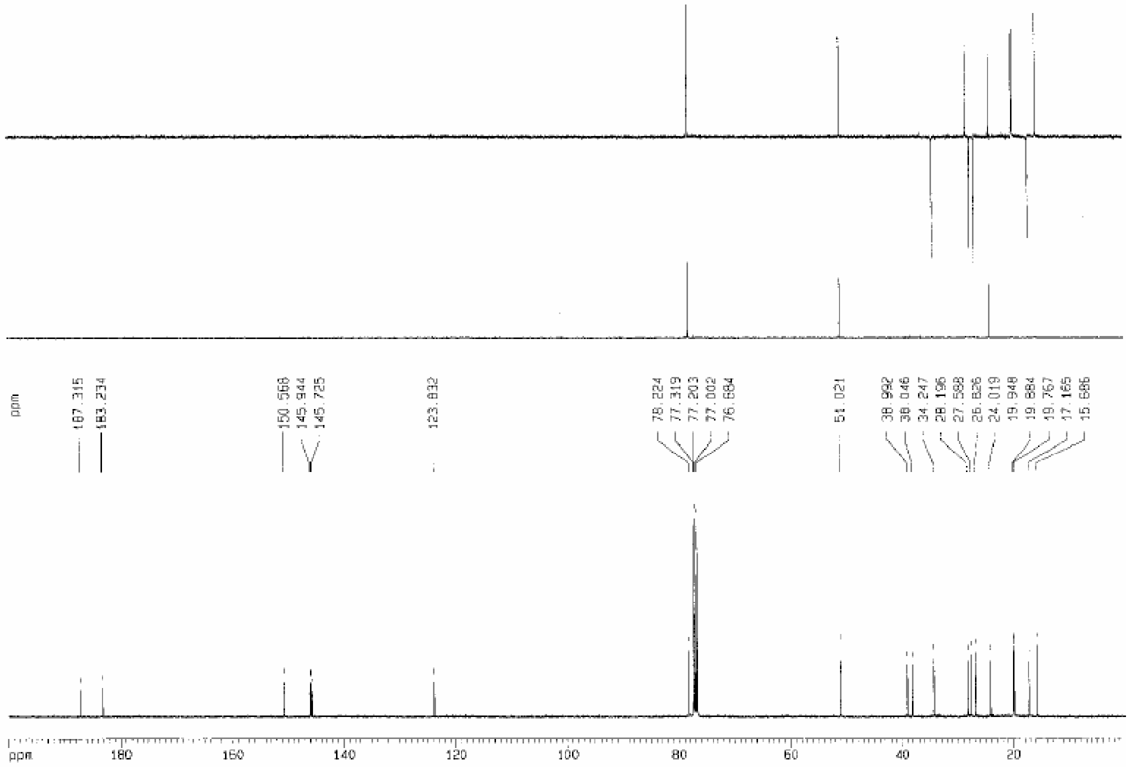

Figure S23. ^{13}C spectrum of **4**

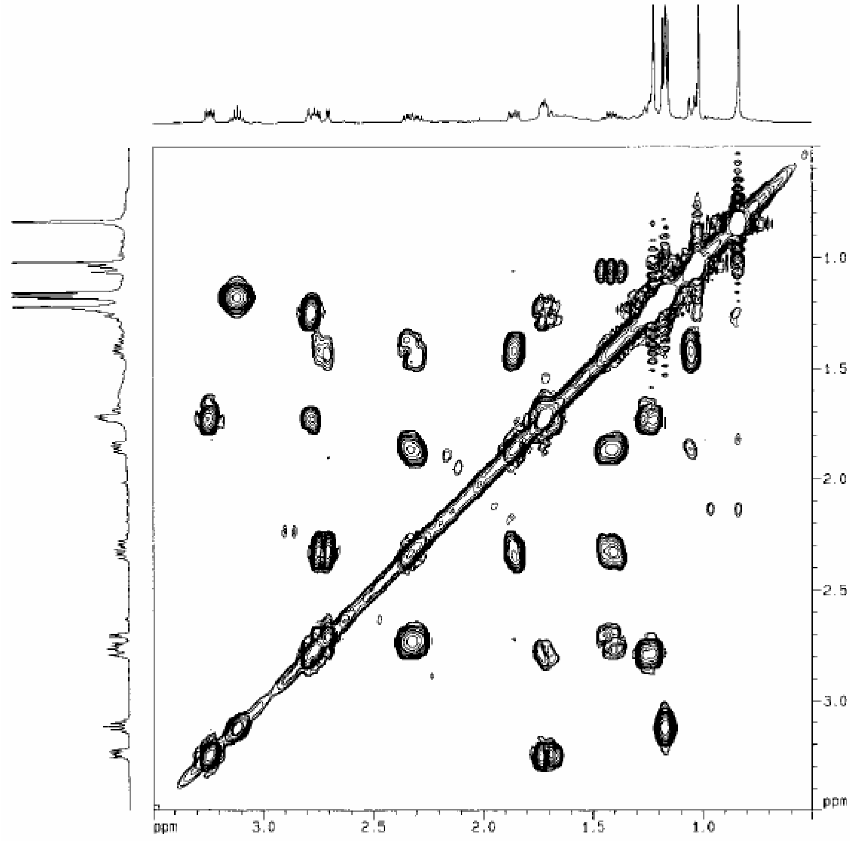

Figure S24. COSY spectrum of **4**

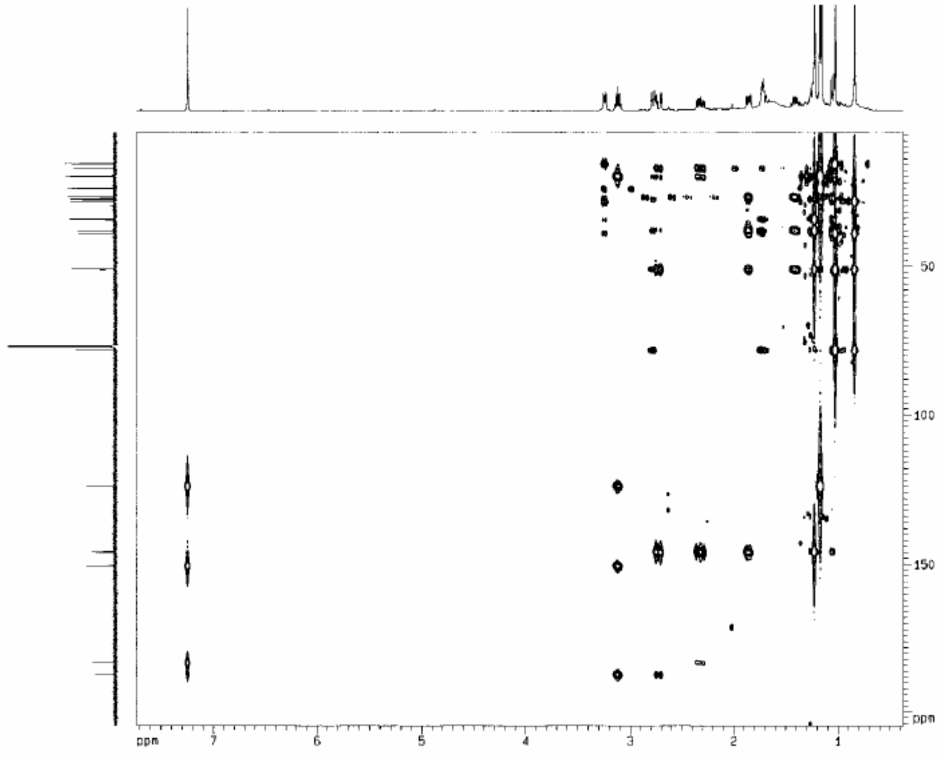

Figure S25. HMBC spectrum of **4**

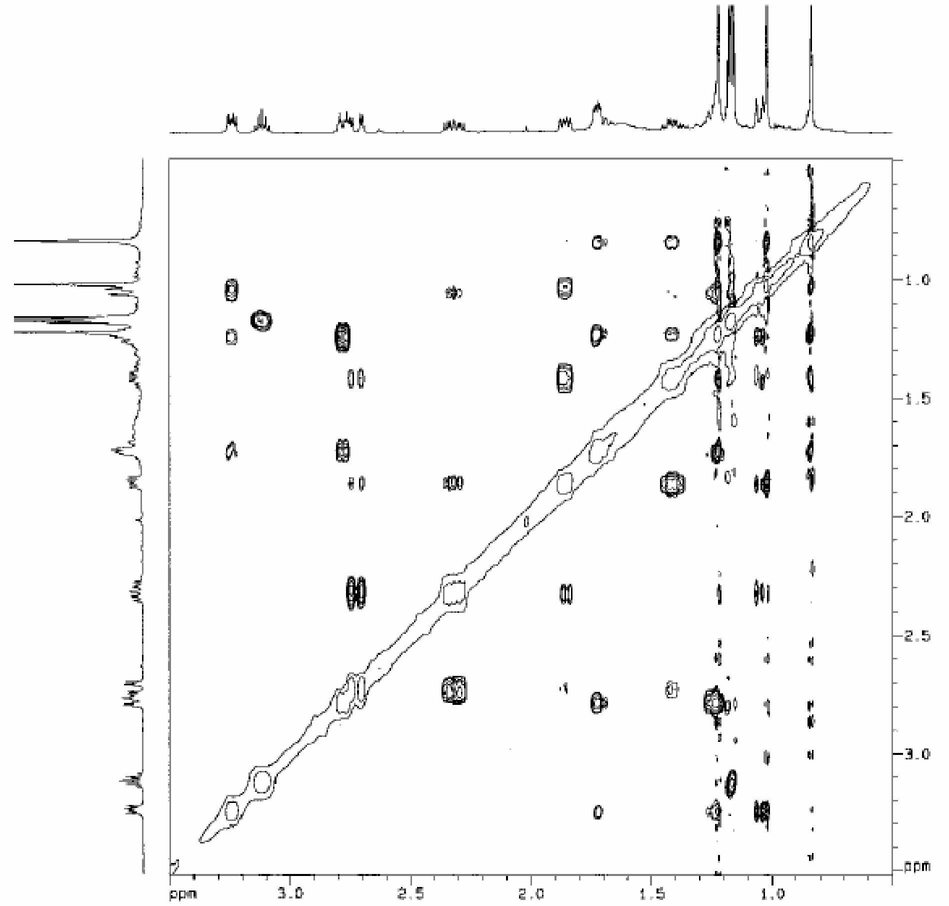

Figure S26. NOESY spectrum of **4**

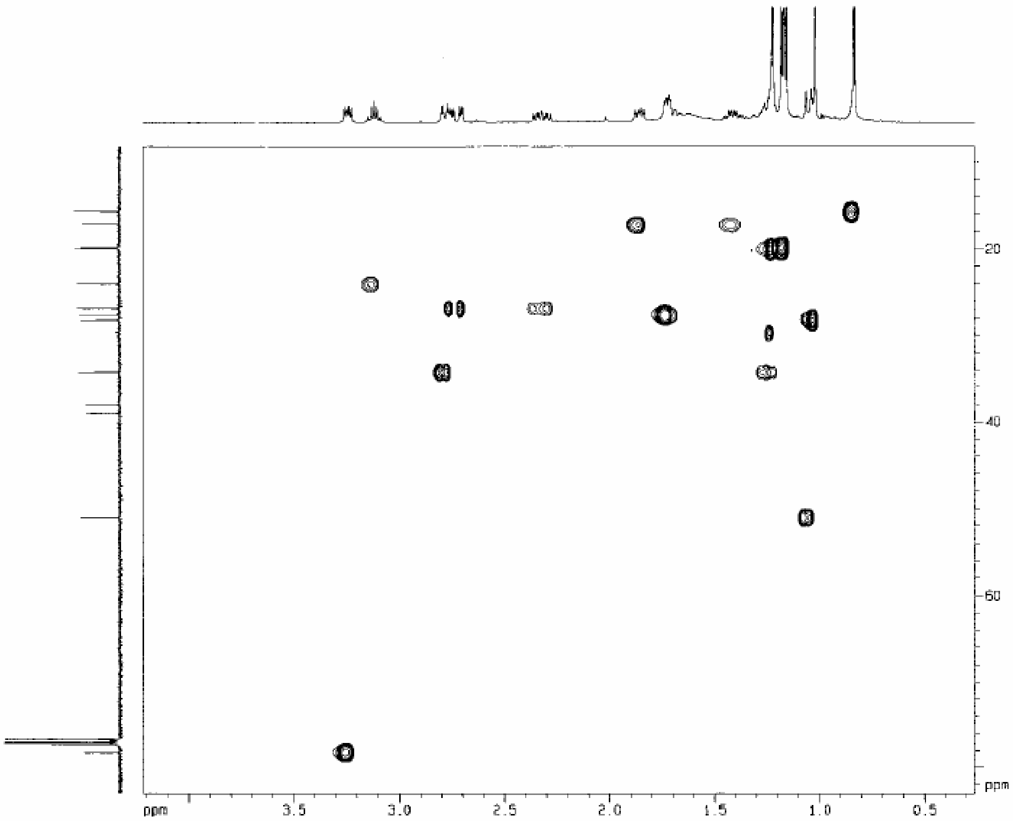

Figure S27. HSQC spectrum of **4**

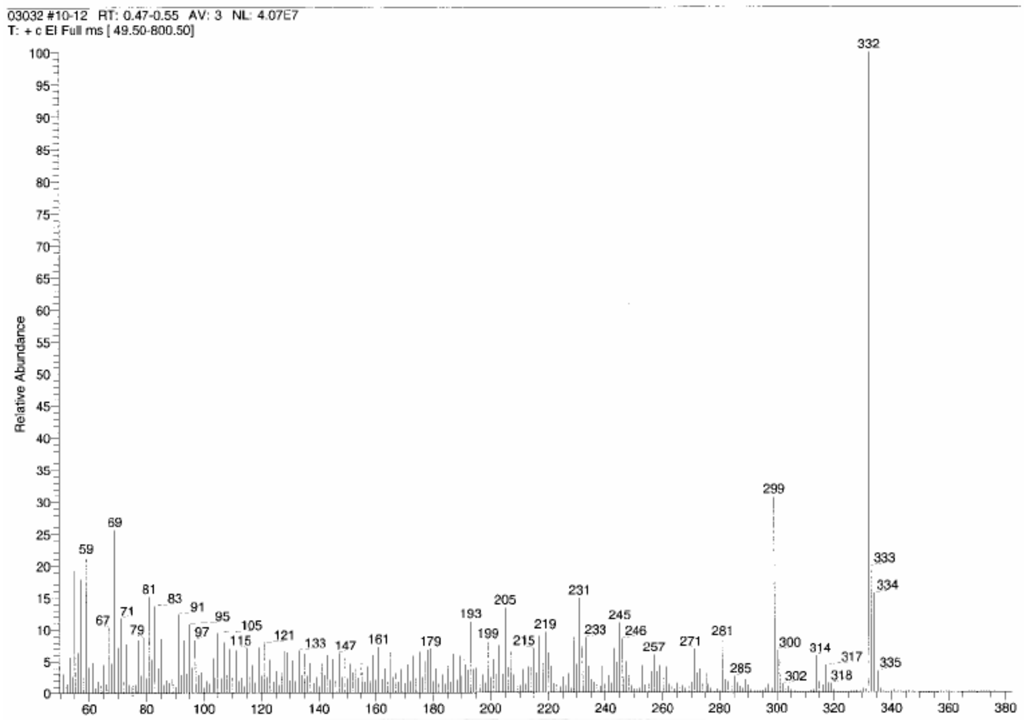

Figure S28. EI-MS spectrum of **4**

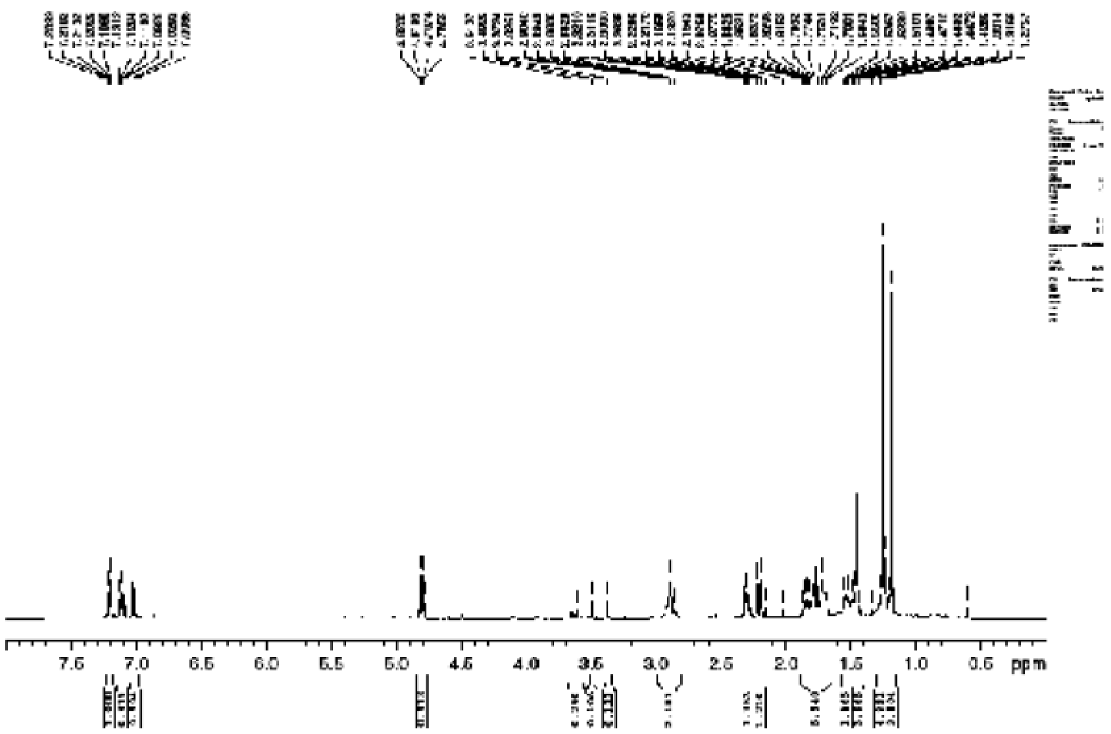

Figure S29. ^{1}H NMR spectrum of **5**

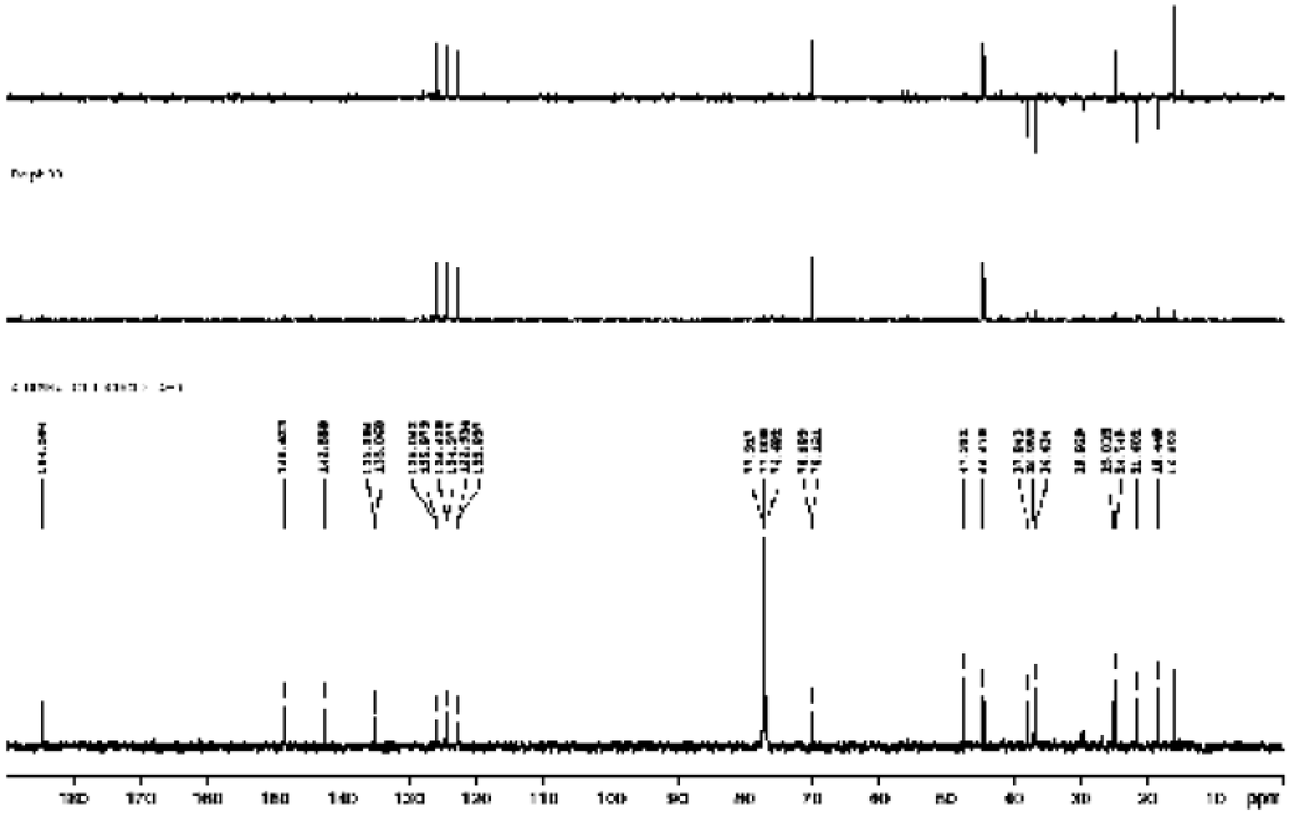

Figure S30. ^{13}C spectrum of **5**

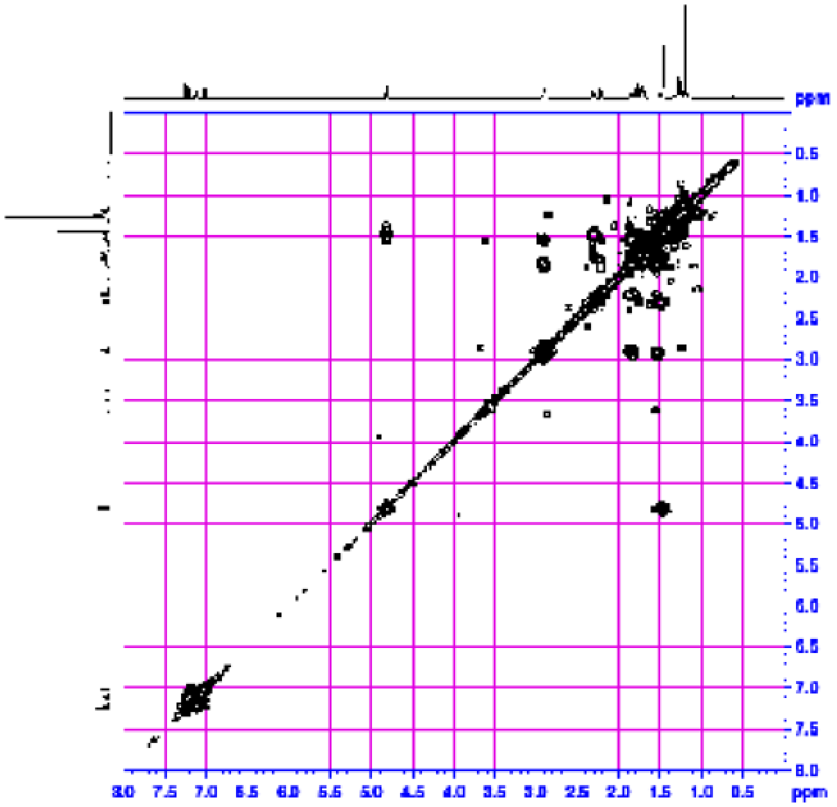

Figure S31. COSY spectrum of **5**

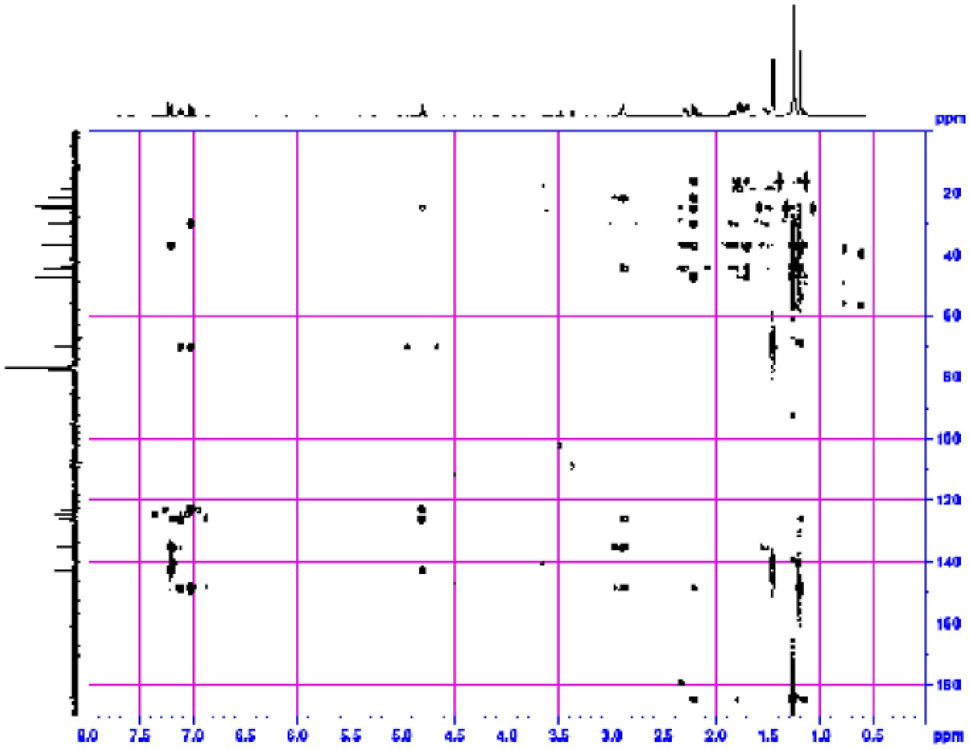

Figure S32. HMBC spectrum of **5**

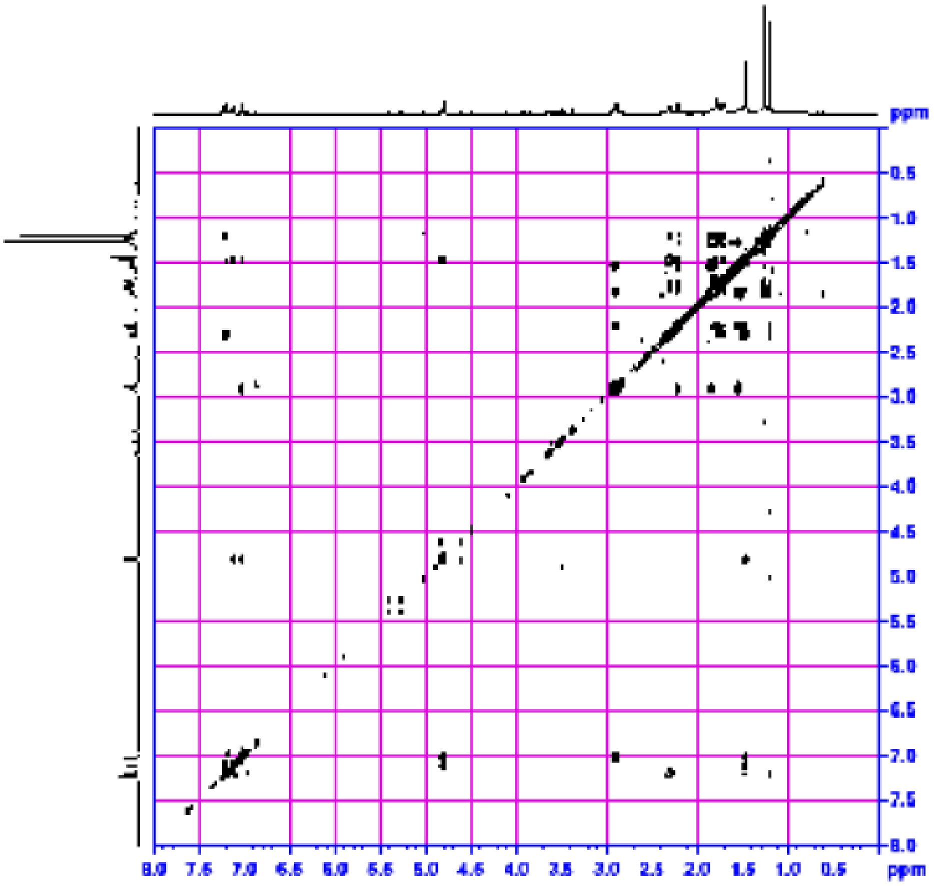

Figure S33. NOESY spectrum of **5**

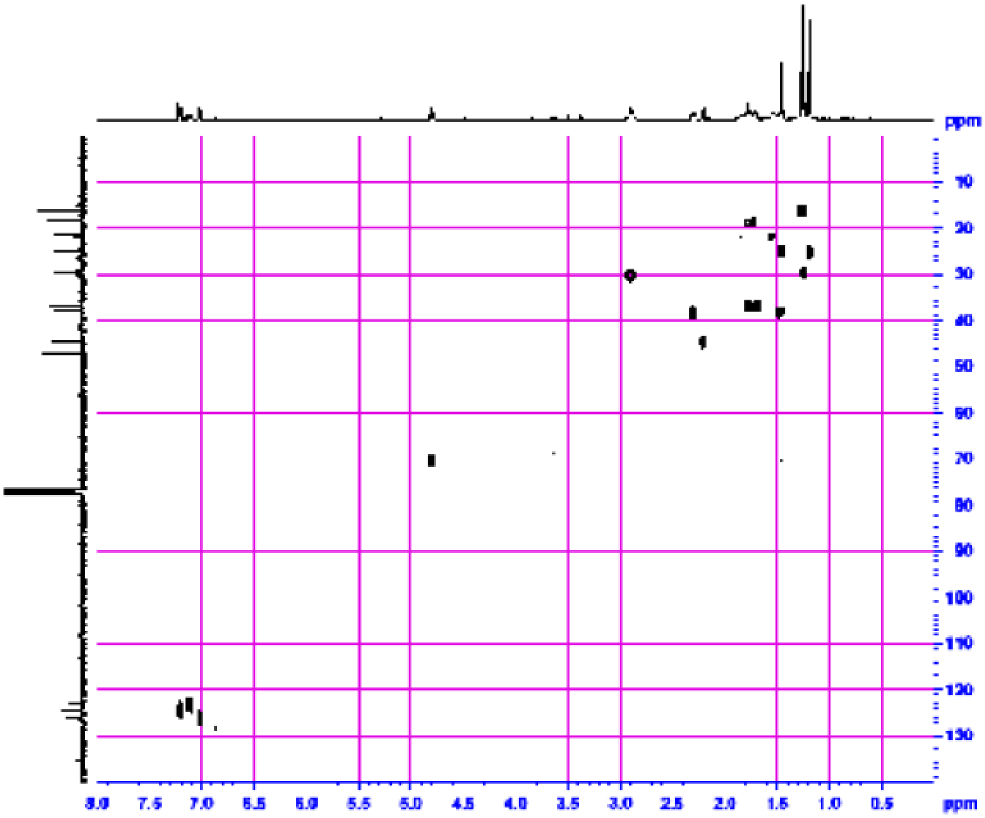

Figure S34. HSQC spectrum of **5**

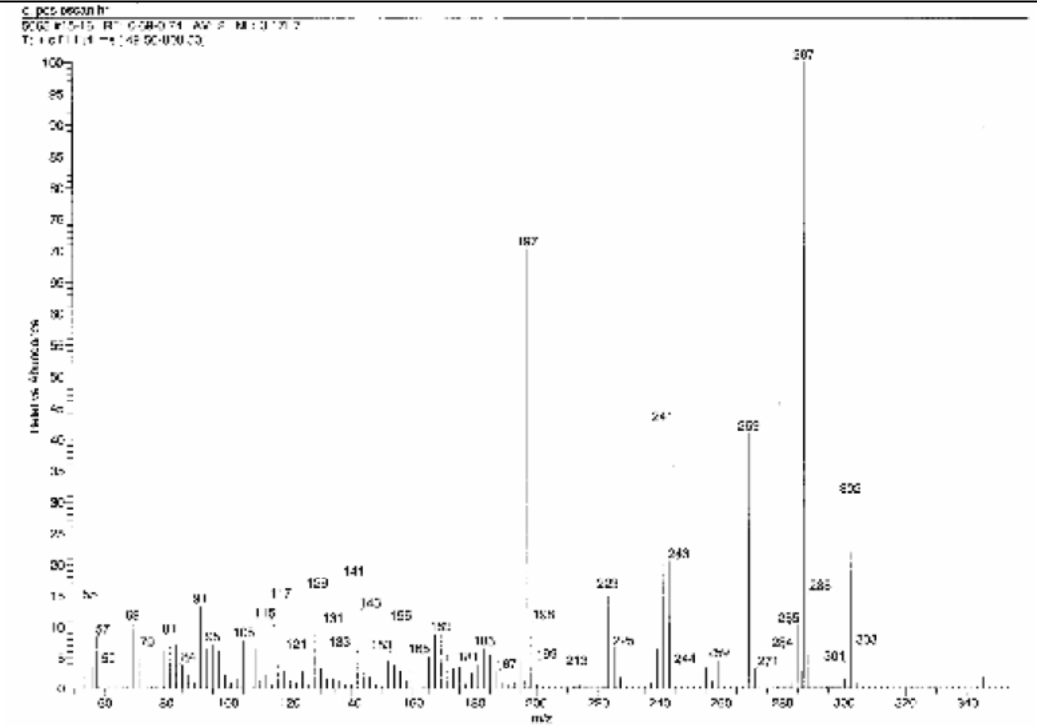

Figure S35. EI-MS spectrum of **5**

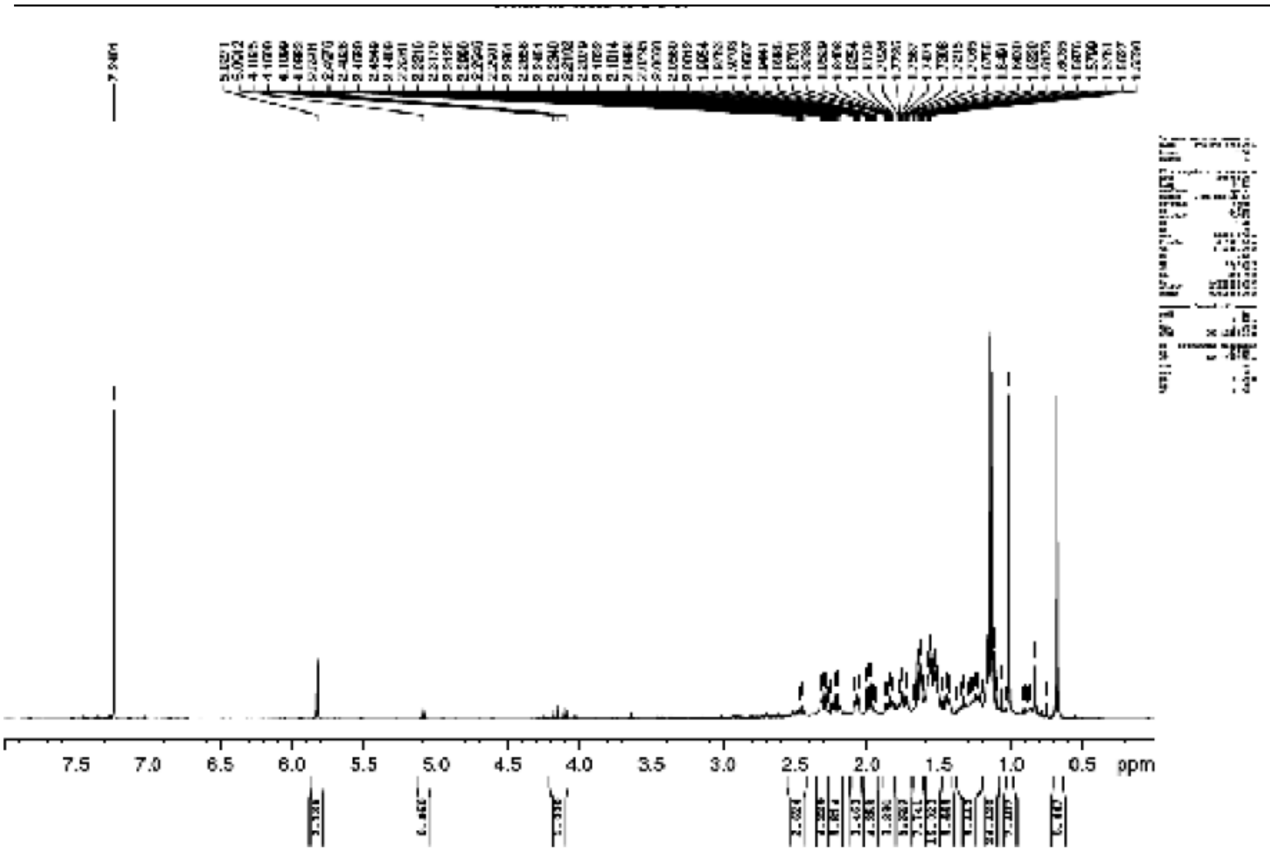

Figure S36. ^{1}H NMR spectrum of **6**

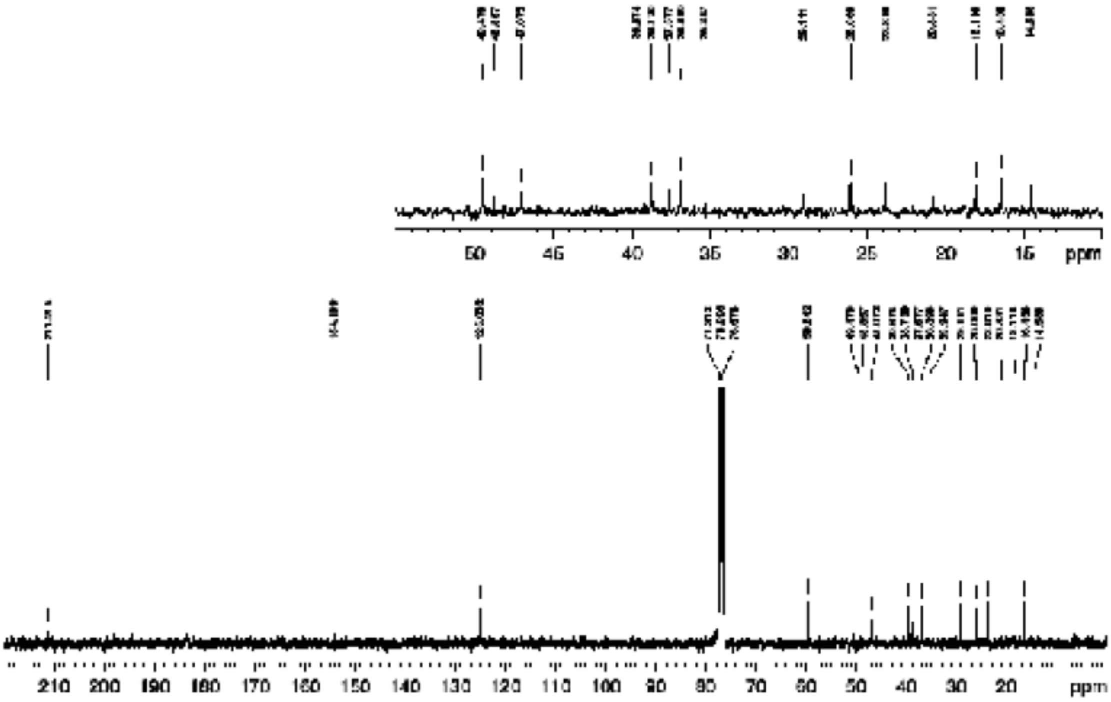

Figure S37. ^{13}C NMR/DEPT spectra of **6**

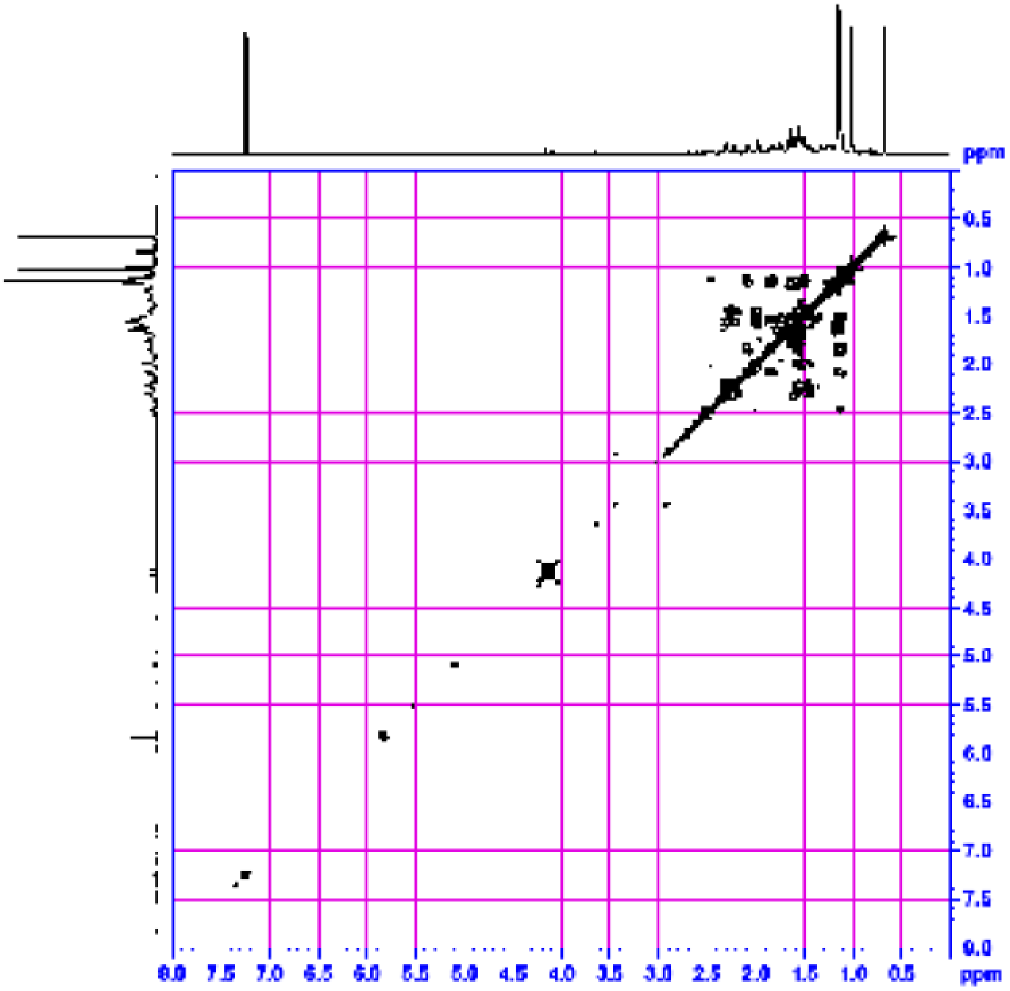

Figure S38. COSY spectrum of **6**

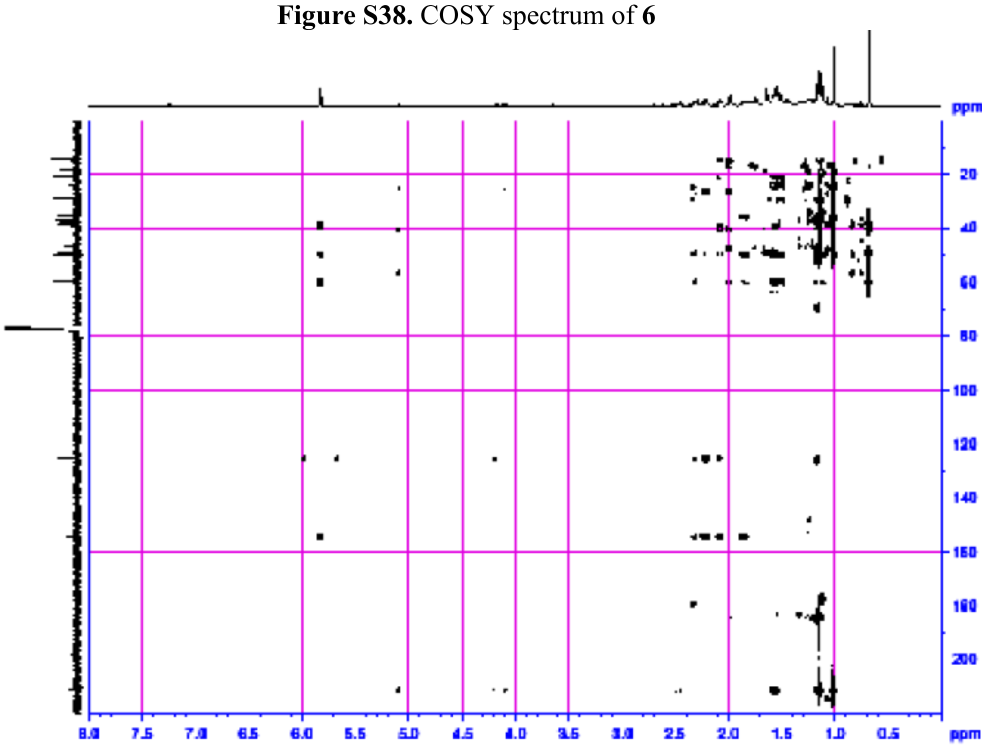

Figure S39. HMBC spectrum of **6**

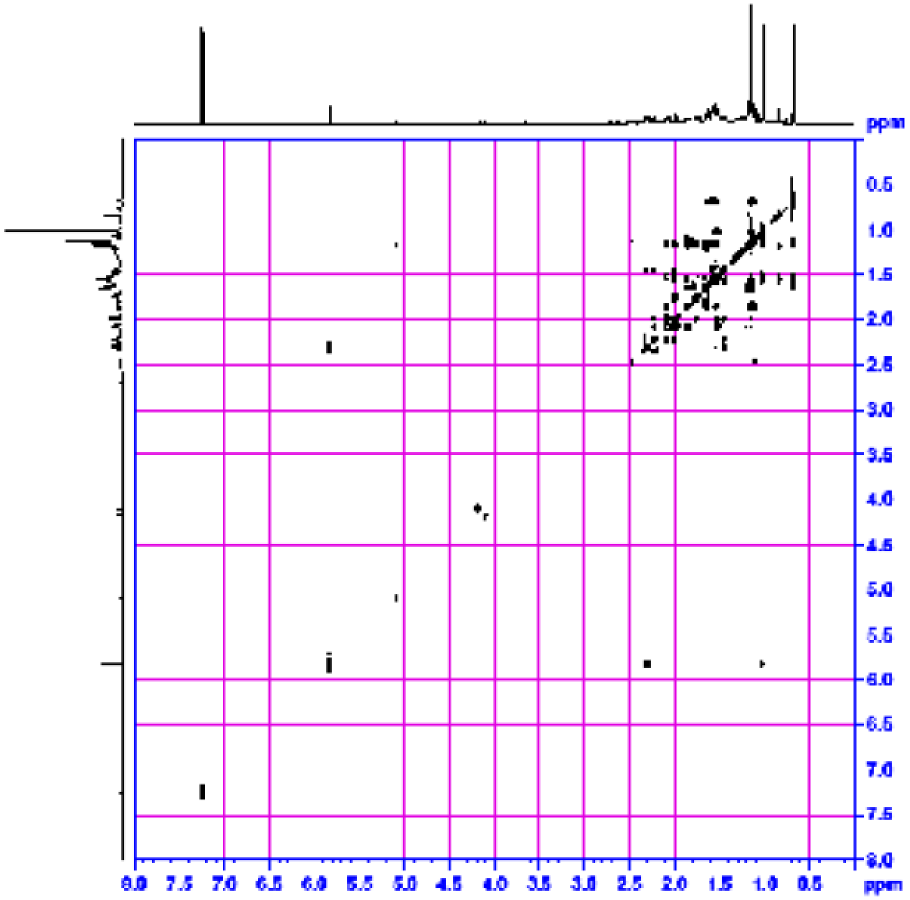

Figure S40. NOESY spectrum of **6**

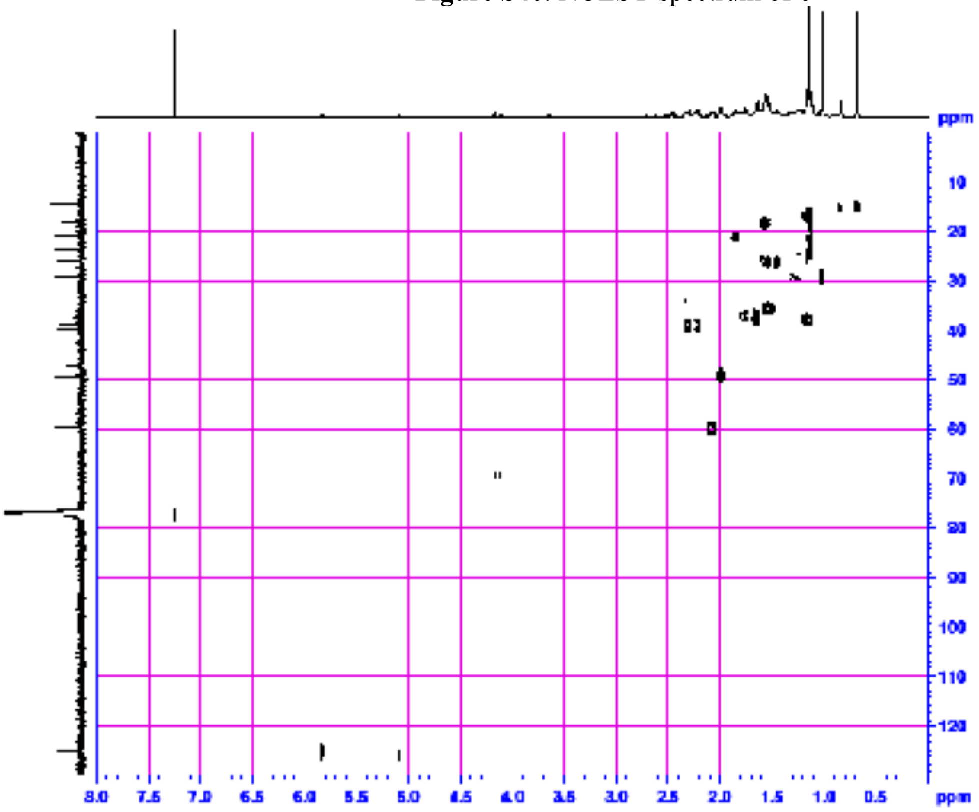

Figure S41. HSQC spectrum of **6**

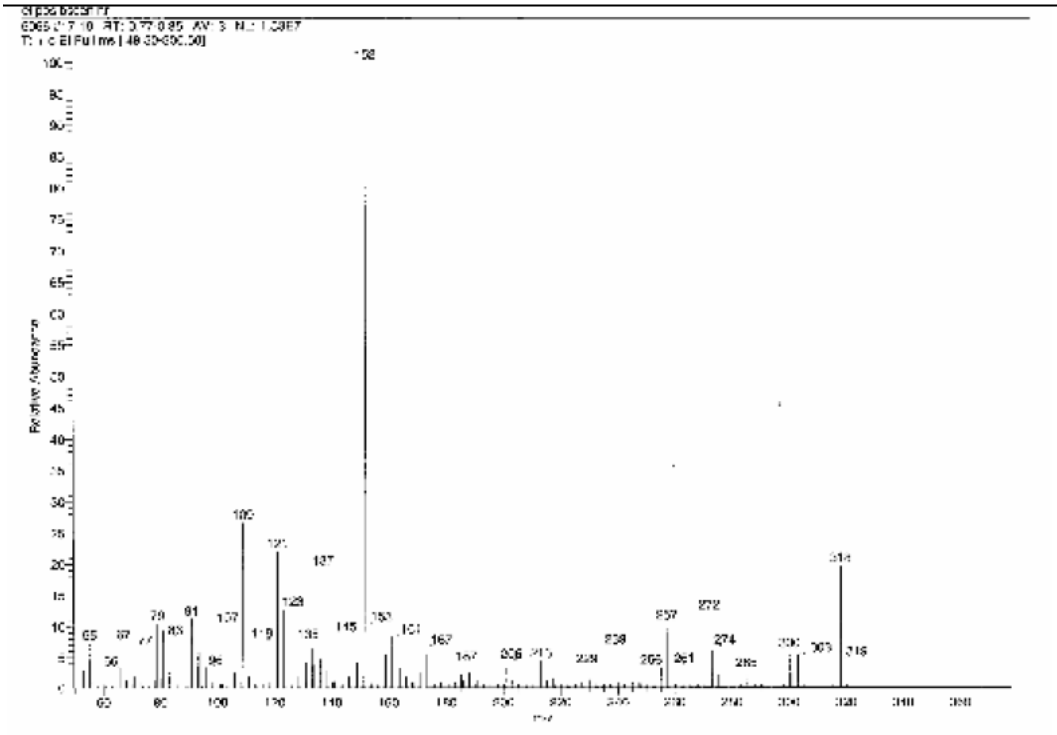

Figure S42. EI-MS spectrum of **6**

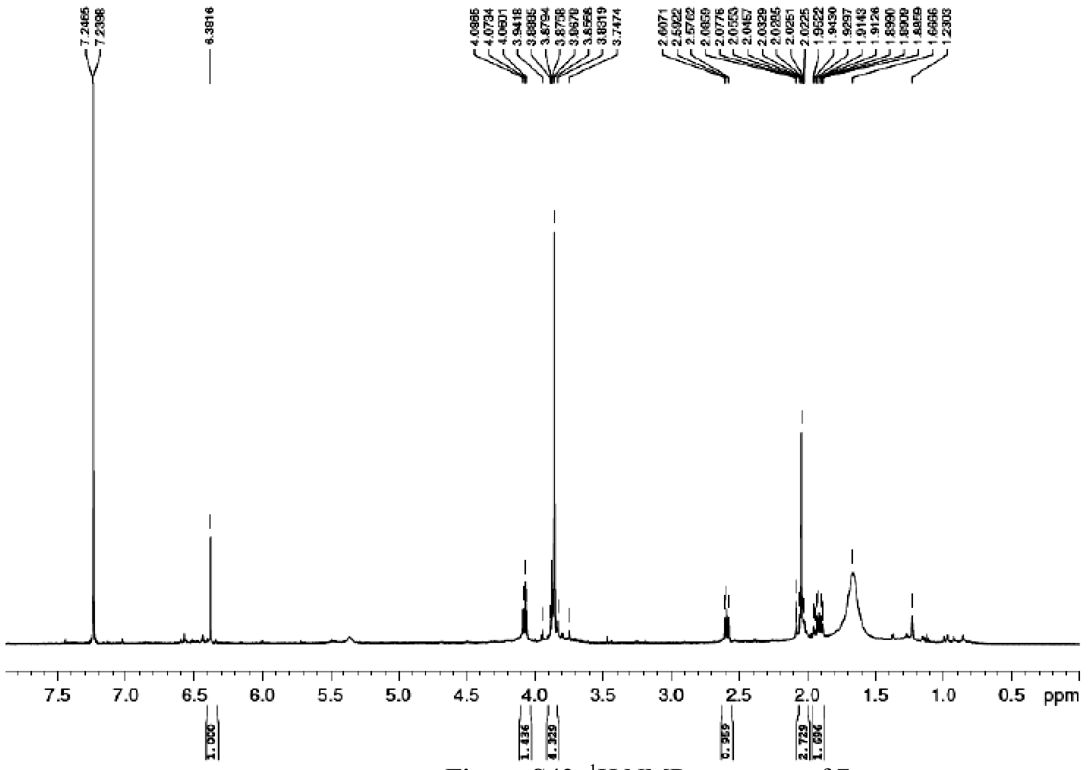

Figure S43. ^{1}H NMR spectrum of **7**

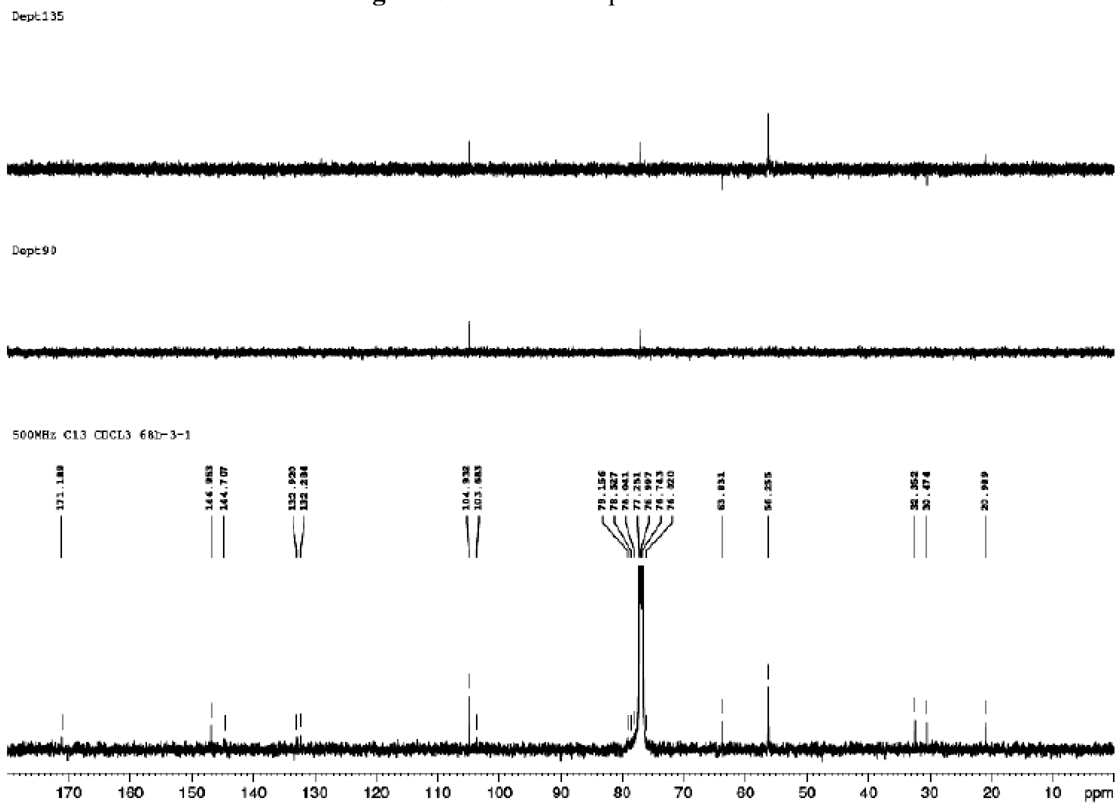

Figure S44. ^{13}C NMR/DEPT spectra of **7**

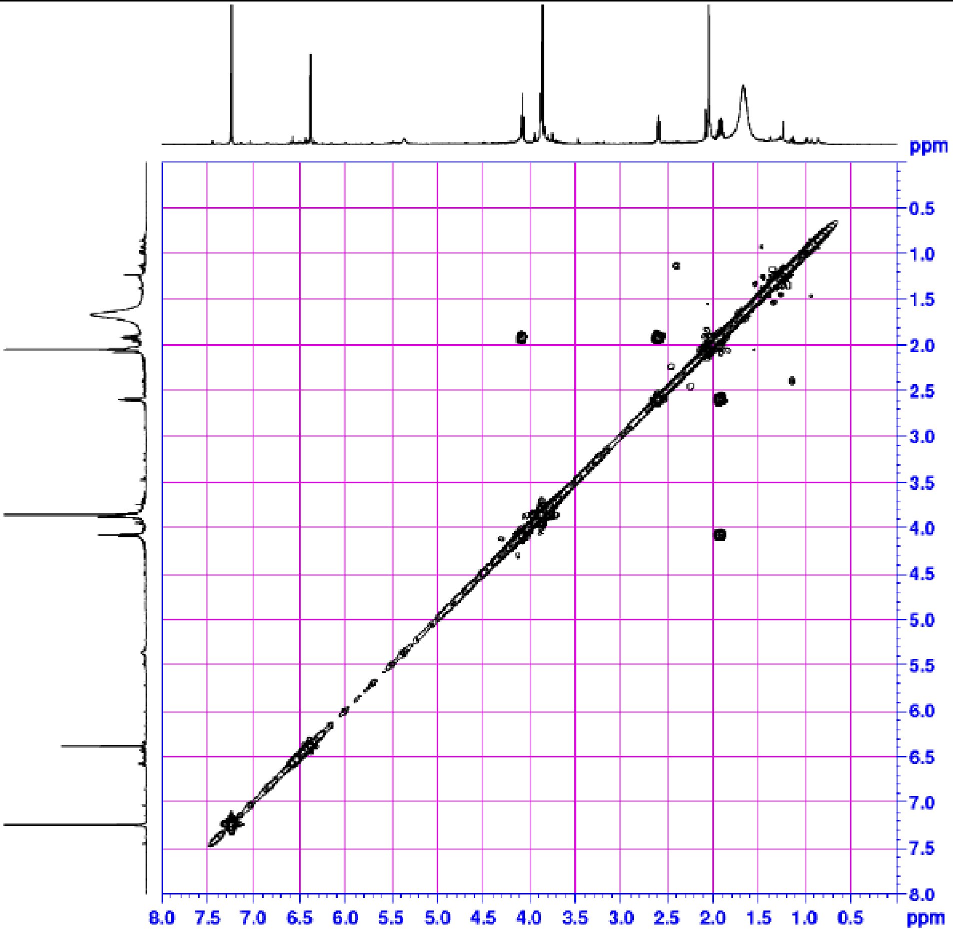

Figure S45. COSY spectrum of **7**

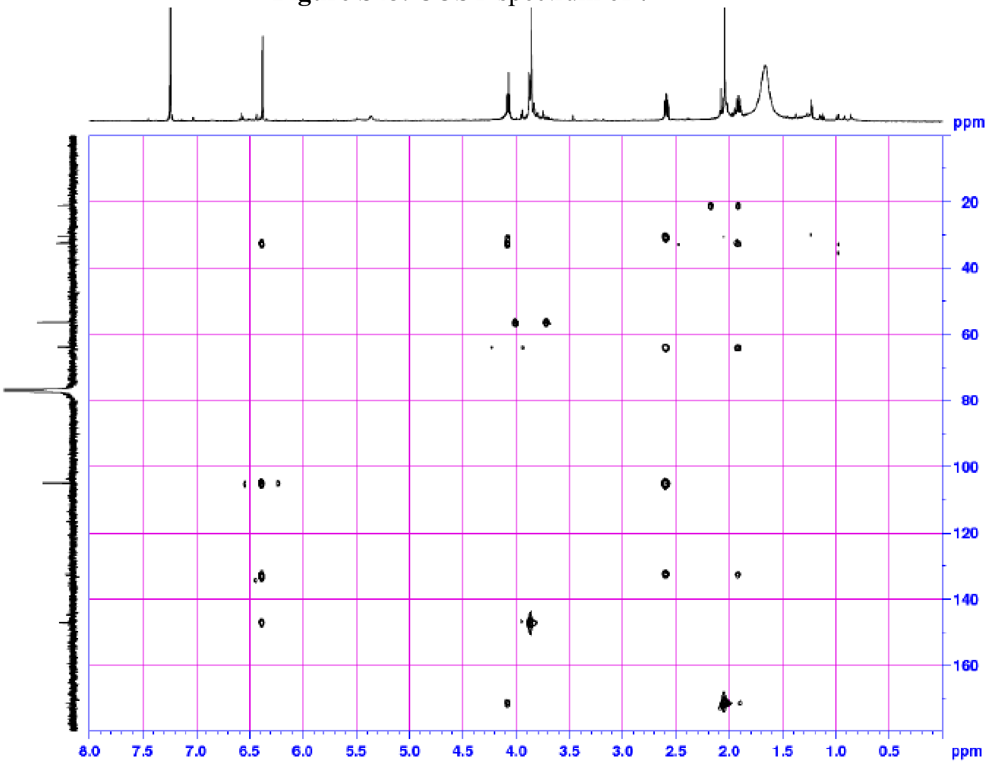

Figure S46. HMBC spectrum of **7**

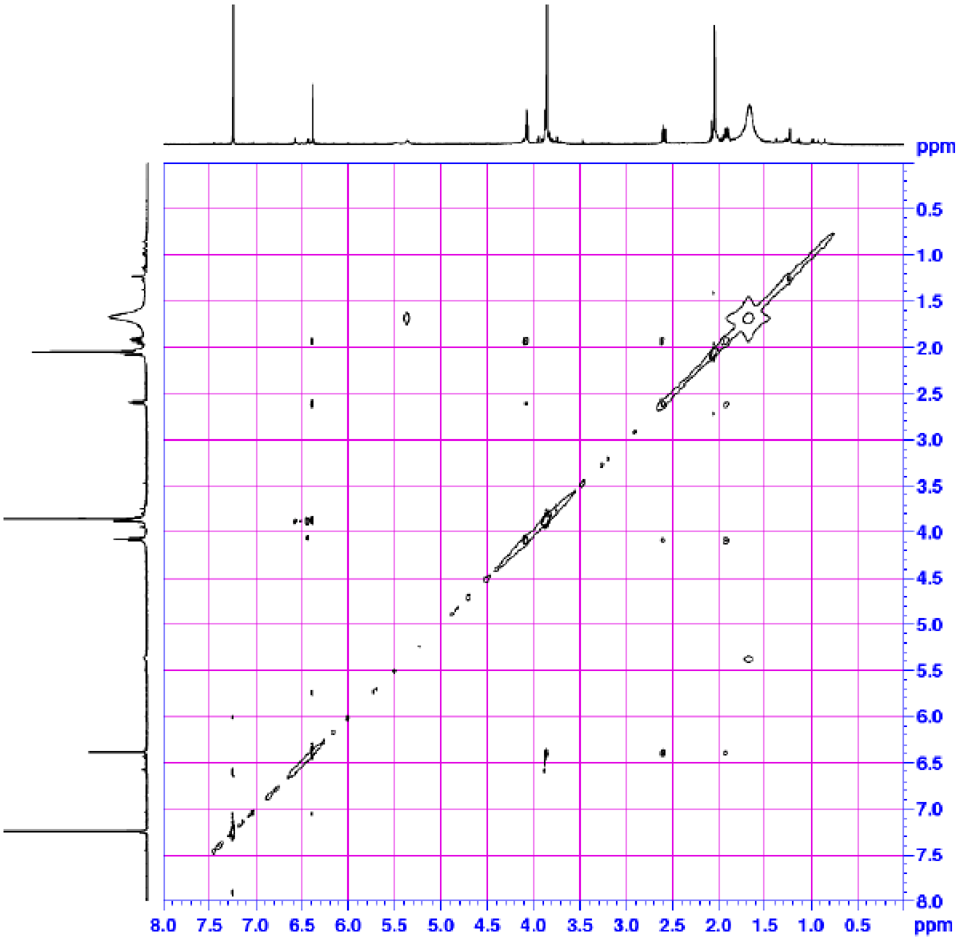

Figure S47. NOESY spectrum of **7**

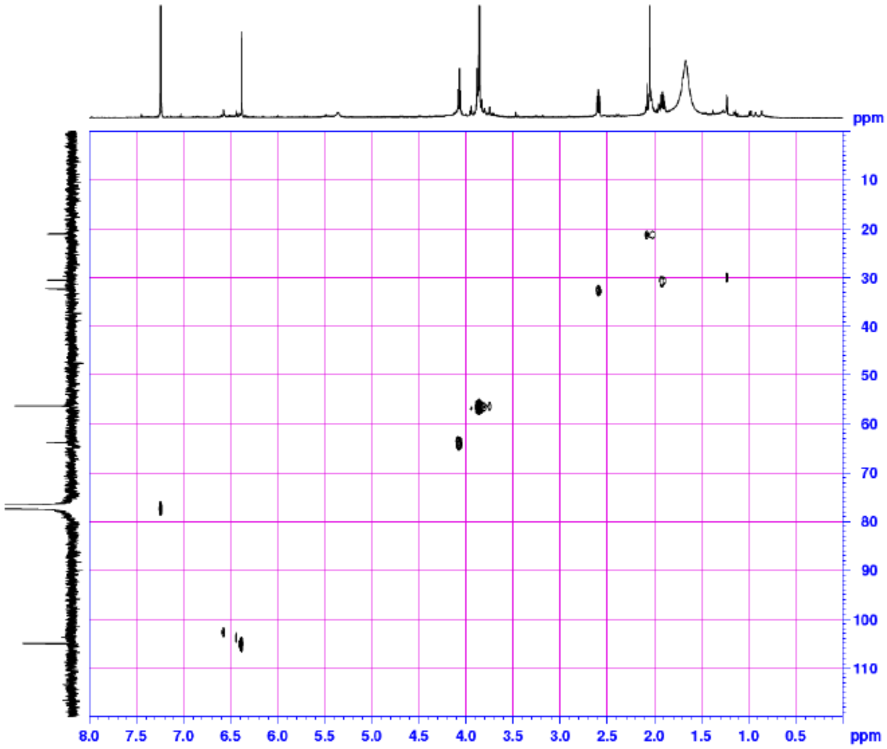

Figure S48. HSQC spectrum of **7**

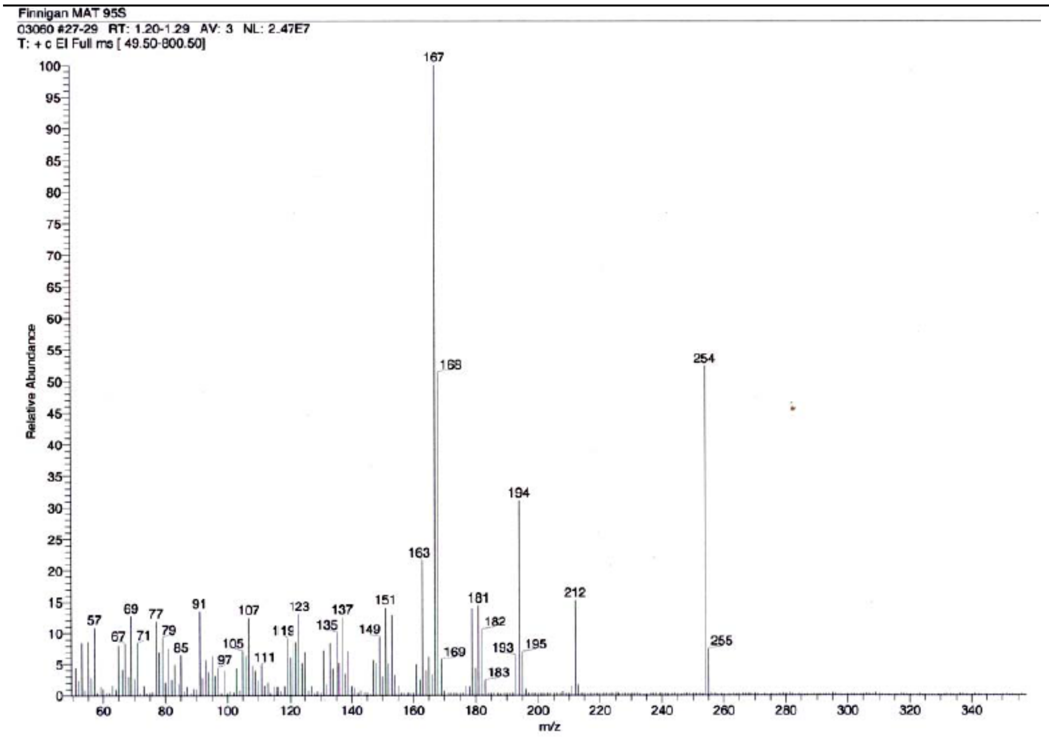

Figure S49. EI-MS spectrum of **7**

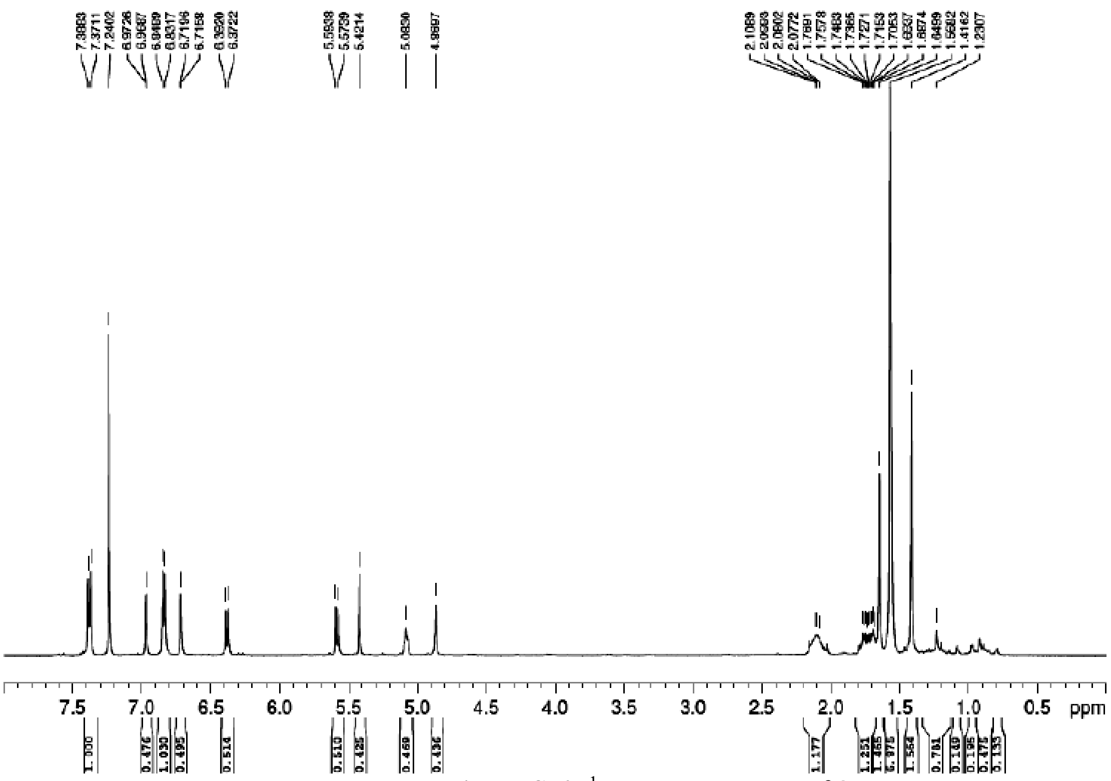

Figure S50. ^{1}H NMR spectrum of **8**

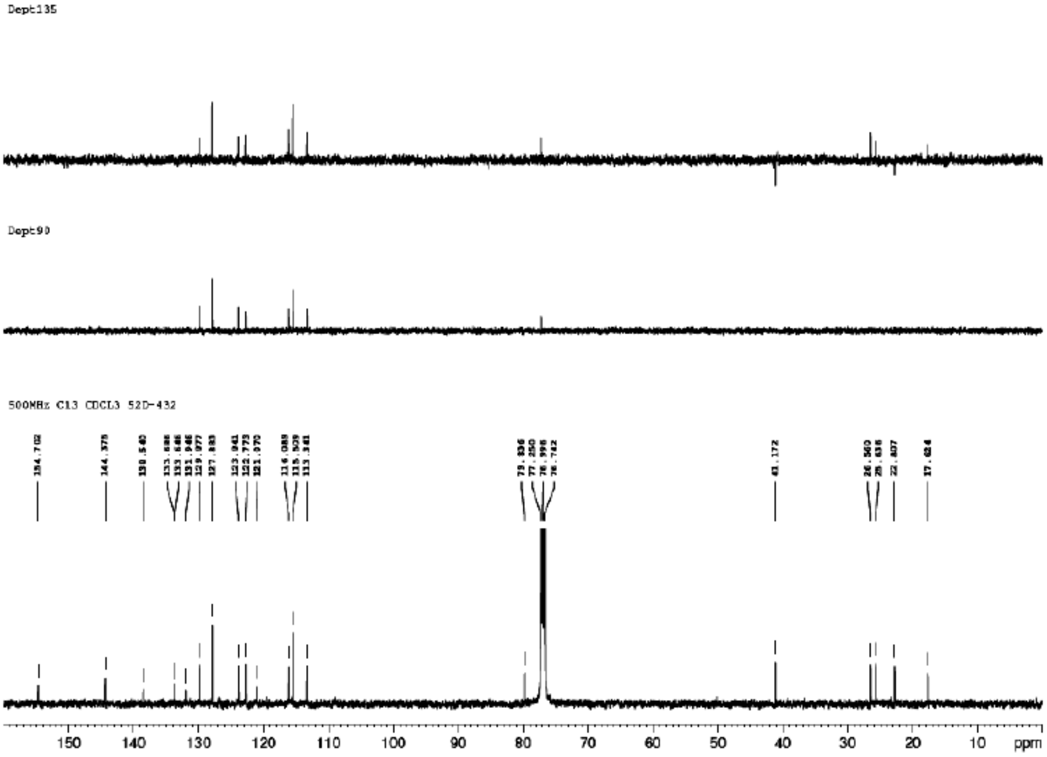

Figure S51. ^{13}C NMR/DEPT spectra of **8**

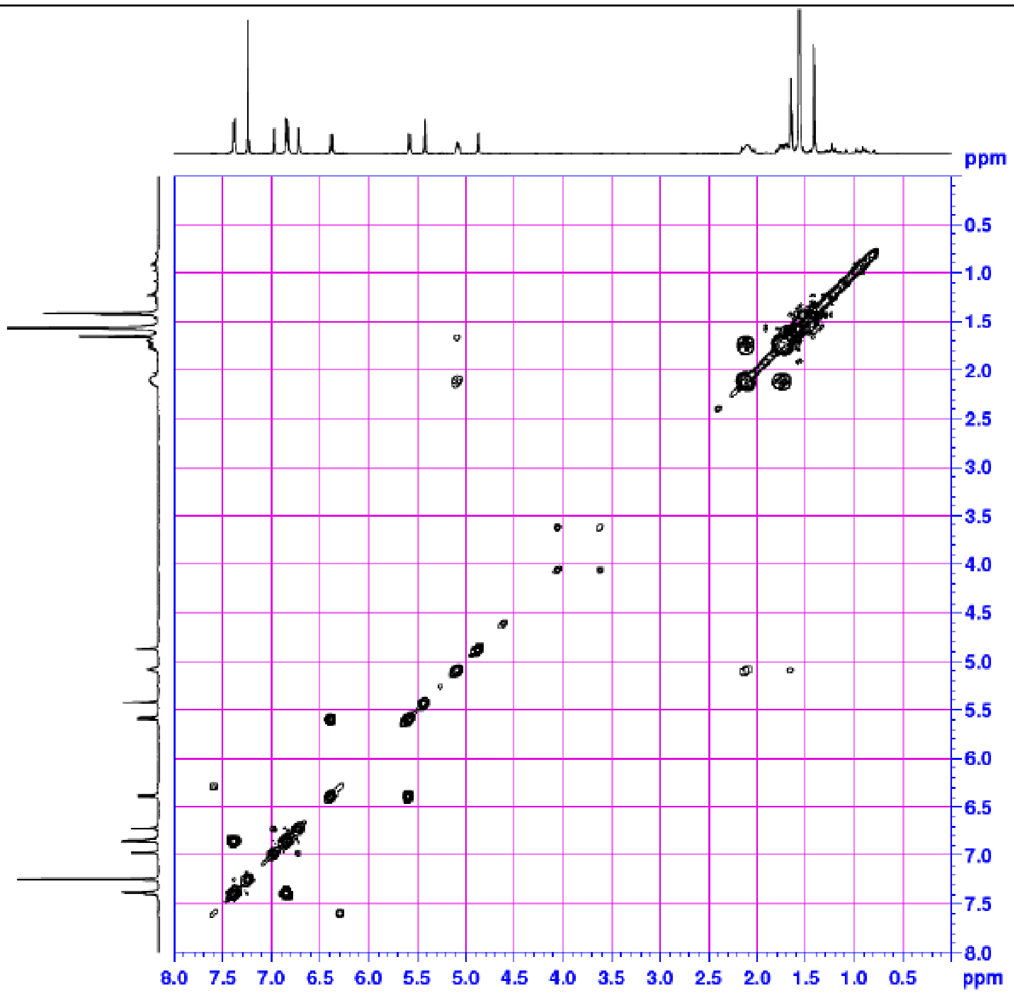

Figure S52. COSY spectrum of **8**

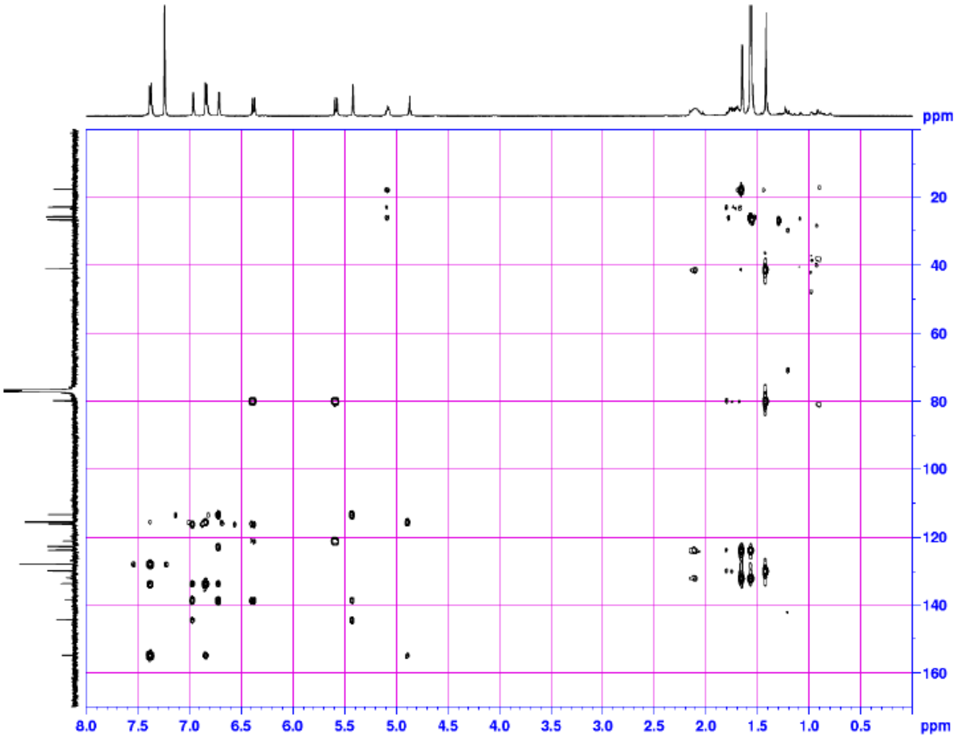

Figure S53. HMBC spectrum of **8**

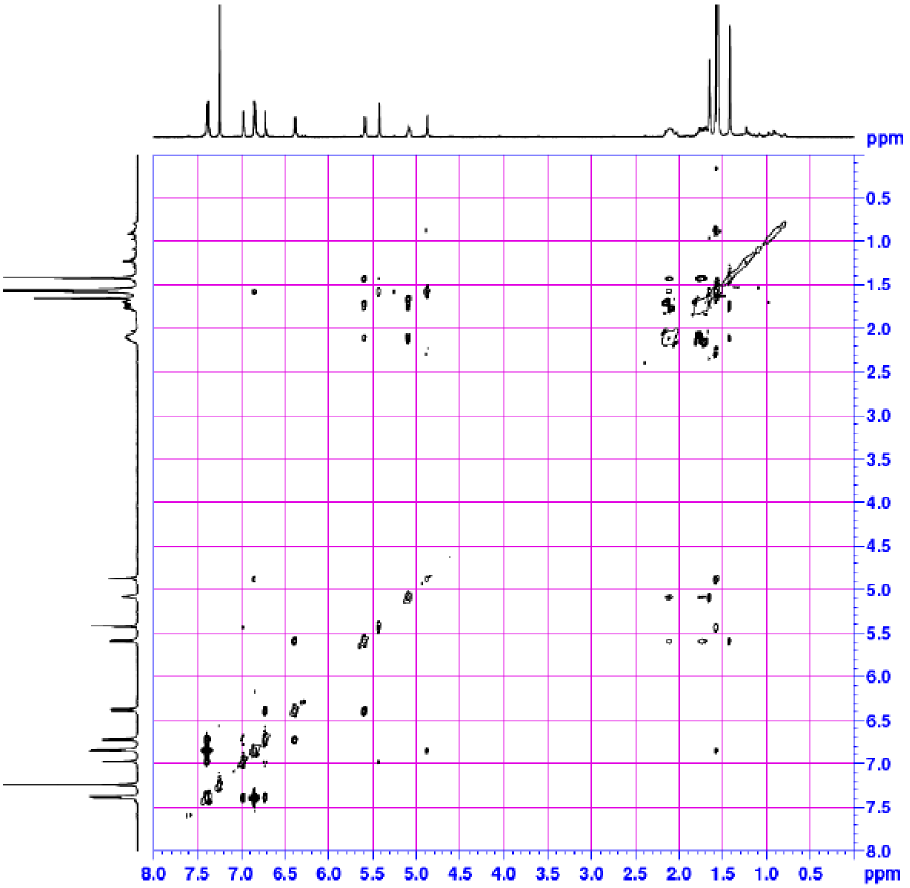

Figure S54. NOESY spectrum of **8**

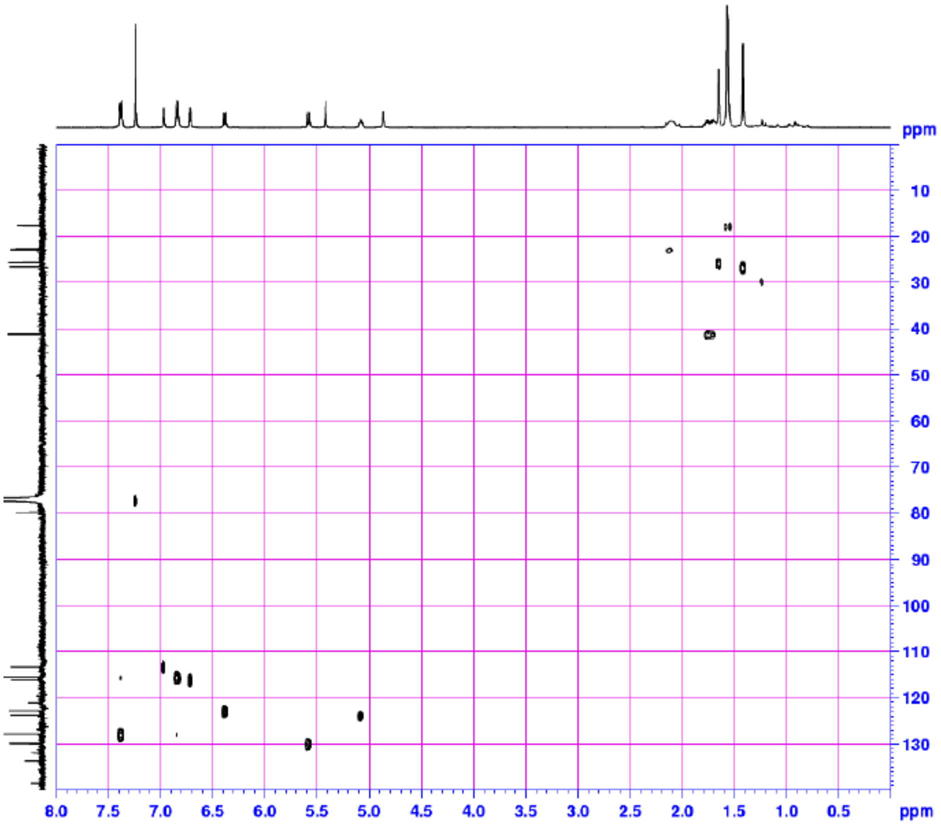

Figure S55. HSQC spectrum of **8**

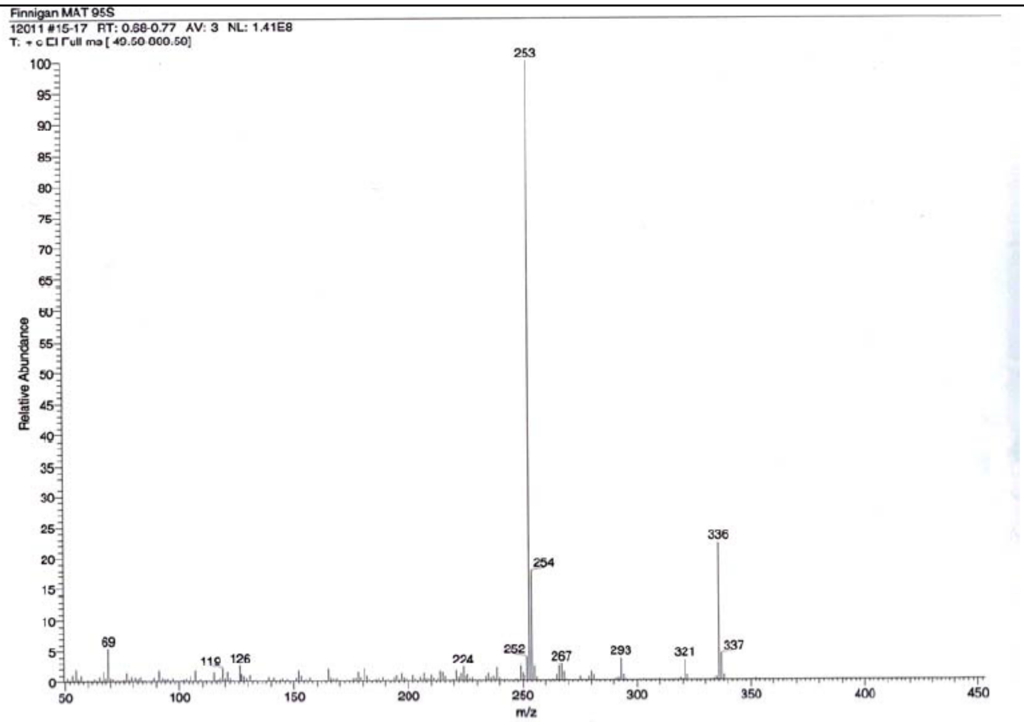

Figure S56. EI-MS spectrum of **8**

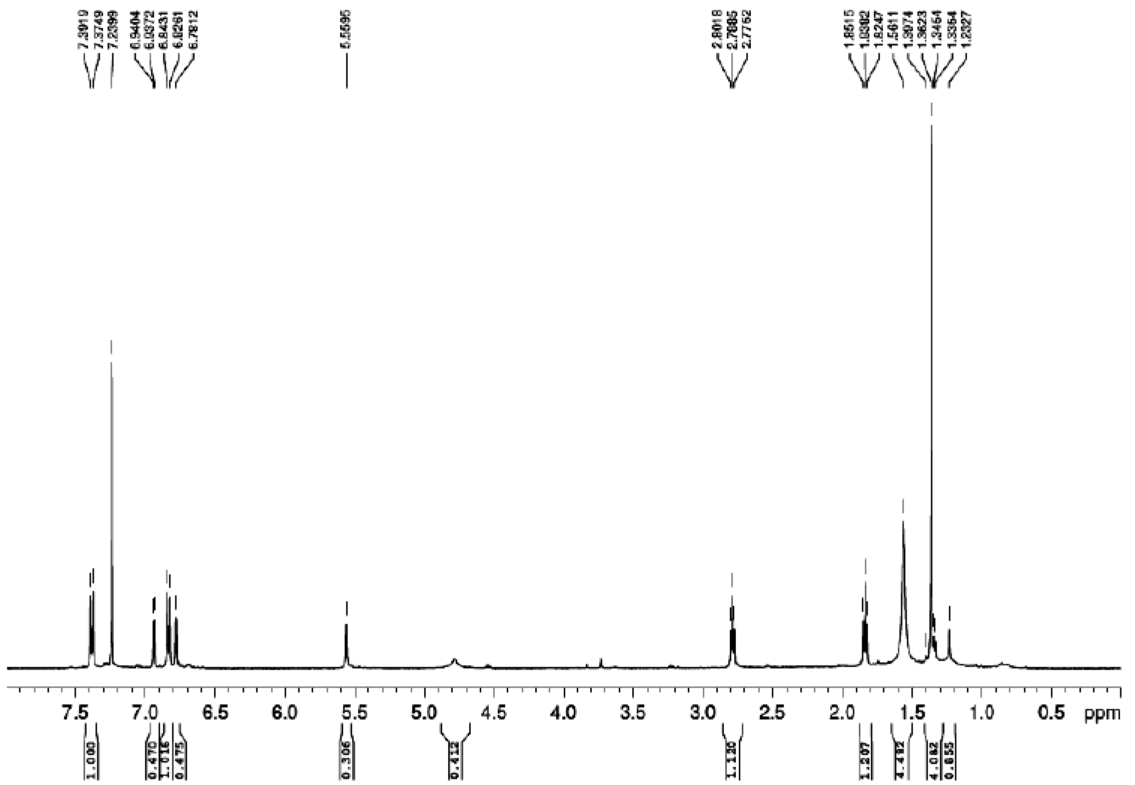

Figure S57. ^{1}H NMR spectrum of **9**

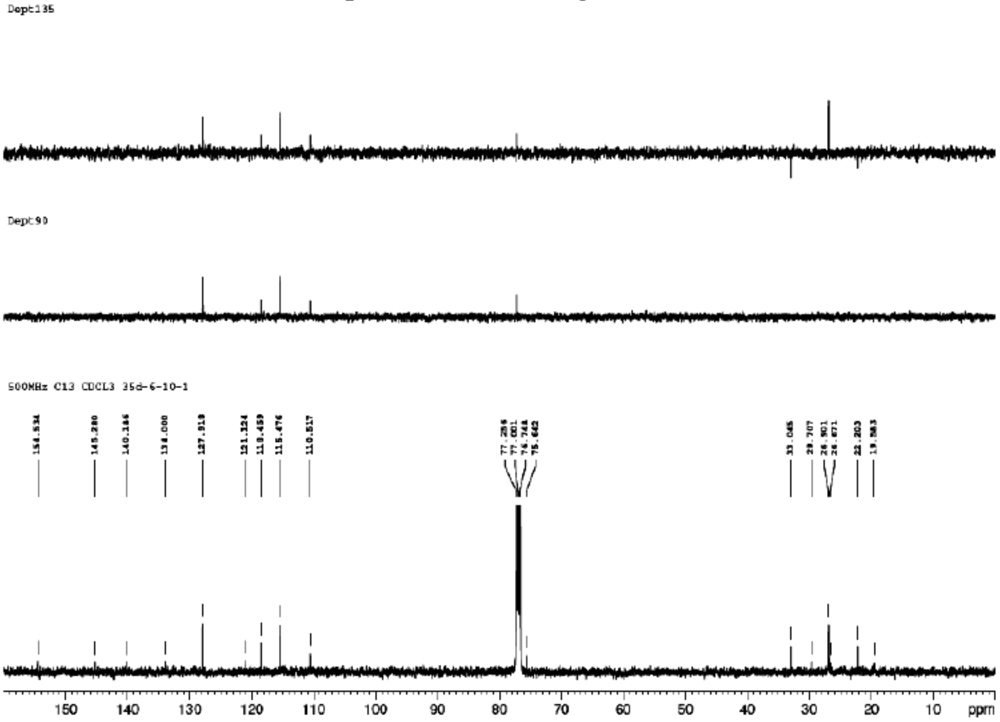

Figure S58. ^{13}C NMR/DEPT spectra of **9**

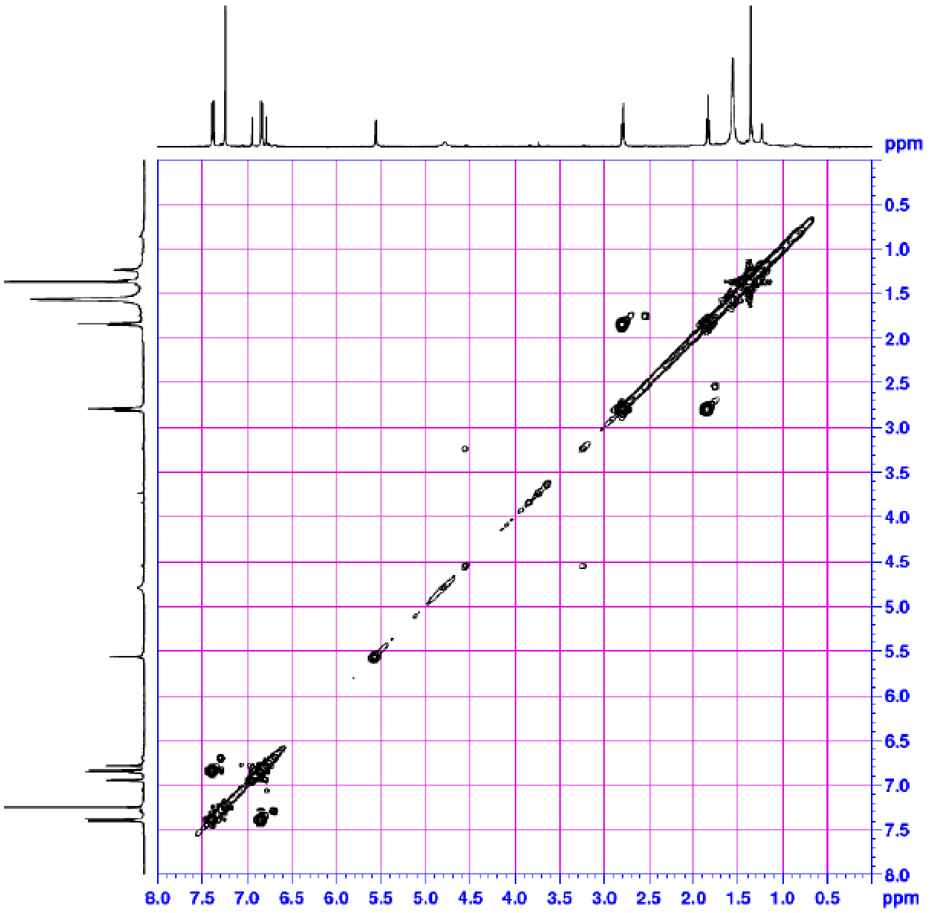

Figure S59. COSY spectrum of **9**

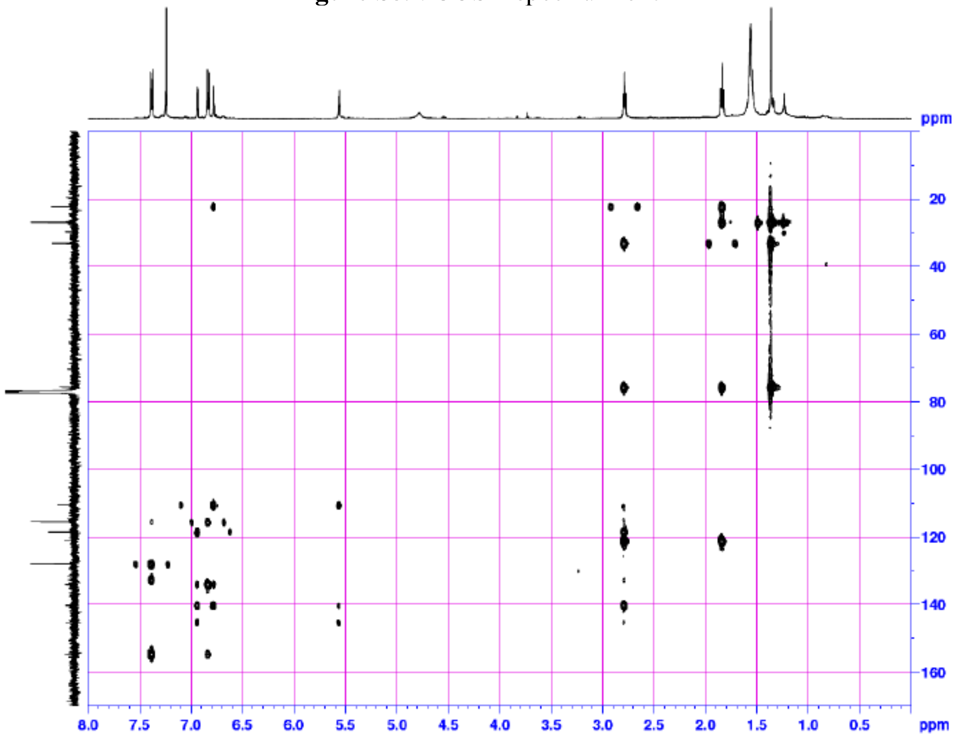

Figure S60. HMBC spectrum of **9**

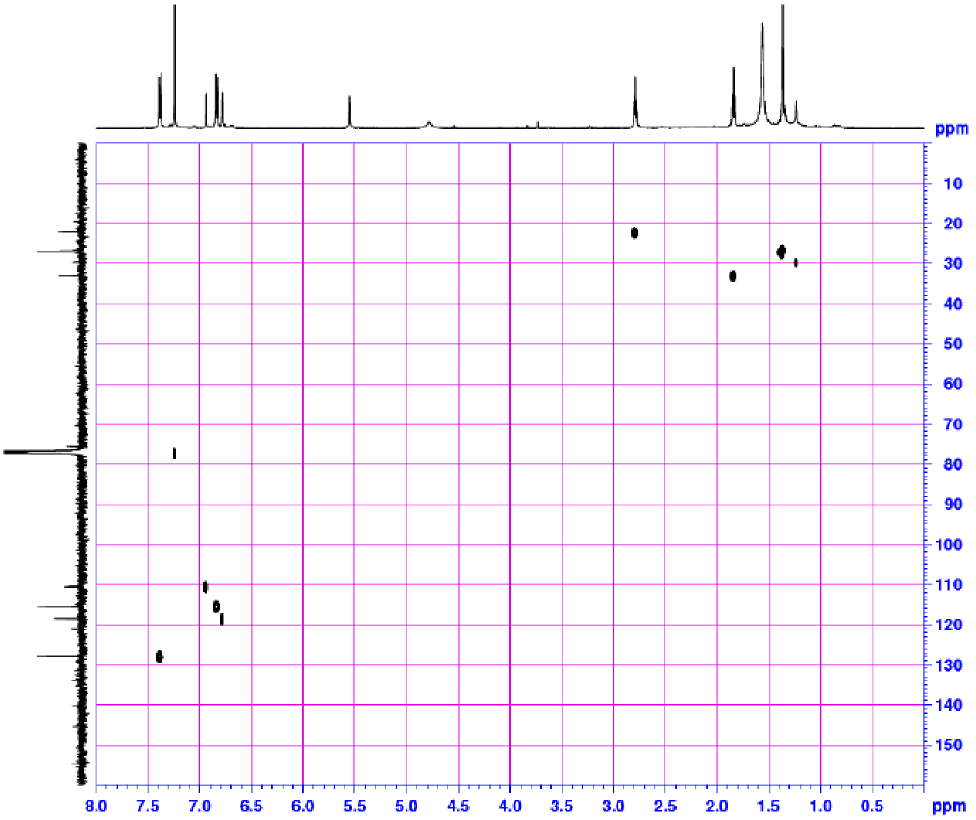

Figure S61. NOESY spectrum of **9**

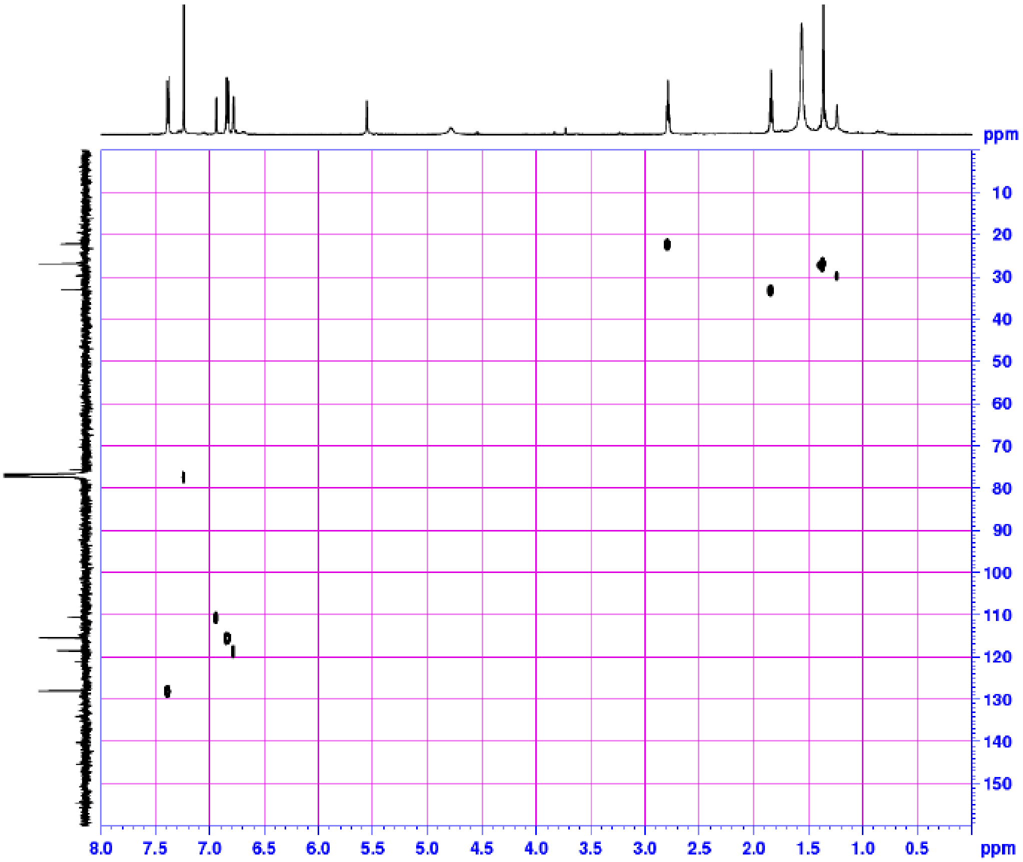

Figure S62 HSQC spectrum of **9**

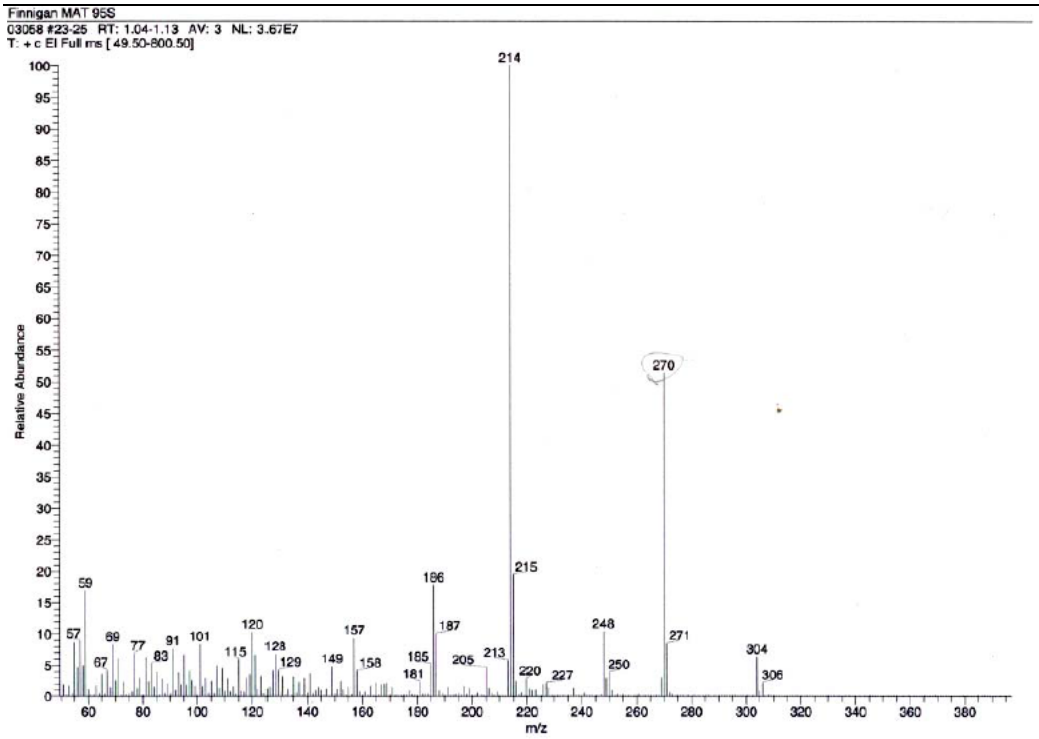

Figure S63. EI-MS spectrum of **9**

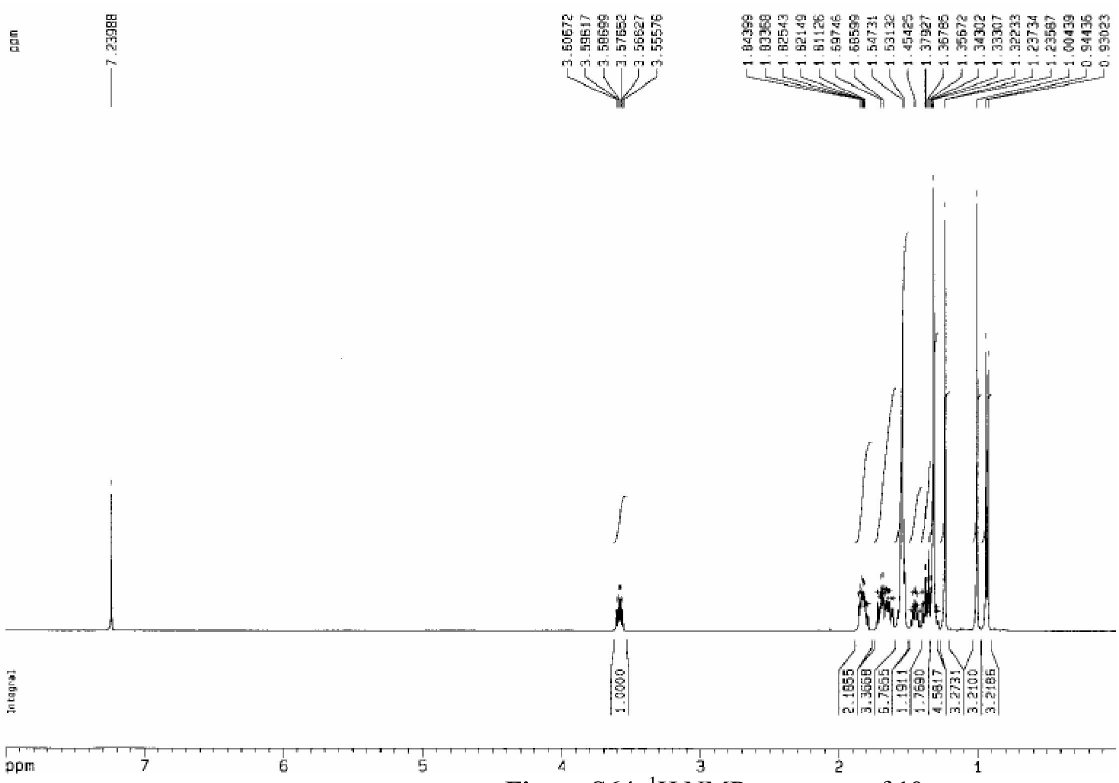

Figure S64. ^{1}H NMR spectrum of **10**

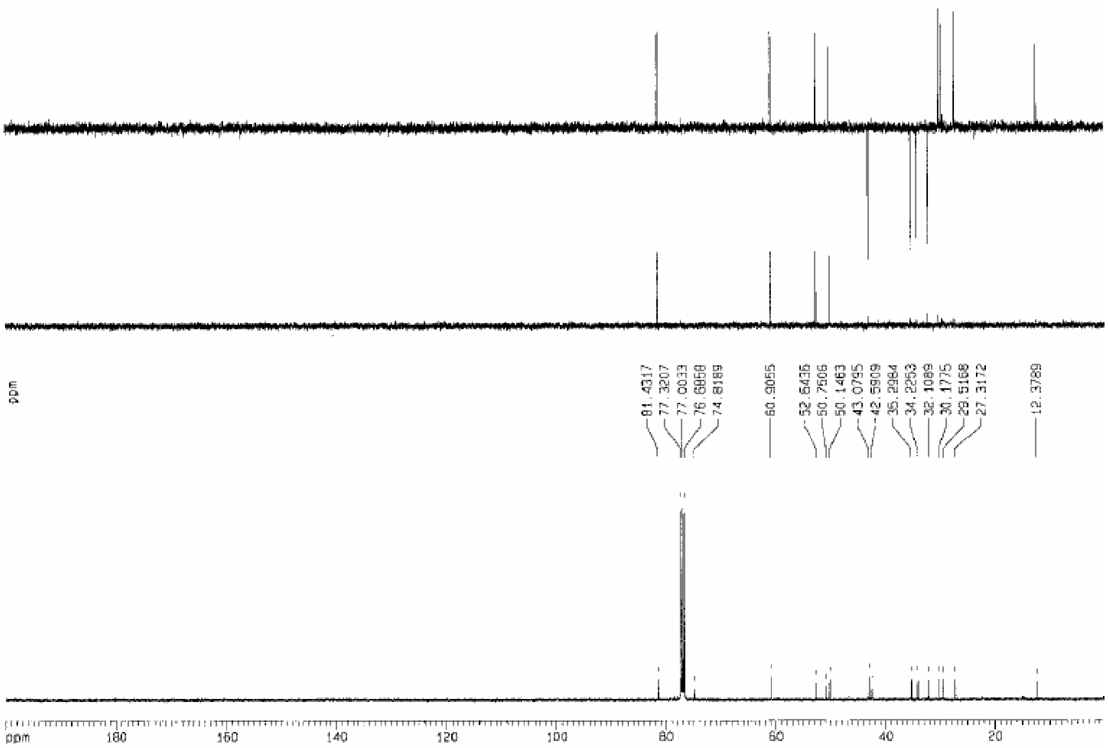

Figure S65. ^{13}C NMR/DEPT spectra of **10**

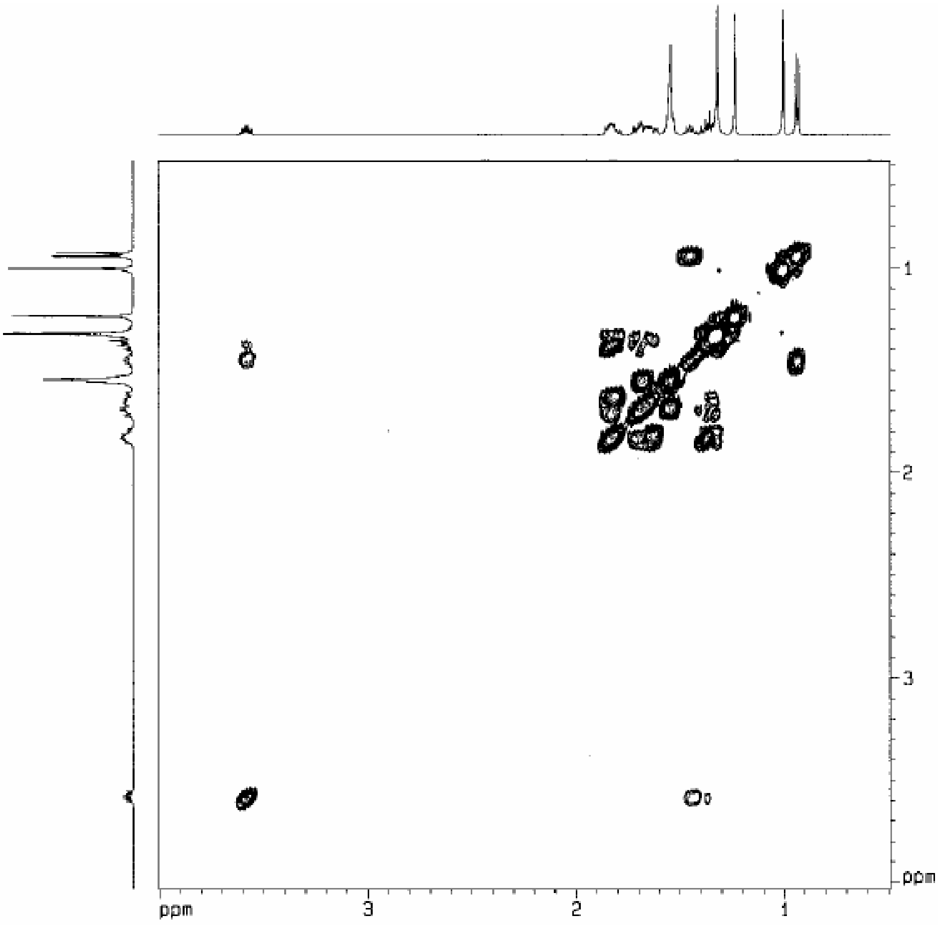

Figure S66. COSY spectrum of **10**

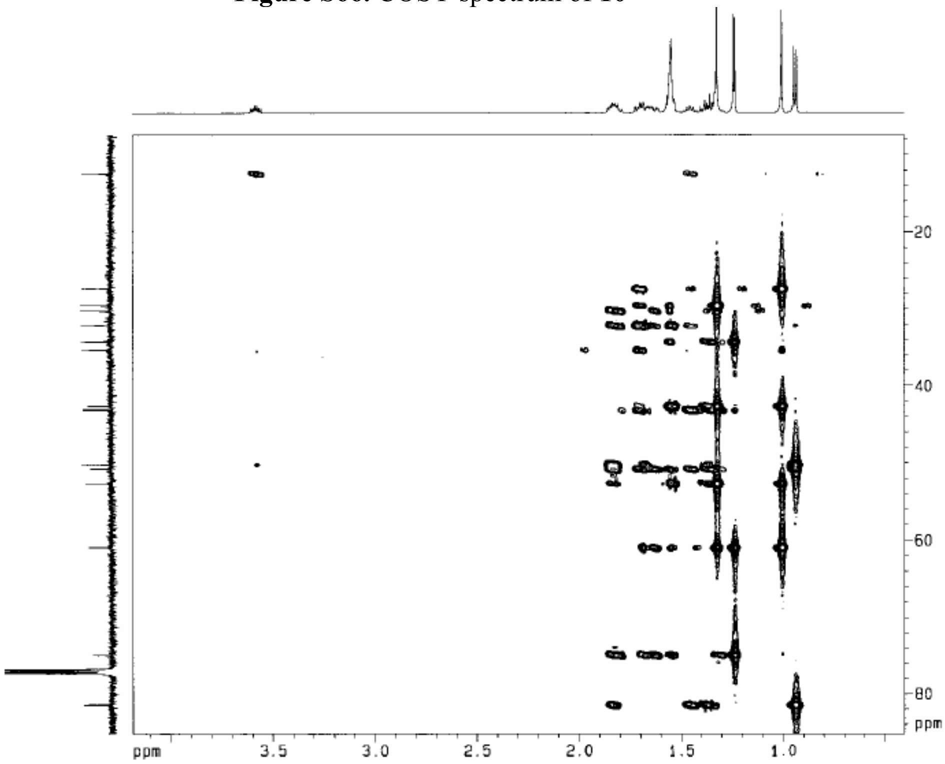

Figure S67. HMBC spectrum of **10**

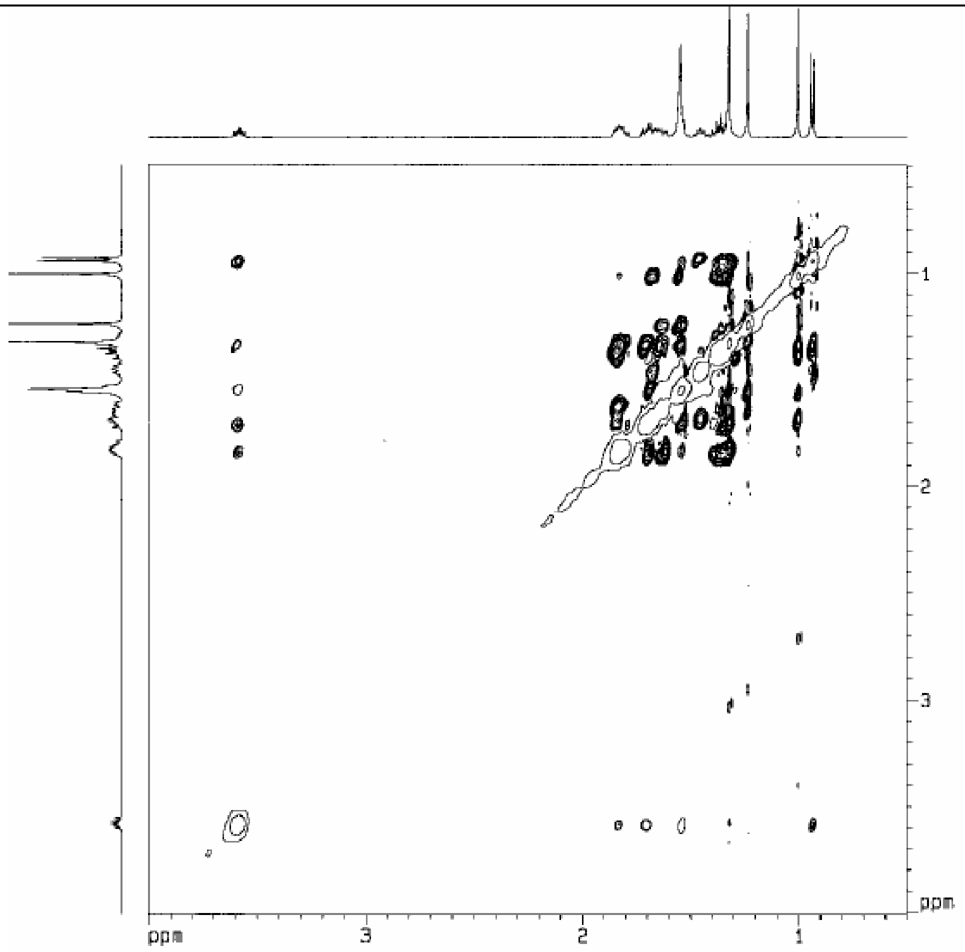

Figure S68 NOESY spectrum of **10**

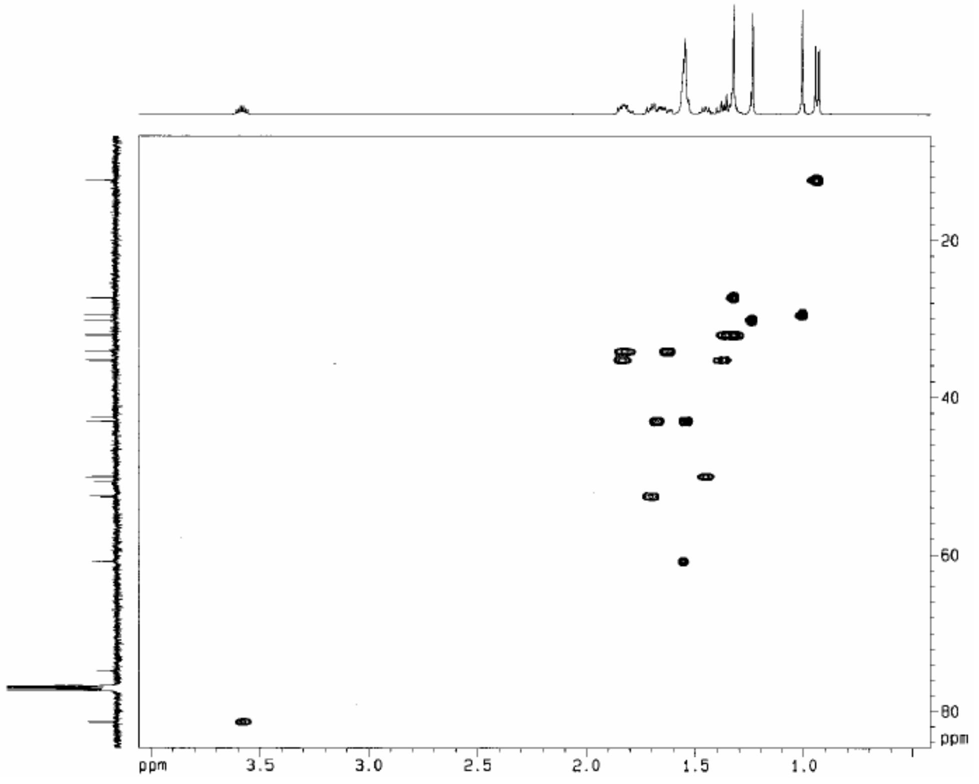

Figure S69. HSQC spectrum of **10**

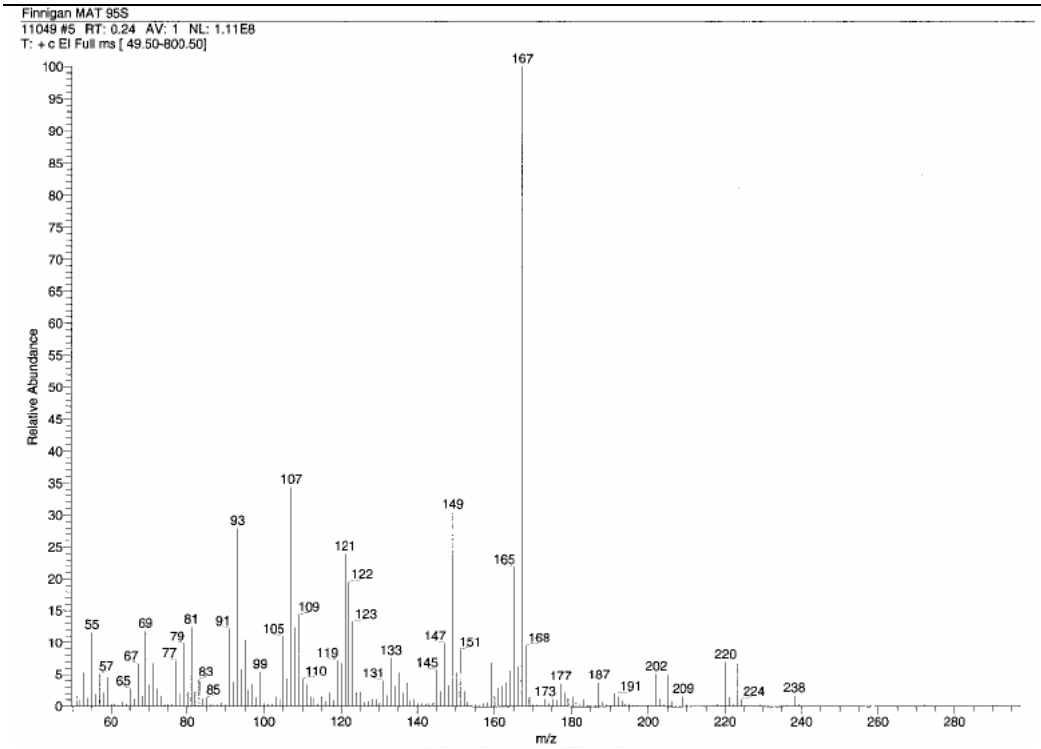

Figure S70. EI-MS spectrum of **10**
